# Theoretical Investigation of Energetic Salts with Pentazolate Anion

**DOI:** 10.3390/molecules25081783

**Published:** 2020-04-13

**Authors:** Hao-Ran Wang, Chong Zhang, Bing-Cheng Hu, Xue-Hai Ju

**Affiliations:** School of Chemical Engineering, Nanjing University of Science and Technology, Nanjing 210094, China; hrwang6@163.com (H.-R.W.); czhang@njust.edu.cn (C.Z.)

**Keywords:** energetic salts, density functional theory, vertical electron affinity, vertical ionization potential, density, pentazolate anion

## Abstract

Energetic salts based on pentazolate anion (*cyclo*-N_5_^−^) have attracted much attention due to their high nitrogen contents. However, it is an enormous challenge to efficiently screen out an appropriate cation that can match well with *cyclo*-N_5_^−^. The vertical electron affinity (*VEA*) of the cations and vertical ionization potential (*VIP*) of the anions for 135 energetic salts and some *cyclo*-N_5_^−^ salts were calculated by the density functional theory (DFT). The magnitudes of *VEA* and *VIP*, and their matchability were analyzed. The results based on the calculations at the B3LYP/6-311++G(d,p) and B3LYP/aug-cc-pVTZ levels indicate that there is an excellent compatibility between *cyclo*-N_5_^−^ and cation when the difference between the *VEA* of cation and the *VIP* of *cyclo*-N_5_^−^ anion is −2.8 to −1.0 eV. The densities of the salts were predicted by the DFT method. Relationship between the calculated density and the experimental density was established as *ρ*_Expt_ = 1.111*ρ*_cal_ − 0.06067 with a correlation coefficient of 0.905. This regression equation could be in turn used to calibrate the calculated density of the *cyclo*-N_5_^−^ energetic salts accurately. This work provides a favorable way to explore the energetic salts with excellent performance based on *cyclo*-N_5_^−^.

## 1. Introduction

Energetic materials are a class of compounds that can release a large amount of energy after decomposition. They have long been used as important components of explosives, propellants, and pyrotechnics. The superior energy density makes them important energy sources in launch, propulsion, and detonation. Hence, they are used widely in strategic, tactical conventional weapon systems and arms and equipment. For the traditional CHON explosives, their energy source mainly depends on the intense redox reaction between the oxidant component and the combustible skeleton component. However, the energy level is limited by the oxygen balance. The oxygen balance directly affects the detonation effect of energetic materials. The ideal state for an explosion of energetic materials is zero oxygen equilibrium. Hence, if a certain explosive can achieve zero oxygen balance, it may release all the explosive energy. However, zero oxygen balance generally deteriorates the safety of the explosive system [1,2,3,4]. Moreover, theoretical and experimental studies show that the limiting density of traditional energetic materials is not greater than 2.20 g/cm^3^; as such, the energy level of traditional energetic materials is approaching this limit. Hence, designing new energetic compounds with excellent oxygen balance and high density to meet the energy requirements of energetic materials for the development of weapons is an important task. High-nitrogen energetic salts were designed and synthesized to meet these objectives. Compared with traditional energetic compounds, there is strong electrostatic interaction between the anions and cations in high-nitrogen energetic salts. Furthermore, there is no traditional energy functional group such as nitro in their structure. They are composed mainly of high-energy bonds of N–N and N=N, making it easier to form hydrogen bonds with H. These characteristics can improve the stability and density. On the basis of the relevant empirical formulas, the detonation performance of energetic materials is closely related to the density of the material, and the higher the crystal density, the better the detonation performance. Furthermore, the high energy of high-nitrogen energetic salts comes from the high formation enthalpy of N–N and N=N, releasing large amounts of nitrogen upon decomposition. Compared with the traditional energetic materials, the way of energy release in high-nitrogen energetic salts has changed fundamentally. These characteristics not only break the limitation of oxygen balance on detonation properties of energetic materials, but also guarantee that the released products are environmentally friendly. So, high-nitrogen energetic salts have broad application in the fields of new high-energy insensitive explosives, low-characteristic signal propellants and low-residue gunpowder. High-nitrogen energetic salts have become an excellent alternative to traditional energetic materials due to their energy properties and stabilities. The synthesis of high-energy total nitrogen compounds has pushed this field to the climax.

The synthesis of pentazolate anion is undoubtedly a hotspot in the field of high-nitrogen ion salts. In 2017, Zhang et al. first synthesized a salt (N_5_)_6_(H_3_O)_3_(NH_4_)_4_Cl (PHAC) [5] that could be stable at room temperature and give the stable crystal structure of the *cyclo*-N_5_^−^ salt. The successful synthesis of this compound is of great significance to the development of energetic materials. Subsequently, a series of *cyclo*-N_5_^−^ salts such as (NH_3_OH)^+^N_5_^−^, (NH_4_)^+^N_5_^−^ and [C(NH_2_)_3_]^+^N_5_^−^ [6,7] were synthesized. However, the research on the combination of *cyclo*-N_5_^−^ anion and nonmetal cations is still in the initial stage. In order to obtain *cyclo*-N_5_^−^ salts with ideal properties, it will take a lot of human and material resources.

Because of the maneuverability and dangerous of experimental research on energetic materials, it is difficult to study the new *cyclo*-N_5_^−^ salts in experiment. Hence, theoretical research is particularly important. In order to search for highly matched counterions of *cyclo*-N_5_^−^, we investigated 135 energetic salts by the DFT method [8] to see what factors make the cations and anions to be matchable (not including *cyclo*-N_5_^−^ salts). Furthermore, the thermal stabilities and crystal densities of the 17 nonmetallic energetic salts of *cyclo*-N_5_^−^ synthesized in experiment were also studied. We investigated the inherent law of what cations will be matchable with *cyclo*-N5^−^ and how to predict the densities of *cyclo*-N5^−^ salts precisely. This work provides theoretical guidance for theoretical design and experimental synthesis of *cyclo*-N_5_^−^ salts.

## 2. Results and Discussion

### 2.1. Thermal Stability

**Figure 1:**
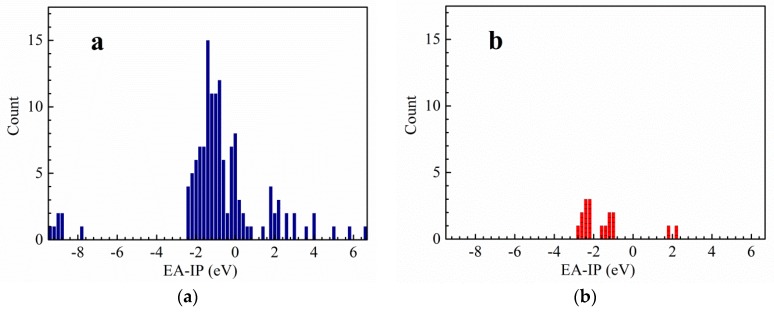
The difference between vertical electron affinity (*VEA*) of the cation and vertical ionization potential (*VIP*) of the anion for common energetic salts (**a**) and *cyclo*-N_5_^−^ salts (**b**) at DFT-B3LYP/6-311++G(d,p) level.

**Figure 2:**
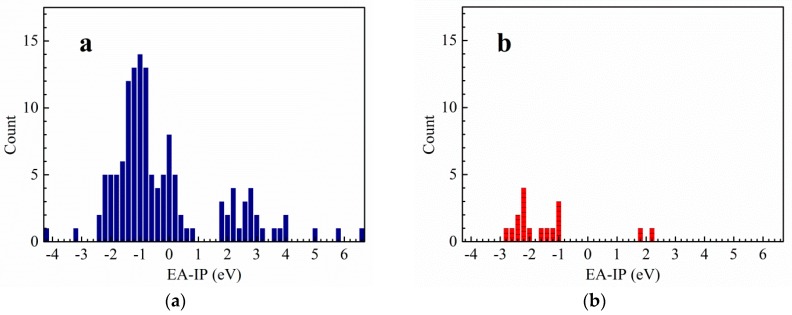
The difference between *VEA* of the cation and *VIP* of the anion for common energetic salts (**a**) and *cyclo*-N_5_^−^ salts (**b**) at DFT-B3LYP/aug-cc-pVTZ level.

**Figure 3:**
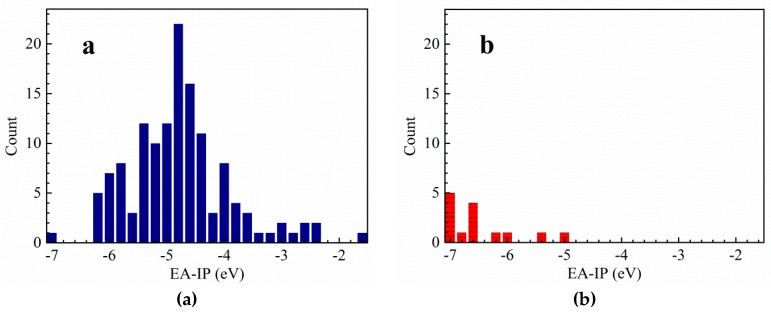
The difference between *VEA* of the cation and *VIP* of the anion for common energetic salts (**a**) and *cyclo*-N_5_^−^ salts (**b**) at DFT-B3LYP/6-311++G(d,p) level in water.

**Figure 4:**
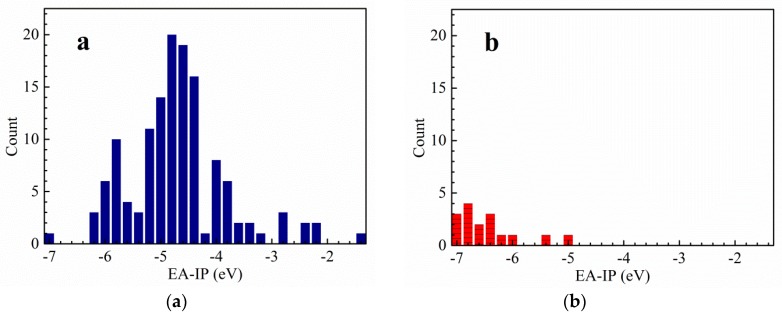
The difference between *VEA* of the cation and *VIP* of the anion for common energetic salts (**a**) and *cyclo*-N_5_^−^ salts (**b**) at DFT-B3LYP/aug-cc-pVTZ level in water.

**Figure 5:**
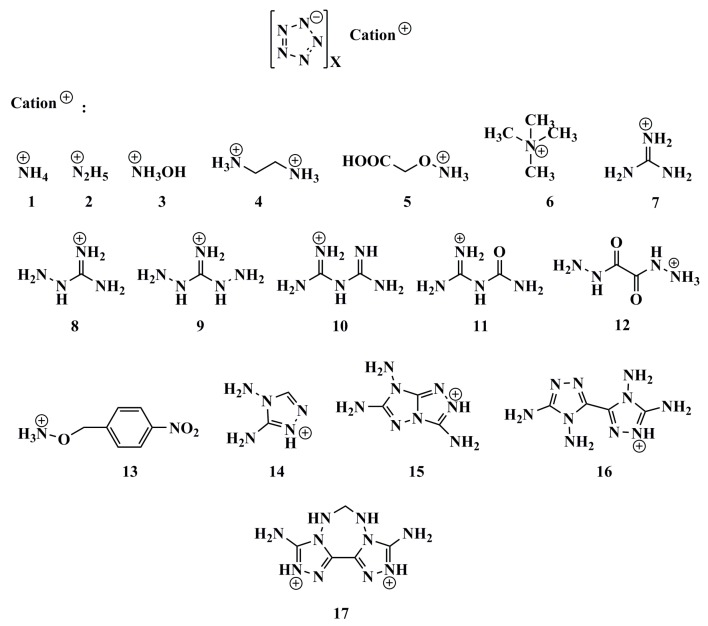
Structures of a series of cations in *cyclo*-N_5_^−^ salts.

In the light of the first approximation principle, the electron affinity of the cation and the ionization potential of the anion can directly affect the thermal stability of the salt [9]. When the two values are close to each other, the salt has good stability. Theoretically, for the *cyclo*-N_5_^−^ salt, the magnitude of vertical electron affinity (*VEA*) of an ideal cation should be similar to that of the vertical ionization potential (*VIP*) of *cyclo*-N_5_^−^ anion. In order to find cations that match well with *cyclo*-N_5_^−^ anion, the *VEA* of the cations and *VIP* of 135 energetic salts [10,11,12,13,14,15,16,17,18,19] and 17 synthetic *cyclo*-N_5_^−^ anion salts [6,20,21,22,23] were calculated by DFT method at B3LYP/6-311++G(d,p) and B3LYP/aug-cc-pVTZ levels (Table S1). On this basis, polarizable continuum model (PCM) solvation model was used to calculate the *VEA* of the cations and the *VIP* of the anions in water. In fact, both the vertical and adiabatic values of EA and IP should be compared with the experimental ones before an appropriate routine is selected. However, the *cyclo*-N5^−^ structure cannot converge to an optimized one at the DFT-B3LYP/6-311++G(d,p) level and is seriously deformed from the coplanar five numbered ring. On this condition, we used the *VEA* and *VIP*. The variations of the differences between *VEA* of cations and *VIP* of anions are statistically analyzed at four computational levels. Thereafter, the range of the differences between the *VEA* of cations and *VIP* of *cyclo*-N_5_^−^ anion are obtained by the above analysis (Figure 1,Figure 2,Figure 3,Figure 4). The structures of a series of cations of *cyclo*-N_5_^−^ salts are shown in Figure 5.

As can be seen from Figure 1, the difference between the *VEA* of the cations and *VIP* of the anions of 135 energetic salts is centralized and distributed in the range of −2.4 to 0.8 eV at the B3LYP/6-311++G(d,p) level of theory, meanwhile the corresponding values of *cyclo*-N_5_^−^ salts are in the range of −2.8 to −1.0 eV. The results at the B3LYP/aug-cc-pVTZ are basically similar except that there is an additional distribution in range of 1.8 to 4.0 eV. As shown in Figure 3, the corresponding values for the 135 salts range from −6.2 to −2.4 eV using PCM solvation model at the B3LYP/6-311++G(d,p) level, and those of *cyclo*-N_5_^−^ salts range from −7.2 to −6.0 eV. The differences between the *VEA* and *VIP* of the 135 salts focus on −6.2 to −3.2 eV, while the differences of the *cyclo*-N_5_^−^ salts are −7.2 to −6.0 eV using PCM solvation model at the B3LYP/aug-cc-pVTZ level.

Although the differences of the *VEA* and *VIP* for the 135 energetic salts are in a wide range, most values are centralized in a certain narrow range. The results provide references for screening out energetic salts in the future. As can be seen from Figure 1 and Figure 2, the differences between *VEA* and *VIP* of the 135 energetic salts are overlapped with those of *cyclo*-N_5_^−^ salts when the solvent effects are not involved in the calculations. The differences between *VEA* and *VIP* of the 135 salts are hardly overlapped with those of *cyclo*-N_5_^−^ salts as a whole (Figure 3 and Figure 4), indicating that the solvent effects on the 135 ordinary energetic salts are different from those on *cyclo*-N_5_^−^ salts since the Mulliken charges on *cyclo*-N_5_^−^ (−0.20 a.u. on all nitrogen atoms) are evenly distributed over its five-numbered ring.

Since the *VEA*–*VIP* differences of the 135 ordinary energetic salts are not matched well with those *cyclo*-N_5_^−^ salts when solvent effect of water is included, we suggested to use the *VEA*–*VIP* results at B3LYP/6-311++G(d,p) level without considering the solvent effect for screening out new counterions of *cyclo*-N_5_^−^. When the absolute values of *VEA*–*VIP* differences are less than 3 eV, especially *VEA*–*VIP* differences near −2 eV at the B3LYP/6-311++G(d,p) level, there is a high probability of *cyclo*-N_5_^−^ forming a stable salt with its counterion. It should be noted that hardly any salts exist with a *VEA*–*VIP* difference near zero, although the magnitudes of *VEA* and *VIP* for a stable salt are expected to be equal theoretically.

### 2.2. Density

Generally speaking, detonation velocity (*D*) and detonation pressure (*P*) are the most important indexes to measure the detonation performance of energetic materials. According to the Kamlet–Jacobs equation, the density of the explosive is directly related to the detonation velocity and the detonation pressure [24]. This means that the density of crystal is the most significant physical parameter for its detonation properties. In this section, the densities for a series of *cyclo*-pentazolate (*cyclo*-N_5_^−^) salts are calculated and the relationship between the calculated densities and experimental values is established. Table 1 shows the densities of the *cyclo*-N_5_^−^ salts. In general, *cyclo*-N_5_^−^ salts have better detonation performance than traditional energetic compounds. In this paper, *cyclo*-N_5_^−^ salt with a density less than 1.5 g/cm^3^ (salt **4:** *ρ*_Expt_ = 1.462 g/cm^3^, *D* = 6.896 km/s, *P* = 17.5 GPa) [21] is considered as low density; *cyclo*-N_5_^−^ salt with a density greater than 1.6 g/cm^3^ (salt **3:** *ρ*_Expt_ = 1.636 g/cm^3^, *D* = 9.005 km/s, *P* = 32.7 GPa) [21] is considered as high density; and *cyclo*-N_5_^−^ salt with a density greater than 1.6 g/cm^3^ can be used as a good high-energy material.

It can be seen from Figure 5 and Table 1 that the densities of *cyclo*-N_5_^−^ salts are small when the cations are made up of chain structures without oxygen atoms. According to Kamlet–Jacobs equation, these cations are bad candidates for high-performance salts with *cyclo*-N_5_^−^. Judged from **7** to **9**, the density of the crystal decreases gradually with the lengthening of the nitrogen chain if the chain cation contains only one C=NH_2_^+^. In other words, increasing the length of the nitrogen chain of cation is not beneficial to improving the detonation performance of *cyclo*-N_5_^−^ salt anion. For the energetic salts **14**, **15**, **17** with azole cations, as the number of azole rings increases, the density of the compound increases. This means that more azole rings are favorable for good detonation performance when they combine with *cyclo*-N_5_^−^.

As can be seen in Table 1, the density values obtained by using *M/V* are smaller than those from Equation (3), and the values obtained from Equation (3) are smaller than the experimental values. In other words, there is a large difference between the value of density obtained by *M/V* and the experimental density. Therefore, it is not suitable for *M/V* to predict the density of the salt.

**Figure 6:**
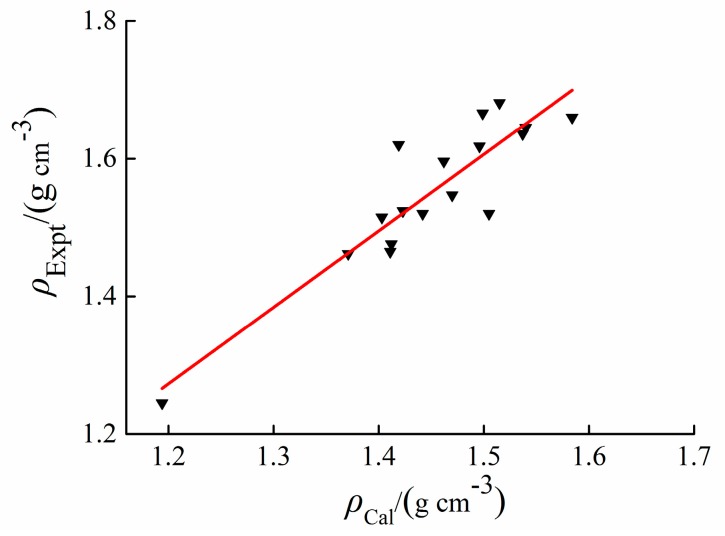
Comparison of *ρ*_Cal_ and *ρ*_Expt_ for *cyclo*-N_5_^−^ salts.

The theoretical densities (*ρ*_cal_) of *cyclo*-N_5_^−^ salts are close to the reference values (*ρ*_Expt_). A linear regression relationship between *ρ*_cal_ and *ρ*_Expt_ is established by SPSS package, as shown in Figure 6. The regression equation is: *ρ*_Expt_ = 1.111*ρ*_cal_ − 0.06067. The correlation coefficient of the regression equation is 0.905. The density from Equation (3) is further calibrated by the regression equation. For salts **1** to **17**, the cations of **1** and **2** contain only carbon and hydrogen atoms, which results in a large difference between the corrected value and the experimental value of density. The mean absolute error and the maximum absolute error (Δ*ρ*) are 0.034 and 0.104 g/cm^3^, respectively. Therefore, this method can be used to calibrate the density of *cyclo*-N_5_^−^ salts further.

## 3. Computational Methods

### 3.1. Vertical Electron Affinity and Vertical Ionization Potential

The structures of energetic salts (Tables S2–S154) were optimized by density functional theory (DFT) by B3LYP method with 6-311++G(d,p) and aug-cc-pVTZ basis sets [25,26]. On this basis, PCM solvation model was used to optimize the geometries of salts in aqueous phase since the *cyclo*-N_5_^−^ salts and most other energetic salts are crystalized in water. All of the quantum chemical calculations were completed by Gaussian 09 program package (G09) [27].

Vertical electron affinity and vertical ionization potential represent the attraction of cation to an electron and the repulsion of anion to an electron, respectively [28]. The *VEA* can be calculated in the following equation:

*VEA* = *E*_0_ (*cation*) − *E*_0_ (*cation* + *e*) (1)

where *E*_0_ (*cation*) represents the total energy of the cation at 0 K; *E*_0_ (*cation* + *e*) is the single-point energy of the cation after gaining an electron.

Similarly, *VIP* is calculated as follows:

*VIP* = *E*_0_ (*anion* − *e*) − *E*_0_ (*anion*) (2)

where *E*_0_ (*anion* *−* e) is the single-point energy of the anion after losing an electron.

### 3.2. Density

The density of the energetic salt is a significant parameter to predict its energetic performance and can directly affect its detonation performance. Several new methods have been developed to predict the crystal density precisely [29,30,31,32]. Compared with the traditional calculation method (*M/V*), these methods take into account the intermolecular interactions within the crystal. The crystal density of the energetic salt can be calculated by the following equation [33]:

*ρ* (g/cm^3^) = *α*(*M*/*V*) + *β*(*V*_S_^+^/*A*_S_^+^) + *γ*(*V*_S_^−^/*A*_S_^−^) + *δ* (3)

where *M* is the molecular mass of the chemical formula, and *V* is the total volume of the compound. *A*_S_^+^ is the portion of a cation’s surface that has a positive electrostatic potential, and *V*_S_^+^ is the average value of that potential; *A*_S_*^−^* and *V*_S_*^−^* are the analogous quantities for an anion. We can get these surface electrostatic potentials through the Multiwfn program [34].

In addition, the volume of compound whose molecular formula is *M*_p_*X*_q_ can be calculated by the following formula:

*V* = *pV*_M_^+^ + *qV*_X_^−^ (4)

where *V*_M_^+^ is expressed as the volume of the cation, and *V*_X_^−^ is expressed as the volume of the anion. In general, the DFT method is used to calculate the volumes for the various ions. The ionic volume was obtained from averaging 100 single-point molar volume calculations per ion molecule using a Monte Carlo integration scheme that is implemented in G09. The volume of the energetic salt is calculated by Formula (4).

## 4. Conclusions

The *VEA* of the cation and the *VIP* of the anion of 152 energetic salts were calculated at the B3LYP/6-311++G(d,p) and B3LYP/aug-cc-pVTZ levels with and without PCM solvent effect in water. The results indicate that under the condition of PCM/water, the difference between *VEA* and *VIP* of the synthesized *cyclo*-N5^−^ salts deviates from the centralized value ranges of 135 energetic salts. In other words, the difference between *VEA* and *VIP* of ordinary energetic salts is not applicable for the design of *cyclo*-N5^−^ salts on this condition. However, when PCM/water solvent effect is excluded, the difference between *VEA* and *VIP* of the *cyclo*-N5^−^ salts is centralized in the range of −2.8 to −1.0 eV at the B3LYP/6-311++G(d,p) and B3LYP/aug-cc-pVTZ levels and is basically located in the corresponding value range of the 135 energetic salts. Meanwhile, the results also indicate that the calculation results at the B3LYP/6-311++G(d,p) level are suitable for the theoretical prediction of *VEA* and *VIP* for *cyclo*-N_5_^−^ salts.

Compared with the cations of furazan and triazole, the nonaromatic cation without oxygen atom is not a good candidate to form energetic salt with *cyclo*-N_5_^−^ due to the low density. When there is only one C=NH_2_^+^, the density of the salt decreases as the nitrogen chain length increases. The densities of *cyclo*-N_5_^−^ salts were predicted at the B3LYP/6-311++G(d,p) level. The regression equation (*ρ*_Expt_ = 1.111*ρ*_cal_ − 0.06067) with a correlation coefficient of 0.905 was established between the predicted and the experimental densities. This equation can be used to search for new *cyclo*-N_5_^−^ energetic salts with excellent detonation performance.

## Figures and Tables

**Table 1:** Volumes (cm^3^/mol) and densities (g/cm^3^) of the salts with *cyclo*-N_5_^−.^

Salts	*M*	*V*	*A*_S_^+^	*V*_S_^+^	*A*_S_^−^	*V*_S_^−^	*M/V*	*ρ*_Cal_	*ρ*_Expt_	*ρ*_Calib_	Δ*ρ*
**1**	88	66.76	48.38	170.38	96.19	−117.64	1.318	1.505	1.520	1.611	0.091
**2**	103	79.03	67.94	145.35	96.19	−117.64	1.303	1.419	1.620	1.516	−0.104
**3**	104	74.03	62.49	151.61	96.19	−117.64	1.405	1.537	1.636	1.647	0.011
**4**	256	203.05	111.01	229.67	96.19	−117.64	1.261	1.371	1.462	1.463	0.001
**5**	162	112.28	121.94	110.91	96.19	−117.64	1.443	1.499	1.666	1.605	−0.061
**6**	144	125.18	130.58	107.72	96.19	−117.64	1.150	1.194	1.245	1.266	0.021
**7**	130	97.54	97.39	121.87	96.19	−117.64	1.333	1.403	1.515	1.498	−0.017
**8**	145	107.09	113.24	113.51	96.19	−117.64	1.354	1.412	1.476	1.508	0.032
**9**	160	117.52	128.88	106.75	96.19	−117.64	1.361	1.411	1.465	1.507	0.042
**10**	172	124.88	138.34	102.26	96.19	−117.64	1.377	1.423	1.524	1.520	−0.004
**11**	173	122.36	134.14	104.48	96.19	−117.64	1.414	1.462	1.596	1.564	−0.032
**12**	189	128.54	145.41	98.67	96.19	−117.64	1.470	1.515	1.681	1.622	−0.059
**13**	239	166.09	198.23	84.87	96.19	−117.64	1.439	1.470	1.547	1.573	0.026
**14**	170	117.72	128.66	107.92	96.19	−117.64	1.444	1.496	1.618	1.601	−0.017
**15**	225	149.78	173.82	91.67	96.19	−117.64	1.502	1.540	1.645	1.650	0.005
**16**	321	227.11	209.57	82.82	96.19	−117.64	1.413	1.442	1.520	1.541	0.021
**17**	350	228.36	213.5	167.61	96.19	−117.64	1.533	1.584	1.660	1.699	0.039

*M*—molecular mass, *V*—molecular volume, *A*_S_^+^—the portion of a cation’s surface that has a positive electrostatic potential, *V*_S_^+^—the average value of that potential; *A*_S_*^−^* and *V*_S_*^−^* are the analogous quantities for an anion. *ρ*_Cal_ represents the calculated density. *ρ*_Expt_ are experimental densities from [6,20,21,22,23]. *ρ*_Calib_ indicates the corrected density from the established equation of *ρ*_Calib_ = 1.111*ρ*_cal_ − 0.06067. Δ*ρ* shows the difference between the calibrated value and the measured value of density.

## Supplementary Materials

The following are available online at https://www.mdpi.com/1420-3049/25/8/1783/s1, Table S1: Vertical electron affinity of cations and vertical ionization potential of anions, Table S2–S154: Salt 2–154 Cartesian coordinates.
