# Supplementary material for: Theoretical Investigation of Energetic Salts with Pentazolate Anion
Source: Molecules. 2020 Apr 13;25(8):1783. doi: 10.3390/molecules25081783 (PMC7221799; doi:10.3390/molecules25081783)
Supplement: Supplementary file 1 [file molecules-25-01783-s001.pdf]

Supporting Information

Theoretical investigation of energetic salts with pentazolate anion

Hao-Ran Wang, Chong Zhang, Bing-Cheng Hu*, Xue-Hai Ju*

School of Chemical Engineering, Nanjing University of Science and Technology, Nanjing 210094, P. R. China.

Table S1 Ionization potential of cations and electron affinity of anions

salts	cation	EA(cation) B3LYP/6-311++G(d,p)	EA(cation) B3LYP/aug-cc-pVTZ	anion	IP(anion) B3LYP/6-311++G(d,p)	IP(anion) B3LYP/aug-cc-pVTZ
**1**	$NH_4^{\oplus}$	4.74	4.74	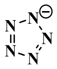	5.72	5.60
**2**	$N_2H_5^{\oplus}$	4.39	4.38	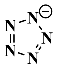	5.72	5.60
**3**	$NH_3OH^{\oplus}$	4.71	4.68	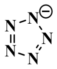	5.72	5.60
**4**	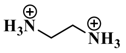	8.00	7.98	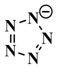	5.72	5.60
**5**	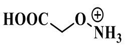	4.73	4.66	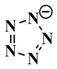	5.72	5.60
**6**	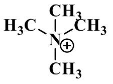	3.13	3.14	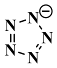	5.72	5.60
**7**	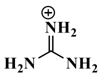	3.61	3.63	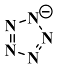	5.72	5.60
**8**	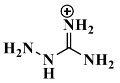	3.49	3.49	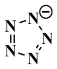	5.72	5.60
**9**	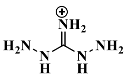	3.44	3.43	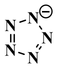	5.72	5.60
**10**	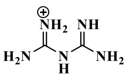	3.39	3.39	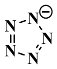	5.72	5.60
**11**	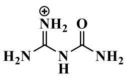	3.55	3.54	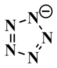	5.72	5.60
**12**	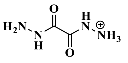	4.15	4.12	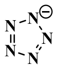	5.72	5.60

**13**	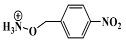	4.55	4.52	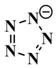	5.72	5.60
**14**	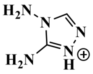	3.52	3.52	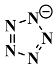	5.72	5.60
**15**	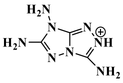	2.98	2.99	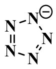	5.72	5.60
**16**	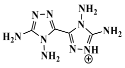	3.25	3.23	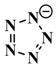	5.72	5.60
**17**	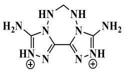	7.60	7.60	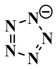	5.72	5.60
**18**	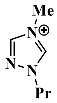	3.71	3.70	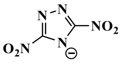	6.11	5.98
**19**	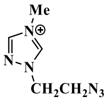	4.00	3.98	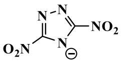	6.11	5.98
**20**	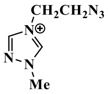	3.97	3.94	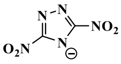	6.11	5.98
**21**	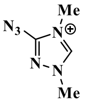	4.14	4.09	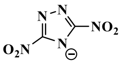	6.11	5.98
**22**	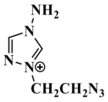	3.85	3.83	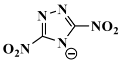	6.11	5.98
**23**	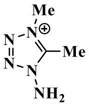	3.85	3.83	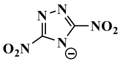	6.11	5.98
**24**	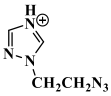	4.12	1.86	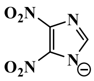	4.98	4.88
**25**	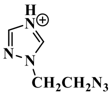	4.12	1.86	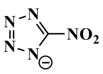	5.99	5.86
**26**	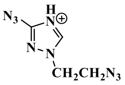	4.37	4.29	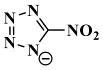	5.99	5.86
**27**	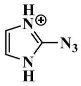	4.33	4.26	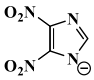	4.98	4.88
**28**	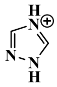	4.28	4.25	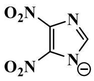	4.98	4.88

**29**	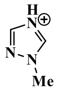	4.00	3.97	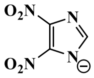	4.98	4.88
**30**	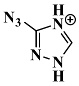	4.55	4.47	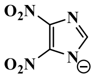	4.98	4.88
**31**	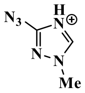	4.28	4.22	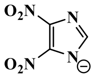	4.98	4.88
**32**	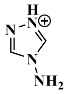	3.94	3.92	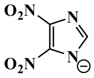	4.98	4.88
**33**	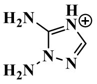	3.32	3.33	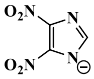	4.98	4.88
**34**	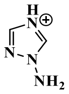	3.83	4.14	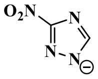	5.05	4.96
**35**	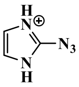	4.33	4.26	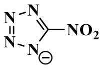	5.99	5.86
**36**	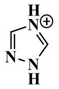	4.28	4.25	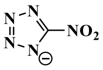	5.99	5.86
**37**	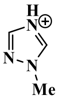	4.00	3.97	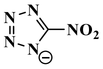	5.99	5.86
**38**	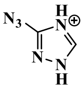	4.55	4.47	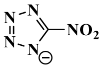	5.99	5.86
**39**	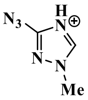	4.28	4.22	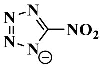	5.99	5.86
**40**	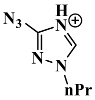	4.11	4.04	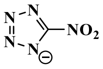	5.99	5.86
**41**	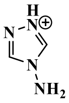	3.94	3.92	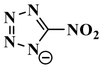	5.99	5.86
**42**	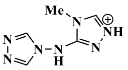	4.18	4.14	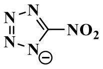	5.99	5.86
**43**	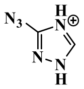	4.55	4.47	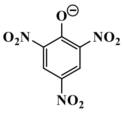	5.23	5.12

**44**	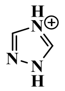	4.28	4.25	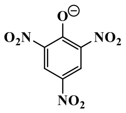	5.23	5.12
**45**	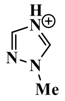	4.00	3.97	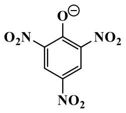	5.23	5.12
**46**	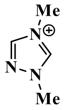	3.88	3.87	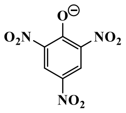	5.23	5.12
**47**	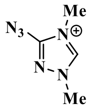	4.14	4.09	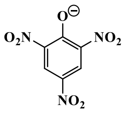	5.23	5.12
**48**	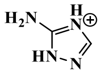	3.61	3.61	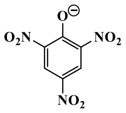	5.23	5.12
**49**	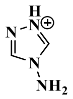	3.94	3.92	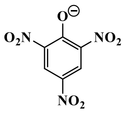	5.23	5.12
**50**	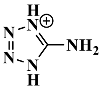	3.99	3.97	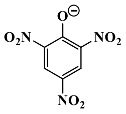	5.23	5.12
**51**	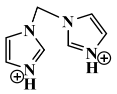	7.54	7.59	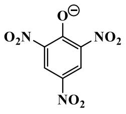	5.23	5.12
**52**	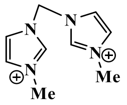	7.10	7.11	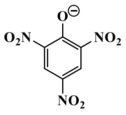	5.23	5.12
**53**	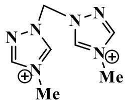	7.56	7.52	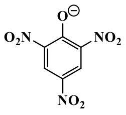	5.23	5.12
**54**	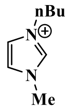	3.38	3.38	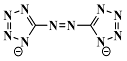	12.75	1.05
**55**	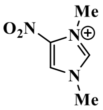	5.02	4.98	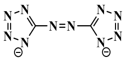	12.75	1.05
**56**	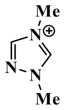	3.88	3.87	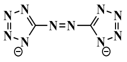	12.75	1.05
**57**	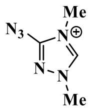	4.14	4.09	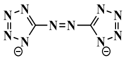	12.75	1.05

**58**	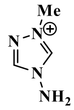	3.72	3.71	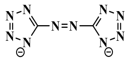	12.75	1.05
**59**	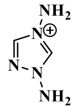	4.04	4.01	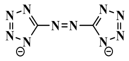	12.75	1.05
**60**	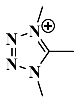	3.93	3.88	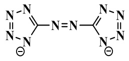	12.75	1.05
**61**	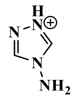	3.94	3.92	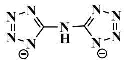	-0.19	-0.27
**62**	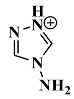	3.94	3.92	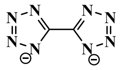	0.33	0.23
**63**	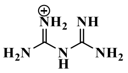	3.39	3.39	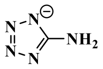	3.48	3.41
**64**	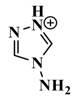	3.94	3.92	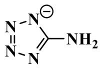	3.48	3.41
**65**	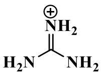	3.61	3.63	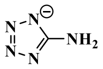	3.48	3.41
**66**	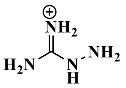	3.49	3.49	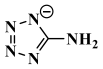	3.48	3.41
**67**	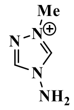	3.72	3.71	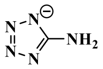	3.48	3.41
**68**	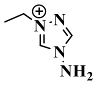	3.59	3.58	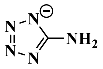	3.48	3.41
**69**	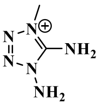	3.61	3.61	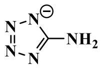	3.48	3.41
**70**	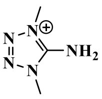	3.57	3.56	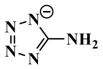	3.48	3.41
**71**	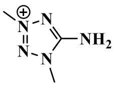	4.24	4.20	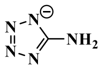	3.48	3.41
**72**	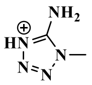	3.79	3.78	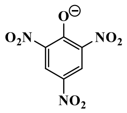	5.23	5.12
**73**	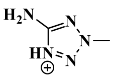	4.45	4.41	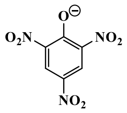	5.23	5.12

**74**	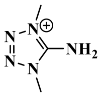	3.57	3.56	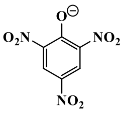	5.23	5.12
**75**	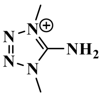	3.57	3.56	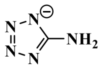	3.48	3.41
**76**	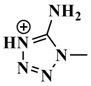	3.79	3.78	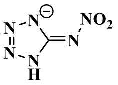	4.55	4.46
**77**	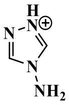	3.94	3.92	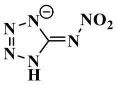	4.55	4.46
**78**	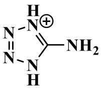	3.99	3.97	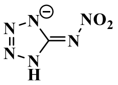	4.55	4.46
**79**	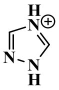	4.28	4.25	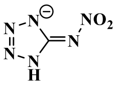	4.55	4.46
**80**	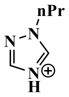	3.81	3.78	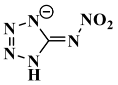	4.55	4.46
**81**	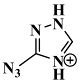	4.55	4.47	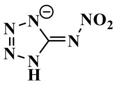	4.55	4.46
**82**	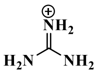	3.61	3.63	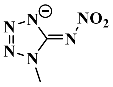	4.40	4.33
**83**	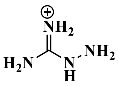	3.49	3.49	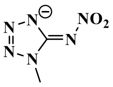	4.40	4.33
**84**	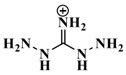	3.38	3.38	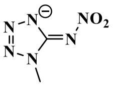	4.40	4.33
**85**	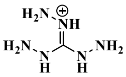	3.32	3.32	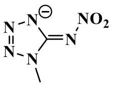	4.40	4.33
**86**	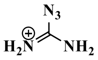	4.64	4.57	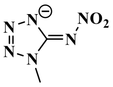	4.40	4.33
**87**	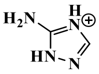	3.61	3.61	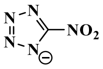	5.99	5.86
**88**	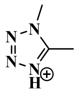	4.12	4.08	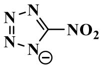	5.99	5.86
**89**	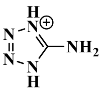	3.99	3.97	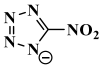	5.99	5.86
**90**	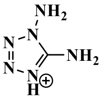	3.86	3.80	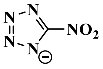	5.99	5.86

**91**	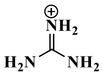	3.61	3.63	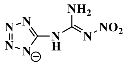	4.74	4.66
**92**	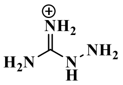	3.49	3.49	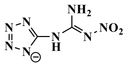	4.74	4.66
**93**	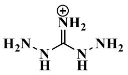	3.38	3.38	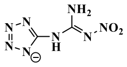	4.74	4.66
**94**	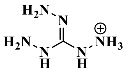	3.19	3.21	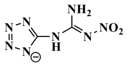	4.74	4.66
**95**	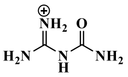	3.55	3.54	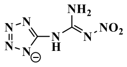	4.74	4.66
**96**	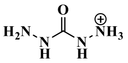	3.74	3.72	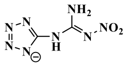	4.74	4.66
**97**	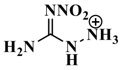	4.26	4.22	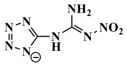	4.74	4.66
**98**	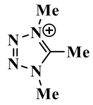	3.93	3.88	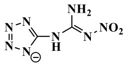	4.74	4.66
**99**	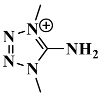	3.57	3.56	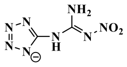	4.74	4.66
**100**	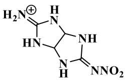	3.80	3.76	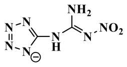	4.74	4.66
**101**	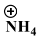	4.74	4.74	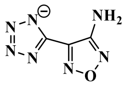	4.87	4.76
**102**	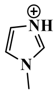	3.60	3.60	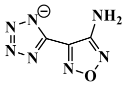	4.87	4.76
**103**	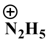	4.39	4.38	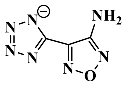	4.87	4.76
**104**	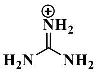	3.61	3.63	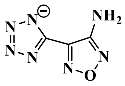	4.87	4.76
**105**	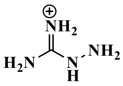	3.49	3.49	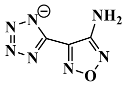	4.87	4.76
**106**	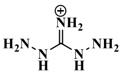	3.38	3.38	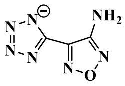	4.87	4.76
**107**	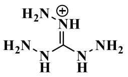	3.32	3.32	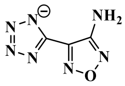	4.87	4.76
**108**	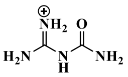	3.55	3.54	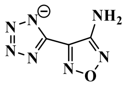	4.87	4.76
**109**	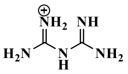	3.39	3.39	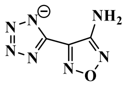	4.87	4.76
**110**	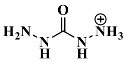	3.74	3.72	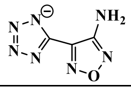	4.87	4.76

**111**	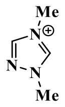	3.88	3.87	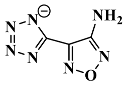	4.87	4.76
**112**	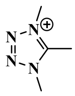	3.93	3.88	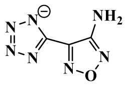	4.87	4.76
**113**	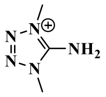	3.57	3.56	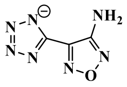	4.87	4.76
**114**	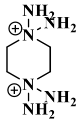	6.98	6.95	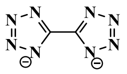	0.33	0.23
**115**	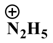	4.39	4.38	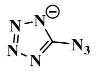	4.54	4.44
**116**	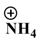	4.74	4.74	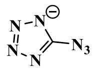	4.54	4.44
**117**	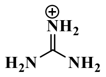	3.61	3.63	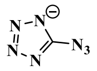	4.54	4.44
**118**	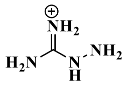	3.49	3.49	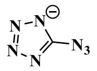	4.54	4.44
**119**	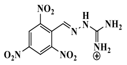	5.34	5.24	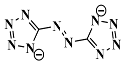	1.14	1.05
**120**	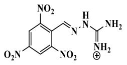	5.34	5.24	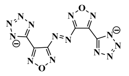	3.03	2.57
**121**	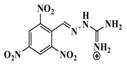	5.34	5.24	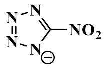	5.99	5.86
**122**	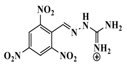	5.34	5.24	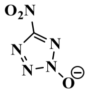	4.52	4.44
**123**	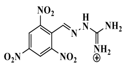	5.34	5.24	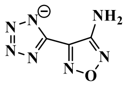	4.87	4.76
**124**	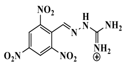	5.34	5.24	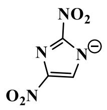	5.29	5.18
**125**	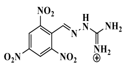	5.34	5.24	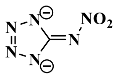	-0.53	-0.60
**126**	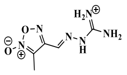	4.49	4.48	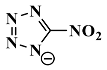	5.99	5.86
**127**	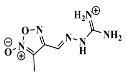	4.49	4.48	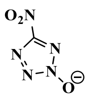	4.52	4.44
**128**	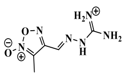	4.49	4.48	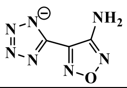	4.87	4.76

**129**	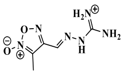	4.49	4.48	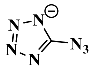	4.54	4.44
**130**	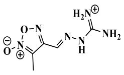	4.49	4.48	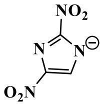	5.29	5.18
**131**	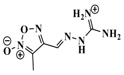	4.49	4.48	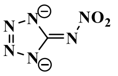	-0.53	-0.60
**132**	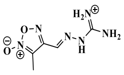	4.49	4.48	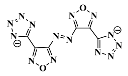	3.03	2.57
**133**	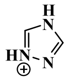	4.28	4.25	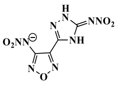	5.06	4.98
**134**	NH_4^{+}	4.74	4.74	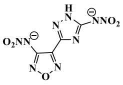	1.63	1.53
**135**	$N_2H_5^{+}$	4.39	4.38	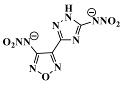	1.63	1.53
**136**	NH_3OH^{+}	4.71	4.68	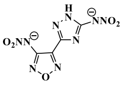	1.63	1.53
**137**	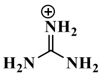	3.61	3.63	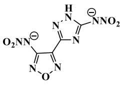	1.63	1.53
**138**	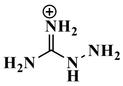	3.49	3.49	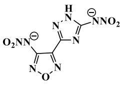	1.63	1.53
**139**	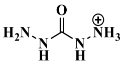	3.74	3.72	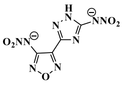	1.63	1.53
**140**	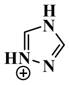	4.28	4.25	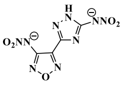	1.63	1.53
**141**	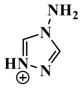	3.59	3.92	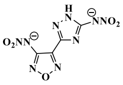	1.63	1.53
**142**	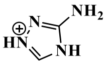	3.80	3.78	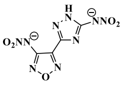	1.63	1.53
**143**	NH_4^{+}	4.74	4.74	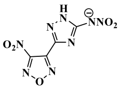	4.86	4.77
**144**	$N_2H_5^{+}$	4.39	4.38	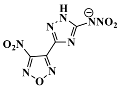	4.86	4.77
**145**	NH_3OH^{+}	4.71	4.68	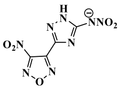	4.86	4.77
**146**	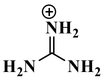	3.61	3.63	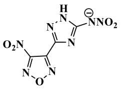	4.86	4.77

**147**	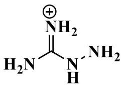	3.49	3.49	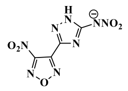	4.86	4.77
**148**	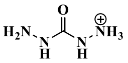	3.74	3.72	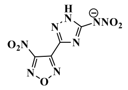	4.86	4.77
**149**	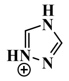	4.28	4.25	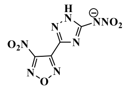	4.86	4.77
**150**	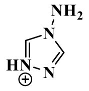	3.59	3.92	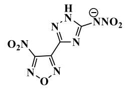	4.86	4.77
**151**	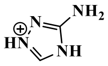	3.80	3.78	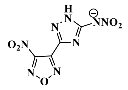	4.86	4.77
**152**	$N_2H_5^+$	4.39	4.38	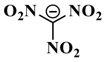	5.25	5.13

Ionization potential of cations and electron affinity of anions

**salts**	**cation**	**EA(cation)**		**anion**	**IP(anion)**	
		**B3LYP/6-311++G(d,p) PCM/water**	**B3LYP/aug-cc-pVTZ PCM/water**		**B3LYP/6-311++G(d,p) PCM/water**	**B3LYP/aug-cc-pVTZ PCM/water**
**1**	NH_4^+	1.64	1.64	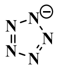	8.13	8.01
**2**	$N_2H_5^+$	1.54	1.54	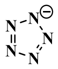	8.13	8.01
**3**	NH_3OH^+	1.72	1.71	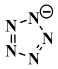	8.13	8.01
**4**	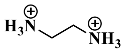	1.49	1.48	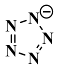	8.13	8.01
**5**	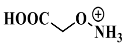	1.67	1.65	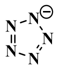	8.13	8.01
**6**	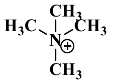	0.97	0.99	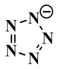	8.13	8.01
**7**	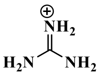	1.23	1.24	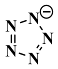	8.13	8.01
**8**	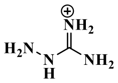	1.19	1.20	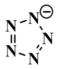	8.13	8.01
**9**	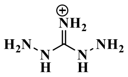	1.30	1.30	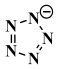	8.13	8.01
**10**	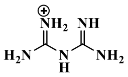	1.08	1.07	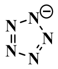	8.13	8.01

**11**	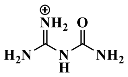	1.33	1.30	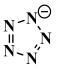	8.13	8.01
**12**	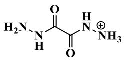	2.20	2.16	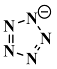	8.13	8.01
**13**	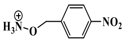	3.22	3.15	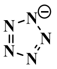	8.13	8.01
**14**	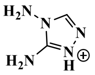	1.05	1.05	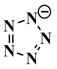	8.13	8.01
**15**	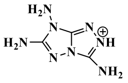	1.27	1.27	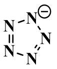	8.13	8.01
**16**	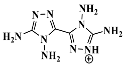	2.04	1.99	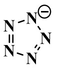	8.13	8.01
**17**	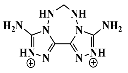	2.79	2.76	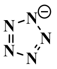	8.13	8.01
**18**	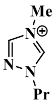	1.78	1.75	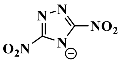	7.75	7.64
**19**	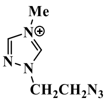	1.91	1.87	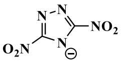	7.75	7.64
**20**	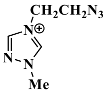	1.89	1.86	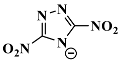	7.75	7.64
**21**	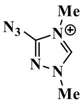	2.30	2.19	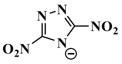	7.75	7.64
**22**	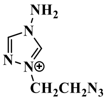	1.80	1.76	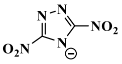	7.75	7.64
**23**	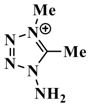	1.96	1.91	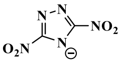	7.75	7.64
**24**	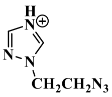	1.90	1.86	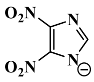	6.69	6.61
**25**	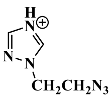	1.90	1.86	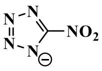	7.96	7.84
**26**	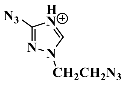	2.31	2.21	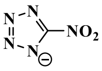	7.96	7.84
**27**	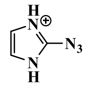	2.29	2.21	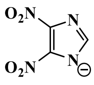	6.69	6.61

**28**	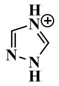	1.89	1.85	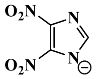	6.69	6.61
**29**	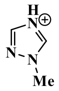	1.80	1.77	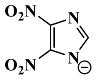	6.69	6.61
**30**	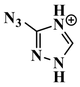	2.32	2.21	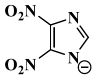	6.69	6.61
**31**	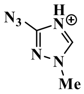	2.29	2.19	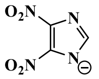	6.69	6.61
**32**	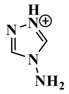	1.76	1.72	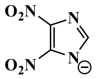	6.69	6.61
**33**	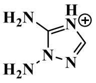	1.06	1.05	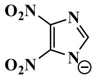	6.69	6.61
**34**	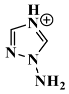	1.97	1.92	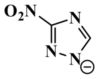	7.01	6.93
**35**	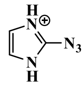	2.29	2.21	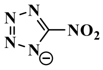	7.96	7.84
**36**	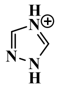	1.89	1.85	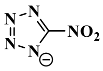	7.96	7.84
**37**	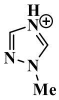	1.80	1.77	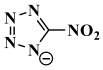	7.96	7.84
**38**	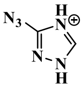	2.32	2.21	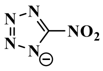	7.96	7.84
**39**	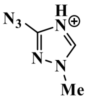	2.29	2.19	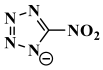	7.96	7.84
**40**	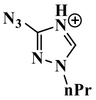	2.28	2.18	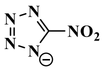	7.96	7.84
**41**	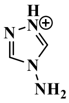	1.76	1.72	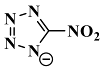	7.96	7.84
**42**	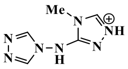	1.85	1.81	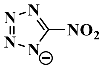	7.96	7.84

**43**	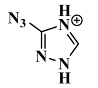	2.32	2.21	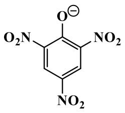	6.54	6.45
**44**	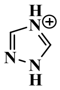	1.89	1.85	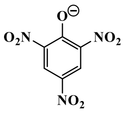	6.54	6.45
**45**	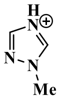	1.80	1.77	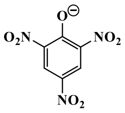	6.54	6.45
**46**	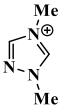	1.82	1.79	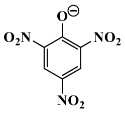	6.54	6.45
**47**	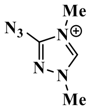	2.30	2.19	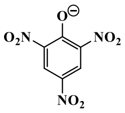	6.54	6.45
**48**	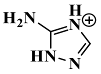	0.97	0.97	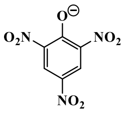	6.54	6.45
**49**	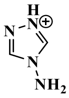	1.76	1.72	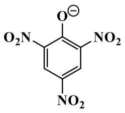	6.54	6.45
**50**	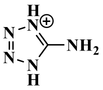	1.96	1.91	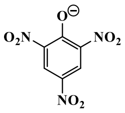	6.54	6.45
**51**	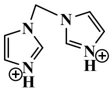	2.22	2.18	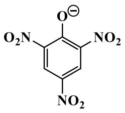	6.54	6.45
**52**	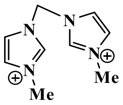	2.10	2.07	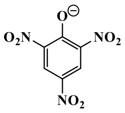	6.54	6.45
**53**	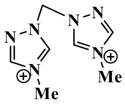	2.50	2.45	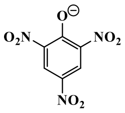	6.54	6.45
**54**	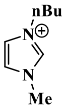	1.54	1.53	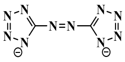	6.42	6.31
**55**	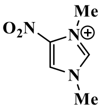	3.64	3.56	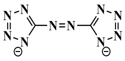	6.42	6.31
**56**	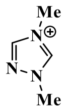	1.82	1.79	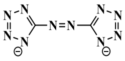	6.42	6.31

**57**	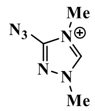	2.30	2.19	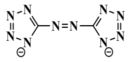	6.42	6.31
**58**	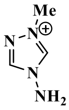	1.71	1.67	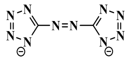	6.42	6.31
**59**	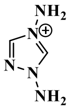	1.98	1.93	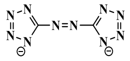	6.42	6.31
**60**	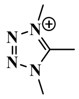	2.05	2.01	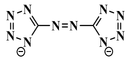	6.42	6.31
**61**	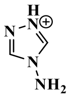	1.76	1.72	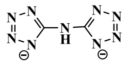	5.22	5.13
**62**	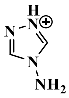	1.76	1.72	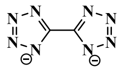	6.12	6.01
**63**	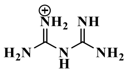	1.08	1.07	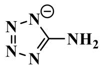	5.61	5.54
**64**	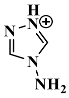	1.76	1.72	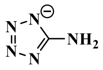	5.61	5.54
**65**	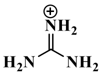	1.23	1.24	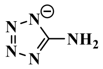	5.61	5.54
**66**	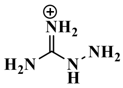	1.19	1.20	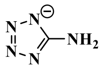	5.61	5.54
**67**	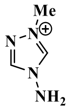	1.71	1.67	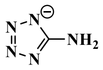	5.61	5.54
**68**	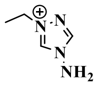	1.66	1.63	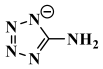	5.61	5.54
**69**	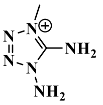	1.98	1.93	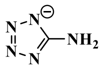	5.61	5.54
**70**	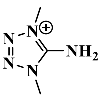	1.87	1.83	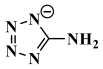	5.61	5.54
**71**	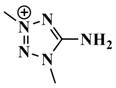	2.45	2.39	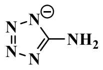	5.61	5.54
**72**	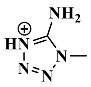	1.95	1.91	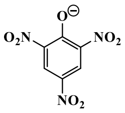	6.54	6.45

**73**	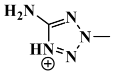	2.53	2.46	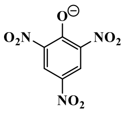	6.54	6.45
**74**	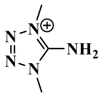	1.87	1.83	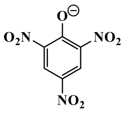	6.54	6.45
**75**	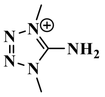	1.87	1.83	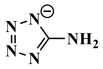	5.61	5.54
**76**	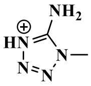	1.95	1.91	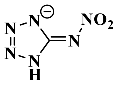	6.53	6.44
**77**	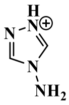	1.76	1.72	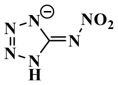	6.53	6.44
**78**	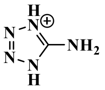	1.96	1.91	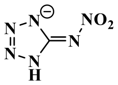	6.53	6.44
**79**	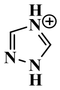	1.89	1.85	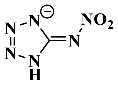	6.53	6.44
**80**	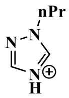	1.76	1.72	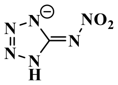	6.53	6.44
**81**	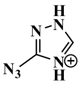	2.32	2.22	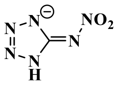	6.53	6.44
**82**	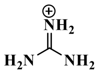	1.23	1.24	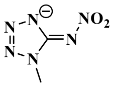	6.42	6.34
**83**	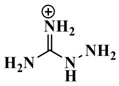	1.19	1.20	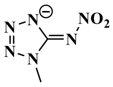	6.42	6.34
**84**	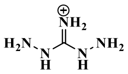	1.16	1.96	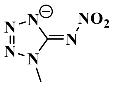	6.42	6.34
**85**	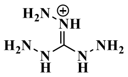	1.37	1.38	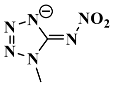	6.42	6.34
**86**	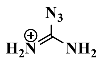	2.47	2.38	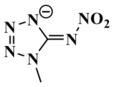	6.42	6.34
**87**	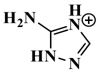	0.97	0.97	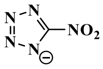	7.96	7.84
**88**	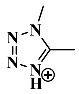	2.16	2.11	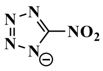	7.96	7.84
**89**	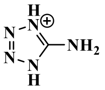	1.96	1.91	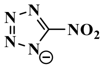	7.96	7.84

**90**	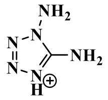	2.06	2.00	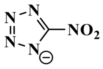	7.96	7.84
**91**	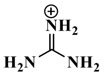	1.23	1.24	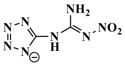	6.49	6.41
**92**	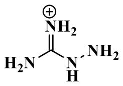	1.19	1.20	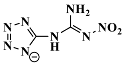	6.49	6.41
**93**	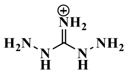	1.16	1.96	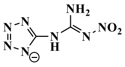	6.49	6.41
**94**	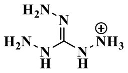	1.46	1.46	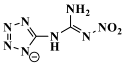	6.49	6.41
**95**	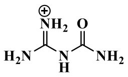	1.33	1.30	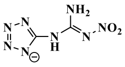	6.49	6.41
**96**	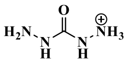	1.53	1.51	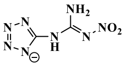	6.49	6.41
**97**	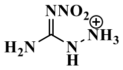	2.74	2.64	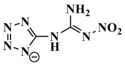	6.49	6.41
**98**	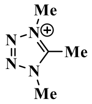	2.05	2.01	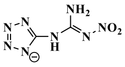	6.49	6.41
**99**	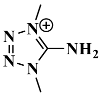	1.87	1.83	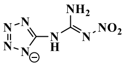	6.49	6.41
**100**	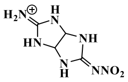	2.51	2.41	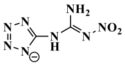	6.49	6.41
**101**	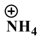	1.64	1.64	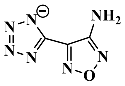	6.56	6.47
**102**	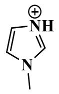	1.51	1.49	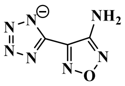	6.56	6.47
**103**	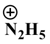	1.54	1.54	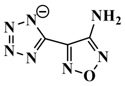	6.56	6.47
**104**	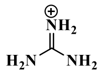	1.23	1.24	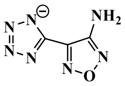	6.56	6.47
**105**	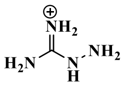	1.19	1.20	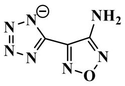	6.56	6.47
**106**	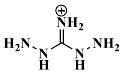	1.16	1.96	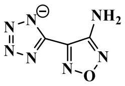	6.56	6.47
**107**	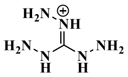	1.37	1.38	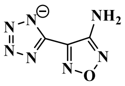	6.56	6.47
**108**	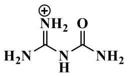	1.33	1.30	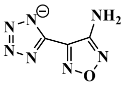	6.56	6.47
**109**	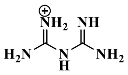	1.08	1.07	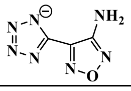	6.56	6.47

**110**	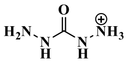	1.53	1.51	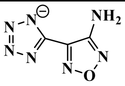	6.56	6.47
**111**	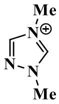	1.82	1.79	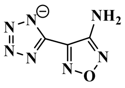	6.56	6.47
**112**	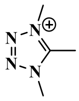	2.05	2.01	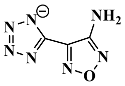	6.56	6.47
**113**	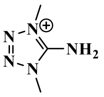	1.87	1.83	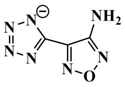	6.56	6.47
**114**	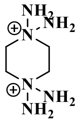	1.43	1.42	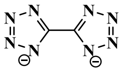	6.12	6.01
**115**	$N_2H_5^{+}$	1.54	1.54	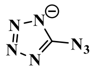	6.49	6.40
**116**	NH_4^{+}	1.64	1.64	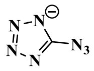	6.49	6.40
**117**	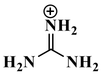	1.23	1.24	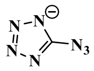	6.49	6.40
**118**	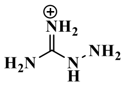	1.19	1.20	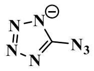	6.49	6.40
**119**	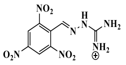	4.04	4.16	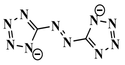	6.42	6.31
**120**	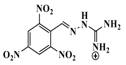	4.04	4.16	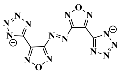	6.98	6.87
**121**	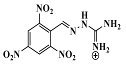	4.04	4.16	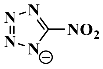	7.96	7.84
**122**	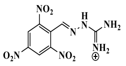	4.04	4.16	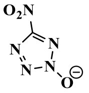	6.42	6.35
**123**	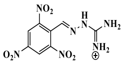	4.04	4.16	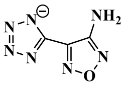	6.56	6.47
**124**	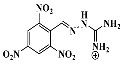	4.04	4.16	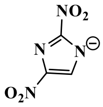	6.90	6.81
**125**	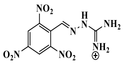	4.04	4.16	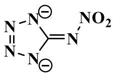	5.49	5.40
**126**	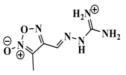	3.07	3.02	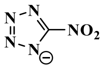	7.96	7.84
**127**	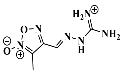	3.07	3.02	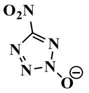	6.42	6.35

**128**	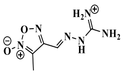	3.07	3.02	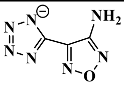	6.56	6.47
**129**	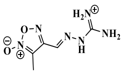	3.07	3.02	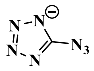	6.49	6.40
**130**	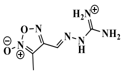	3.07	3.02	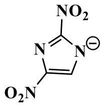	6.90	6.81
**131**	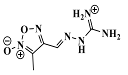	3.07	3.02	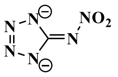	5.49	5.40
**132**	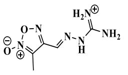	3.07	3.02	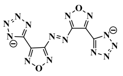	6.98	6.87
**133**	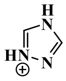	1.89	1.85	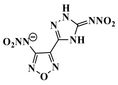	6.62	6.53
**134**	$\overset{\oplus}{N}H_4$	1.64	1.64	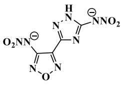	6.18	6.09
**135**	$\overset{\oplus}{N}_2H_5$	1.54	1.54	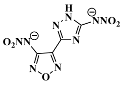	6.18	6.09
**136**	$\overset{\oplus}{N}H_3OH$	1.72	1.71	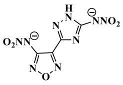	6.18	6.09
**137**	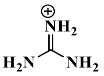	1.23	1.24	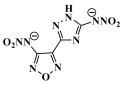	6.18	6.09
**138**	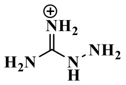	1.19	1.20	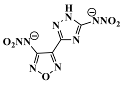	6.18	6.09
**139**	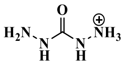	1.53	1.51	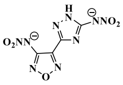	6.18	6.09
**140**	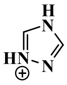	1.89	1.85	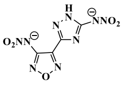	6.18	6.09
**141**	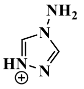	1.76	1.72	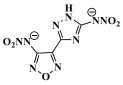	6.18	6.09
**142**	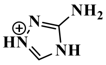	1.67	1.63	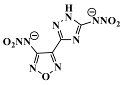	6.18	6.09
**143**	$\overset{\oplus}{N}H_4$	1.64	1.64	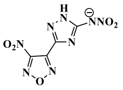	6.41	6.33
**144**	$\overset{\oplus}{N}_2H_5$	1.54	1.54	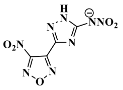	6.41	6.33
**145**	$\overset{\oplus}{N}H_3OH$	1.72	1.71	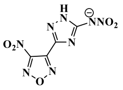	6.41	6.33
**146**	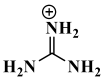	1.23	1.24	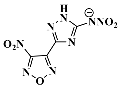	6.41	6.33

**147**	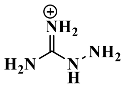	1.19	1.20	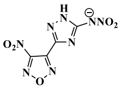	6.41	6.33
**148**	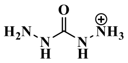	1.53	1.51	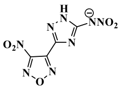	6.41	6.33
**149**	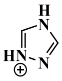	1.89	1.85	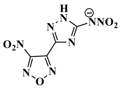	6.41	6.33
**150**	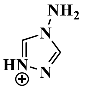	1.76	1.72	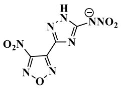	6.41	6.33
**151**	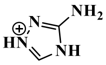	1.67	1.63	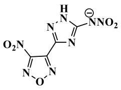	6.41	6.33
**152**	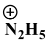	1.54	1.54	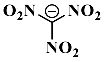	7.43	7.34

Table S2 Cartesian coordinates of salts

Salt 1

Cartesian coordinates

	Atom	X	Y	Z
Cation	N1	−0.00001200	0.00000000	0.00000000
	H2	0.09263200	0.83857300	−0.58434300
	H3	0.10779100	−0.83730900	−0.58355400
	H4	0.72571300	0.00690600	0.72562600
	H5	−0.92605200	−0.00816900	0.44227100
Anion	N1	1.09782300	−0.25964600	0.00008600
	N2	0.58607900	0.96336400	−0.00000300
	N3	−0.73534900	0.85500500	−0.00008100
	N4	−1.04076500	−0.43504700	0.00013400
	N5	0.09221200	−1.12367700	−0.00013600

SCF Done: E(B3LYP, cation) = −56.9203571 a.u.

SCF Done: E(B3LYP, anion) = −273.8228097 a.u.

Table S2: Salt 2

Cartesian coordinates

	Atom	X	Y	Z
Cation	N1	0.65541700	−0.00411800	0.00005100
	N2	−0.75045600	−0.00232700	−0.00003900
	H3	0.99718400	0.96411800	−0.00012100
	H4	1.05415400	−0.45557600	0.83700000
	H5	1.05425800	−0.45589200	−0.83667700
	H6	−1.21844400	−0.89213100	−0.00011000
	H7	−1.22187800	0.88459500	−0.00017400
Anion	N1	1.09782300	−0.25964600	0.00008600
	N2	0.58607900	0.96336400	−0.00000300
	N3	−0.73534900	0.85500500	−0.00008100
	N4	−1.04076500	−0.43504700	0.00013400
	N5	0.09221200	−1.12367700	−0.00013600

SCF Done: E(B3LYP, cation) = −112.2418137 a.u.

SCF Done: E(B3LYP, anion) = −273.8228097 a.u.

Table S3: Salt 3

Cartesian coordinates

	Atom	X	Y	Z
Cation	N1	0.62157632	0.00638917	0.00006179
	O2	−0.73539340	0.09712580	−0.00012371
	H3	1.01706940	0.92485812	−0.00007138
	H4	0.92261491	−0.48604256	0.81669314
	H5	0.92281651	−0.48636615	−0.81629998
	H6	−1.11506675	−0.78460439	0.00000414
Anion	N1	1.09782300	−0.25964600	0.00008600
	N2	0.58607900	0.96336400	−0.00000300
	N3	−0.73534900	0.85500500	−0.00008100
	N4	−1.04076500	−0.43504700	0.00013400
	N5	0.09221200	−1.12367700	−0.00013600

SCF Done: E(B3LYP, cation) = −132.0865145 a.u.

SCF Done: E(B3LYP, anion) = −273.8228097 a.u.

Table S4: Salt 4

Cartesian coordinates

	Atom	X	Y	Z
Cation	N1	−1.91505800	0.25420600	0.00009700
	C2	−0.59327600	−0.49095800	−0.00011400
	C3	0.59327600	0.49095800	0.00011400

	N4	1.91505800	−0.25420600	−0.00009700
	H5	−2.69714000	−0.41647900	−0.00005800
	H6	−2.04212000	0.84899600	0.83022300
	H7	−2.04215100	0.84941000	−0.82972700
	H8	−0.59832600	−1.12605500	−0.88826400
	H9	−0.59829300	−1.12650600	0.88771500
	H10	0.59832600	1.12605500	0.88826400
	H11	0.59829300	1.12650600	−0.88771500
	H12	2.69714000	0.41647900	0.00005800
	H13	2.04212000	−0.84899600	−0.83022300
	H14	2.04215100	−0.84941000	0.82972700
Anion	N1	1.09782300	−0.25964600	0.00008600
	N2	0.58607900	0.96336400	−0.00000300
	N3	−0.73534900	0.85500500	−0.00008100
	N4	−1.04076500	−0.43504700	0.00013400
	N5	0.09221200	−1.12367700	−0.00013600

SCF Done: E(B3LYP, cation) = −191.1523481 a.u.

SCF Done: E(B3LYP, anion) = −273.8228097 a.u.

Table S5: Salt 5

Cartesian coordinates

	Atom	X	Y	Z
Cation	C1	0.16382400	−0.67325800	0.00007200
	O2	1.15888500	0.40401800	−0.00000400
	N3	2.48364200	−0.08453600	0.00006000
	C4	−1.25714200	−0.07579200	−0.00001700
	O5	−2.16451800	−0.84597600	0.00008500
	O6	−1.40227400	1.24881900	−0.00019900
	H7	0.27556900	−1.28034500	0.90223800
	H8	0.27561500	−1.28051000	−0.90197700
	H9	3.08891000	0.74710800	−0.00004200
	H10	2.68087600	−0.64340200	0.84494100
	H11	2.68088500	−0.64361400	−0.84467900
	H12	−0.56417700	1.73193800	−0.00028000
Anion	N1	1.09782300	−0.25964600	0.00008600
	N2	0.58607900	0.96336400	−0.00000300
	N3	−0.73534900	0.85500500	−0.00008100
	N4	−1.04076500	−0.43504700	0.00013400
	N5	0.09221200	−1.12367700	−0.00013600

SCF Done: E(B3LYP, cation) = −360.0167181 a.u.

SCF Done: E(B3LYP, anion) = −273.8228097 a.u.

Table S6: Salt 6

Cartesian coordinates

	Atom	X	Y	Z
Cation	N1	0.00000000	0.00000600	0.00000000
	C2	1.23093900	0.87042100	0.00000000
	C3	−1.23094000	0.87042000	0.00000000
	C4	0.00000000	−0.87043100	1.23096600
	C5	0.00000000	−0.87043100	−1.23096600
	H6	2.11354200	0.23304200	0.00000000
	H7	1.22150400	1.49452800	0.89199100
	H8	1.22150400	1.49452800	−0.89199100
	H9	−2.11354200	0.23304200	0.00000000
	H10	−1.22150500	1.49452800	−0.89199000
	H11	−1.22150500	1.49452800	0.89199000
	H12	0.00021300	−0.23302500	2.11354600
	H13	0.89189400	−1.49466900	1.22140400
	H14	−0.89210800	−1.49436600	1.22161700
	H15	0.00021300	−0.23302500	−2.11354600
	H16	−0.89210800	−1.49436600	−1.22161700
	H17	0.89189400	−1.49466900	−1.22140400
Anion	N1	1.09782300	−0.25964600	0.00008600
	N2	0.58607900	0.96336400	−0.00000300
	N3	−0.73534900	0.85500500	−0.00008100
	N4	−1.04076500	−0.43504700	0.00013400
	N5	0.09221200	−1.12367700	−0.00013600

SCF Done: E(B3LYP, cation) = −214.2233642 a.u.

SCF Done: E(B3LYP, anion) = −273.8228097 a.u.

Table S7: Salt 7

Cartesian coordinates

	Atom	X	Y	Z
Cation	N1	0.01505600	1.33482600	−0.00016400
	C2	0.00005600	0.00000600	0.00002800
	N3	1.14853800	−0.68050900	0.00012700
	N4	−1.16365100	−0.65433800	−0.00009900
	H5	0.86148500	1.85114100	−0.18670200
	H6	−0.81953400	1.86956400	0.18784900
	H7	2.02923800	−0.22498500	0.18640200

	H8	1.17252800	−1.67159400	−0.18697800
	H9	−1.20975100	−1.64457800	0.18733500
	H10	−2.03389600	−0.17943500	−0.18712000
Anion	N1	1.09782300	−0.25964600	0.00008600
	N2	0.58607900	0.96336400	−0.00000300
	N3	−0.73534900	0.85500500	−0.00008100
	N4	−1.04076500	−0.43504700	0.00013400
	N5	0.09221200	−1.12367700	−0.00013600

SCF Done: E(B3LYP, cation) = −205.8356358 a.u.

SCF Done: E(B3LYP, anion) = −273.8228097 a.u.

Table S8: Salt 8

Cartesian coordinates

	Atom	X	Y	Z
Cation	N1	−0.05049000	−1.92874500	0.00000000
	N2	−0.63398200	−0.66960600	0.00000000
	C3	0.00000000	0.49077800	0.00000000
	N4	−0.71303500	1.63576100	0.00000000
	N5	1.34090500	0.55396100	0.00000000
	H6	−0.67184900	−2.71566800	0.00000000
	H7	0.94319800	−2.04223100	0.00000000
	H8	−1.64306200	−0.68135400	0.00000000
	H9	−0.25481800	2.53243700	0.00000000
	H10	−1.72031300	1.63950200	0.00000000
	H11	1.81372800	1.44388600	0.00000000
	H12	1.92932800	−0.26084100	0.00000000
Anion	N1	1.09782300	−0.25964600	0.00008600
	N2	0.58607900	0.96336400	−0.00000300
	N3	−0.73534900	0.85500500	−0.00008100
	N4	−1.04076500	−0.43504700	0.00013400
	N5	0.09221200	−1.12367700	−0.00013600

SCF Done: E(B3LYP, cation) = −261.143769 a.u.

SCF Done: E(B3LYP, anion) = −273.8228097 a.u.

Table S9: Salt 9

Cartesian coordinates

	Atom	X	Y	Z
Cation	N1	0.00000000	2.42944600	−0.09893800
	N2	0.00000000	1.14998700	−0.65450900

	C3	0.00000000	0.00000000	0.02528500
	N4	0.00000000	−1.14998700	−0.65450900
	N5	0.00000000	−2.42944600	−0.09893800
	N6	0.00000000	0.00000000	1.37233500
	H7	0.00000000	3.19851000	−0.74127800
	H8	0.00000000	2.56996300	0.89042200
	H9	0.00000000	1.13876300	−1.66204000
	H10	0.00000000	−1.13876300	−1.66204000
	H11	0.00000000	−3.19851000	−0.74127800
	H12	0.00000000	−2.56996300	0.89042200
	H13	0.00000000	−0.84963300	1.90800300
	H14	0.00000000	0.84963300	1.90800300
Anion	N1	1.09782300	−0.25964600	0.00008600
	N2	0.58607900	0.96336400	−0.00000300
	N3	−0.73534900	0.85500500	−0.00008100
	N4	−1.04076500	−0.43504700	0.00013400
	N5	0.09221200	−1.12367700	−0.00013600

SCF Done: E(B3LYP, cation) = −316.4471704 a.u.

SCF Done: E(B3LYP, anion) = −273.8228097 a.u.

Table S10: Salt 10

Cartesian coordinates

	Atom	X	Y	Z
Cation	N1	−2.30865200	0.81588200	0.00000000
	C2	−1.21694900	0.03718400	0.00000000
	N3	0.00000000	0.64962600	0.00000000
	C4	1.27171000	0.00451000	0.00000000
	N5	2.30605800	0.88525700	0.00000000
	N6	−1.29726400	−1.27578000	0.00000000
	N7	1.30701300	−1.27093700	0.00000000
	H8	−3.23217400	0.41098800	0.00000000
	H9	−2.25308100	1.82198700	0.00000000
	H10	−0.00498400	1.65842700	0.00000000
	H11	3.25154100	0.53751800	0.00000000
	H12	2.19157500	1.88552200	0.00000000
	H13	−0.38525300	−1.77463900	0.00000000
	H14	−2.18726700	−1.75082900	0.00000000
	H15	2.24099900	−1.66747000	0.00000000
Anion	N1	1.09782300	−0.25964600	0.00008600
	N2	0.58607900	0.96336400	−0.00000300
	N3	−0.73534900	0.85500500	−0.00008100

	N4	−1.04076500	−0.43504700	0.00013400
	N5	0.09221200	−1.12367700	−0.00013600

SCF Done: E(B3LYP, cation) = −354.7051979 a.u.

SCF Done: E(B3LYP, anion) = −273.8228097 a.u.

Table S11: Salt 11

Cartesian coordinates

	Atom	X	Y	Z
Cation	N1	2.31218400	0.76862300	0.00000000
	C2	1.20229600	0.02255600	0.00000000
	N3	0.00000000	0.66359500	0.00000000
	N4	1.26145600	−1.29536300	0.00000000
	C5	−1.27820400	0.00713000	0.00000000
	N6	−2.32216300	0.85491600	0.00000000
	O7	−1.35801900	−1.20338400	0.00000000
	H8	3.22630700	0.34135400	0.00000000
	H9	2.28234200	1.77628500	0.00000000
	H10	0.02372300	1.67372500	0.00000000
	H11	0.37690600	−1.80805400	0.00000000
	H12	2.14512200	−1.78250700	0.00000000
	H13	−3.24569300	0.44611200	0.00000000
	H14	−2.24945800	1.85963800	0.00000000
Anion	N1	1.09782300	−0.25964600	0.00008600
	N2	0.58607900	0.96336400	−0.00000300
	N3	−0.73534900	0.85500500	−0.00008100
	N4	−1.04076500	−0.43504700	0.00013400
	N5	0.09221200	−1.12367700	−0.00013600

SCF Done: E(B3LYP, cation) = −374.5987024 a.u.

SCF Done: E(B3LYP, anion) = −273.8228097 a.u.

Table S12: Salt 12

Cartesian coordinates

	Atom	X	Y	Z
Cation	N1	1.75806900	−0.57488600	−0.00004200
	C2	0.75090200	0.29866000	−0.00001000
	N3	3.04773100	−0.13300200	−0.00006300
	C4	−0.61057300	−0.33673600	0.00000800
	N5	−1.59966500	0.63307700	0.00006400
	N6	−2.90571200	0.10701600	0.00005300

	O7	0.85966500	1.52738700	0.00000000
	O8	−0.92031800	−1.51553200	−0.00000500
	H9	1.59160400	−1.57331100	−0.00005100
	H10	3.79088500	−0.80584800	−0.00010800
	H11	3.17878900	0.86522600	−0.00007000
	H12	−1.41787600	1.63278400	0.00005000
	H13	−2.72626200	−0.93289200	0.00000000
	H14	−3.43841300	0.36106800	0.84132500
	H15	−3.43843900	0.36115900	−0.84117500
Anion	N1	1.09782300	−0.25964600	0.00008600
	N2	0.58607900	0.96336400	−0.00000300
	N3	−0.73534900	0.85500500	−0.00008100
	N4	−1.04076500	−0.43504700	0.00013400
	N5	0.09221200	−1.12367700	−0.00013600

SCF Done: E(B3LYP, cation) = −449.709776 a.u.

SCF Done: E(B3LYP, anion) = −273.8228097 a.u.

Table S13: Salt 13

Cartesian coordinates

	Atom	X	Y	Z
Cation	N1	4.64460300	−0.37096200	−0.00044300
	O2	3.25611800	−0.58436800	−0.00074100
	C3	2.52993300	0.69044700	0.00071400
	C4	1.04921100	0.39610900	0.00038100
	C5	0.53723900	−0.90192500	0.00052500
	C6	−0.84111500	−1.10432300	0.00032500
	C7	−1.67996700	−0.00034900	0.00002700
	C8	−1.19170200	1.30107300	−0.00009600
	C9	0.18320000	1.49560700	0.00005000
	N10	−3.15315100	−0.21520400	−0.00017500
	O11	−3.85972300	0.78002800	−0.00040700
	O12	−3.54675200	−1.37061900	−0.00003800
	H13	5.06848300	−1.30744800	−0.00141600
	H14	4.95422600	0.13708900	−0.84378600
	H15	4.95413900	0.13530500	0.84400500
	H16	2.82173100	1.24766000	0.89925300
	H17	2.82166600	1.24967900	−0.89658200
	H18	1.19402000	−1.76144200	0.00079700
	H19	−1.26422700	−2.09961600	0.00041100
	H20	−1.88166200	2.13385400	−0.00034000
	H21	0.57351900	2.50792900	−0.00008800

Anion	N1	1.09782300	−0.25964600	0.00008600
	N2	0.58607900	0.96336400	−0.00000300
	N3	−0.73534900	0.85500500	−0.00008100
	N4	−1.04076500	−0.43504700	0.00013400
	N5	0.09221200	−1.12367700	−0.00013600

SCF Done: E(B3LYP, cation) = −607.073813 a.u.

SCF Done: E(B3LYP, anion) = −273.8228097 a.u.

Table S14: Salt 14

Cartesian coordinates

	Atom	X	Y	Z
Cation	C1	0.50712200	−0.53528200	0.00000000
	N2	−0.56713100	−1.34638000	0.00000000
	N3	−1.73971000	−0.65351600	0.00000000
	C4	−1.38184300	0.59048800	0.00000000
	N5	0.00000000	0.71419300	0.00000000
	N6	0.70444300	1.89288900	0.00000000
	N7	1.80014100	−0.88815100	0.00000000
	H8	−0.58523700	−2.35710100	0.00000000
	H9	−2.05097700	1.43600200	0.00000000
	H10	0.17789700	2.74782300	0.00000000
	H11	1.70633800	1.88155500	0.00000000
	H12	2.54646300	−0.21278800	0.00000000
	H13	2.06964100	−1.85997500	0.00000000
Anion	N1	1.09782300	−0.25964600	0.00008600
	N2	0.58607900	0.96336400	−0.00000300
	N3	−0.73534900	0.85500500	−0.00008100
	N4	−1.04076500	−0.43504700	0.00013400
	N5	0.09221200	−1.12367700	−0.00013600

SCF Done: E(B3LYP, cation) = −353.3810613 a.u.

SCF Done: E(B3LYP, anion) = −273.8228097 a.u.

Table S15: Salt 15

Cartesian coordinates

	Atom	X	Y	Z
Cation	N1	−0.60776300	−0.42370200	−0.00090700
	C2	−0.03648700	0.82050400	−0.00617700
	N3	1.31644700	0.57678700	−0.01788400
	C4	1.45163800	−0.82638600	−0.01120500

	N5	0.30139100	−1.47084700	−0.01158900
	C6	−1.94323300	−0.25822900	0.00235500
	N7	−2.09938700	1.08148500	−0.00046200
	N8	−0.89685800	1.79142900	−0.00677900
	N9	2.28479100	1.55951000	0.02345500
	N10	2.65887200	−1.40721700	0.03767200
	N11	−2.86076400	−1.22861600	0.01233500
	H12	−2.96413300	1.60239100	0.00883900
	H13	2.83875100	1.53061600	0.87133500
	H14	2.82928700	1.61557700	−0.82886500
	H15	2.70933100	−2.41271800	−0.02969500
	H16	3.49074100	−0.88102600	−0.17671300
	H17	−3.84768700	−1.02850500	−0.00868100
	H18	−2.56489700	−2.19347000	0.00305200
Anion	N1	1.09782300	−0.25964600	0.00008600
	N2	0.58607900	0.96336400	−0.00000300
	N3	−0.73534900	0.85500500	−0.00008100
	N4	−1.04076500	−0.43504700	0.00013400
	N5	0.09221200	−1.12367700	−0.00013600

SCF Done: E(B3LYP, cation) = −556.4008576 a.u.

SCF Done: E(B3LYP, anion) = −273.8228097 a.u.

Table S16: Salt 16

Cartesian coordinates

	Atom	X	Y	Z
Cation	N1	−2.36597700	−1.56459900	0.00000500
	C2	−2.85380700	−0.32148600	0.00000300
	N3	−1.83383700	0.58129700	−0.00000300
	C4	−0.69199400	−0.18874700	−0.00000300
	N5	−1.02574000	−1.47083200	0.00000200
	N6	−4.16968300	−0.01110300	0.00000300
	C7	0.65231300	0.29383200	0.00000000
	N8	1.05072300	1.54373000	0.00000500
	N9	2.41535300	1.46842300	0.00000500
	C10	2.85382700	0.19737000	0.00000000
	N11	1.74893800	−0.57062000	−0.00000100
	N12	−1.90491700	1.95699400	−0.00002500
	N13	1.67092700	−1.93908100	−0.00000300
	N14	4.12649300	−0.22950300	−0.00000100
	H15	−4.82868300	−0.77277200	0.00000300
	H16	−4.52781600	0.92607700	0.00000700

	Atom	X	Y	Z
	H17	2.96473700	2.31497400	0.00000700
	H18	−2.80467200	2.39575300	0.00002400
	H19	−1.03638400	2.46348700	0.00007300
	H20	0.71532600	−2.30403300	−0.00000200
	H21	2.51498000	−2.47926000	−0.00000900
	H22	4.36191900	−1.20780100	−0.00000400
	H23	4.89260200	0.42480700	0.00000000
Anion	N1	1.09782300	−0.25964600	0.00008600
	N2	0.58607900	0.96336400	−0.00000300
	N3	−0.73534900	0.85500500	−0.00008100
	N4	−1.04076500	−0.43504700	0.00013400
	N5	0.09221200	−1.12367700	−0.00013600

SCF Done: E(B3LYP, cation) = −705.2135034 a.u.

SCF Done: E(B3LYP, anion) = −273.8228097 a.u.

Table S17: Salt 17

Cartesian coordinates

	Atom	X	Y	Z
Cation	C1	−2.76619800	−0.04963200	0.00089200
	N2	−2.75984000	−1.33372300	0.40073900
	N3	−1.50077000	−1.87154300	0.45055700
	C4	−0.72367600	−0.91568500	0.06460000
	N5	−1.45002000	0.25318900	−0.20725900
	N6	−3.78002900	0.77491400	−0.17698600
	C7	0.72362900	−0.91569600	−0.06428900
	N8	1.50060900	−1.87154300	−0.45054600
	N9	2.75970400	−1.33381800	−0.40082600
	C10	2.76618200	−0.04971800	−0.00099200
	N11	1.45007400	0.25311600	0.20751500
	N12	3.78006800	0.77483700	0.17657400
	N13	−0.98754100	1.43355200	−0.77716800
	C14	−0.00001200	2.20388400	0.00008100
	N15	0.98777300	1.43360000	0.77721100
	H16	−3.54944100	−1.91639600	0.65767800
	H17	−3.60678500	1.70716000	−0.53312400
	H18	−4.73954900	0.48830400	−0.03444200
	H19	3.54926900	−1.91658800	−0.65765700
	H20	3.60695100	1.70698000	0.53304700
	H21	4.73953100	0.48827800	0.03355400
	H22	−0.70666700	1.26518300	−1.73907900
	H23	0.49793700	2.84245900	−0.73074300

	H24	−0.49812100	2.84212100	0.73109200
	H25	0.70714500	1.26552200	1.73924100
Anion	N1	1.09782300	−0.25964600	0.00008600
	N2	0.58607900	0.96336400	−0.00000300
	N3	−0.73534900	0.85500500	−0.00008100
	N4	−1.04076500	−0.43504700	0.00013400
	N5	0.09221200	−1.12367700	−0.00013600

SCF Done: E(B3LYP, cation) = −743.608022 a.u.

SCF Done: E(B3LYP, anion) = −273.8228097 a.u.

Table S18: Salt 18

Cartesian coordinates

	Atom	X	Y	Z
Cation	C1	−1.95998000	1.08037900	0.00004000
	N2	−0.74863600	1.56336400	0.00001600
	N3	0.06046900	0.47142700	−0.00004600
	C4	−0.65089200	−0.64528600	−0.00017800
	N5	−1.94883600	−0.29194300	−0.00002700
	C6	−3.11298100	−1.19686300	0.00008600
	C7	3.81669700	−0.39891300	0.00006300
	C8	2.30519100	−0.65934400	0.00004000
	C9	1.53314100	0.65473400	−0.00004000
	H10	−2.86086800	1.67373700	0.00012300
	H11	−0.26884000	−1.65229700	−0.00030400
	H12	−4.01466800	−0.58833100	0.00008800
	H13	−3.09629700	−1.81745400	−0.89494700
	H14	−3.09622100	−1.81735500	0.89518800
	H15	4.36083800	−1.34425700	0.00012200
	H16	4.12464500	0.16268700	−0.88520800
	H17	4.12460300	0.16277200	0.88529600
	H18	2.04010400	−1.24873200	0.88485900
	H19	2.04015200	−1.24881800	−0.88473600
	H20	1.75926500	1.25488300	−0.88340000
	H21	1.75925200	1.25498800	0.88325100
Anion	N1	−0.66794700	−1.46327300	−0.00018400
	C2	−1.01162700	−0.16002100	0.00007000
	N3	−0.00000100	0.70958100	0.00031400
	C4	1.01162700	−0.16002300	0.00014800
	N5	0.66796000	−1.46327800	0.00043900
	N6	−2.40065100	0.26300100	−0.00000300
	O7	−3.26725400	−0.60961200	−0.00035500

	O8	−2.63610000	1.46942000	0.00013900
	N9	2.40064900	0.26300400	−0.00012800
	O10	2.63608900	1.46942400	−0.00020900
	O11	3.26725500	−0.60960600	−0.00012200

SCF Done: E(B3LYP, cation) = −399.9844557 a.u.

SCF Done: E(B3LYP, anion) = −650.9218673 a.u.

Table S19: Salt 19

Cartesian coordinates

	Atom	X	Y	Z
Cation	C1	2.87140500	−0.88054800	−0.00055200
	N2	1.74972100	−1.54544900	0.00034800
	N3	0.78140300	−0.59207600	0.00019000
	C4	1.31251200	0.62241300	0.00070400
	N5	2.64831200	0.47329200	−0.00004300
	C6	3.65971500	1.54792600	−0.00019800
	C7	−0.63314800	−1.01825800	0.00005800
	C8	−1.60874400	0.15515800	−0.00004600
	N9	−2.93234200	−0.47322400	−0.00014300
	N10	−3.89400400	0.30831600	−0.00012000
	N11	−4.85266100	0.90457100	−0.00011000
	H12	3.85274600	−1.32859400	−0.00111200
	H13	0.78368200	1.56121400	0.00116100
	H14	4.64407400	1.08517200	−0.00101400
	H15	3.54643000	2.15804800	−0.89529600
	H16	3.54753800	2.15728000	0.89556300
	H17	−0.77820900	−1.63921600	0.88444600
	H18	−0.77808600	−1.63927000	−0.88431300
	H19	−1.46573100	0.77858400	−0.89304500
	H20	−1.46588400	0.77862600	0.89294800
Anion	N1	−0.66794700	−1.46327300	−0.00018400
	C2	−1.01162700	−0.16002100	0.00007000
	N3	−0.00000100	0.70958100	0.00031400
	C4	1.01162700	−0.16002300	0.00014800
	N5	0.66796000	−1.46327800	0.00043900
	N6	−2.40065100	0.26300100	−0.00000300
	O7	−3.26725400	−0.60961200	−0.00035500
	O8	−2.63610000	1.46942000	0.00013900
	N9	2.40064900	0.26300400	−0.00012800
	O10	2.63608900	1.46942400	−0.00020900
	O11	3.26725500	−0.60960600	−0.00012200

SCF Done: E(B3LYP, cation) = −524.2704084 a.u.

SCF Done: E(B3LYP, anion) = −650.9218673 a.u.

Table S20: Salt 20

Cartesian coordinates

	Atom	X	Y	Z
Cation	C1	−1.09878300	1.10871700	−0.00090000
	N2	−2.39547400	1.25769000	−0.00096500
	N3	−2.88786000	−0.00653400	0.00039300
	C4	−1.90913800	−0.89798900	−0.00035000
	N5	−0.74840800	−0.22007700	−0.00087900
	C6	0.60181300	−0.84175200	−0.00148600
	C7	1.72874200	0.18909300	0.00021400
	N8	2.94961000	−0.62150000	−0.00032300
	N9	4.01357700	0.01498300	0.00061800
	N10	5.04752200	0.46730400	0.00136000
	C11	−4.33852600	−0.23320300	0.00187100
	H12	−0.39638400	1.92535400	−0.00137100
	H13	−2.01832000	−1.97091800	−0.00007500
	H14	0.68153100	−1.46967200	−0.88954900
	H15	0.68113600	−1.47206100	0.88491400
	H16	1.67130600	0.82437900	0.89343000
	H17	1.67202000	0.82657700	−0.89147900
	H18	−4.52389900	−1.30550300	0.00242000
	H19	−4.76158200	0.22511000	0.89436500
	H20	−4.76321300	0.22446800	−0.89017200
Anion	N1	−0.66794700	−1.46327300	−0.00018400
	C2	−1.01162700	−0.16002100	0.00007000
	N3	−0.00000100	0.70958100	0.00031400
	C4	1.01162700	−0.16002300	0.00014800
	N5	0.66796000	−1.46327800	0.00043900
	N6	−2.40065100	0.26300100	−0.00000300
	O7	−3.26725400	−0.60961200	−0.00035500
	O8	−2.63610000	1.46942000	0.00013900
	N9	2.40064900	0.26300400	−0.00012800
	O10	2.63608900	1.46942400	−0.00020900
	O11	3.26725500	−0.60960600	−0.00012200

SCF Done: E(B3LYP, cation) = −524.2708654 a.u.

SCF Done: E(B3LYP, anion) = −650.9218673 a.u.

Table S21: Salt 21

Cartesian coordinates

	Atom	X	Y	Z
Cation	C1	0.18477700	−0.48806600	−0.00017100
	N2	−0.95937800	−1.14105000	−0.00018500
	N3	−1.91196800	−0.18023500	0.00005300
	C4	−1.38445400	1.02649200	0.00005300
	N5	−0.04134400	0.88642500	−0.00005800
	C6	0.89524300	2.03075200	−0.00012200
	C7	−3.32873300	−0.56230300	0.00021500
	N8	1.34903900	−1.20352600	−0.00035600
	N9	2.47345600	−0.67220400	0.00009700
	N10	3.56526000	−0.39981600	0.00040300
	H11	−1.91807500	1.96378500	0.00016800
	H12	1.91877100	1.67476400	−0.00053300
	H13	0.73147200	2.62963000	−0.89531300
	H14	0.73204600	2.62928600	0.89540500
	H15	−3.93391500	0.34219600	0.00043500
	H16	−3.52851500	−1.15391500	−0.89190000
	H17	−3.52824900	−1.15417100	0.89221900
Anion	N1	−0.66794700	−1.46327300	−0.00018400
	C2	−1.01162700	−0.16002100	0.00007000
	N3	−0.00000100	0.70958100	0.00031400
	C4	1.01162700	−0.16002300	0.00014800
	N5	0.66796000	−1.46327800	0.00043900
	N6	−2.40065100	0.26300100	−0.00000300
	O7	−3.26725400	−0.60961200	−0.00035500
	O8	−2.63610000	1.46942000	0.00013900
	N9	2.40064900	0.26300400	−0.00012800
	O10	2.63608900	1.46942400	−0.00020900
	O11	3.26725500	−0.60960600	−0.00012200

SCF Done: E(B3LYP, cation) = −484.9450554 a.u.

SCF Done: E(B3LYP, anion) = −650.9218673 a.u.

Table S22: Salt 22

Cartesian coordinates

	Atom	X	Y	Z
Cation	C1	2.89223000	−0.84681300	−0.00007600
	N2	1.76828200	−1.51421700	−0.00001800
	N3	0.79660100	−0.58247900	0.00009300

	C4	1.31177700	0.64987800	0.00011300
	N5	2.64710500	0.50518900	0.00000300
	N6	3.58043400	1.49862900	−0.00002800
	C7	−0.61547700	−1.01884600	0.00021400
	C8	−1.59780800	0.14905600	−0.00009200
	N9	−2.91800400	−0.48602400	−0.00001700
	N10	−3.88380400	0.29003300	−0.00007800
	N11	−4.84616700	0.88045200	−0.00012200
	H12	3.87888200	−1.28201500	−0.00017500
	H13	0.77753900	1.58442500	0.00019900
	H14	4.54962400	1.23410500	−0.00012000
	H15	3.27341800	2.45497100	0.00003500
	H16	−0.75866400	−1.63903700	0.88546900
	H17	−0.75865400	−1.63948000	−0.88473100
	H18	−1.45875200	0.77294300	−0.89346700
	H19	−1.45884600	0.77335200	0.89301100
Anion	N1	−0.66794700	−1.46327300	−0.00018400
	C2	−1.01162700	−0.16002100	0.00007000
	N3	−0.00000100	0.70958100	0.00031400
	C4	1.01162700	−0.16002300	0.00014800
	N5	0.66796000	−1.46327800	0.00043900
	N6	−2.40065100	0.26300100	−0.00000300
	O7	−3.26725400	−0.60961200	−0.00035500
	O8	−2.63610000	1.46942000	0.00013900
	N9	2.40064900	0.26300400	−0.00012800
	O10	2.63608900	1.46942400	−0.00020900
	O11	3.26725500	−0.60960600	−0.00012200

SCF Done: E(B3LYP, cation) = −540.2671406 a.u.

SCF Done: E(B3LYP, anion) = −650.9218673 a.u.

Table S23: Salt 23

Cartesian coordinates

	Atom	X	Y	Z
Cation	N1	0.59742900	−1.53950800	−0.00004700
	N2	−0.66881200	−1.53321300	0.00000800
	N3	−1.07173000	−0.22648800	0.00002800
	C4	−0.00603900	0.58942200	−0.00002500
	N5	1.03461400	−0.24552600	−0.00008400
	N6	−2.39402200	0.18054700	0.00004600
	C7	−0.00401400	2.06752800	−0.00001300
	C8	2.47346300	0.05182800	0.00006500

	H9	−2.85710600	−0.17504600	−0.83326500
	H10	−2.85715400	−0.17540200	0.83317800
	H11	0.51462100	2.44501700	−0.88550100
	H12	−1.02999200	2.43188900	0.00025000
	H13	0.51505600	2.44498800	0.88523500
	H14	2.99048900	−0.90499400	−0.00023500
	H15	2.73067200	0.61539500	−0.89632800
	H16	2.73060000	0.61480100	0.89685300
Anion	N1	−0.66794700	−1.46327300	−0.00018400
	C2	−1.01162700	−0.16002100	0.00007000
	N3	−0.00000100	0.70958100	0.00031400
	C4	1.01162700	−0.16002300	0.00014800
	N5	0.66796000	−1.46327800	0.00043900
	N6	−2.40065100	0.26300100	−0.00000300
	O7	−3.26725400	−0.60961200	−0.00035500
	O8	−2.63610000	1.46942000	0.00013900
	N9	2.40064900	0.26300400	−0.00012800
	O10	2.63608900	1.46942400	−0.00020900
	O11	3.26725500	−0.60960600	−0.00012200

SCF Done: E(B3LYP, cation) = −392.6831906 a.u.

SCF Done: E(B3LYP, anion) = −650.9218673 a.u.

Table S24: Salt 24

Cartesian coordinates

	Atom	X	Y	Z
Cation	C1	3.35468900	−0.29963400	0.00002900
	N2	2.31701600	−1.08583400	−0.00013900
	N3	1.23834300	−0.25134200	0.00000300
	C4	1.61519900	1.01603600	−0.00010100
	N5	2.95905600	1.01426000	0.00006400
	C6	−0.11531200	−0.84712800	0.00011600
	C7	−1.22721400	0.19790700	−0.00003800
	N8	−2.46118600	−0.59117300	0.00000800
	N9	−3.51346600	0.06445600	0.00000200
	N10	−4.53954000	0.53461700	0.00000100
	H11	4.38281200	−0.62629100	0.00006900
	H12	0.98218700	1.88791900	−0.00015500
	H13	3.55525000	1.83419200	0.00029900
	H14	−0.18057000	−1.48111300	0.88483800
	H15	−0.18056400	−1.48135200	−0.88443300
	H16	−1.16238800	0.83422900	−0.89330800

	H17	−1.16245200	0.83443800	0.89308700
Anion	C1	0.79717000	0.28342400	0.00000900
	C2	−0.58056800	0.53786700	0.00000200
	N3	−0.81036200	1.84623700	0.00000200
	C4	0.45875900	2.35802000	−0.00000800
	N5	1.45163600	1.47525100	0.00000500
	N6	1.44641200	−0.97668100	0.00001400
	O7	0.72010200	−1.98641900	−0.00001300
	O8	2.67855900	−1.02697400	−0.00000700
	N9	−1.71005700	−0.40124600	0.00000100
	O10	−2.15717700	−0.75001000	1.08450700
	O11	−2.15718700	−0.74999700	−1.08450600
	H12	0.63005500	3.42640600	−0.00001000

SCF Done: E(B3LYP, cation) = −484.9431898 a.u.

SCF Done: E(B3LYP, anion) = −634.882769 a.u.

Table S25: Salt 25

Cartesian coordinates

	Atom	X	Y	Z
Cation	C1	3.35468900	−0.29963400	0.00002900
	N2	2.31701600	−1.08583400	−0.00013900
	N3	1.23834300	−0.25134200	0.00000300
	C4	1.61519900	1.01603600	−0.00010100
	N5	2.95905600	1.01426000	0.00006400
	C6	−0.11531200	−0.84712800	0.00011600
	C7	−1.22721400	0.19790700	−0.00003800
	N8	−2.46118600	−0.59117300	0.00000800
	N9	−3.51346600	0.06445600	0.00000200
	N10	−4.53954000	0.53461700	0.00000100
	H11	4.38281200	−0.62629100	0.00006900
	H12	0.98218700	1.88791900	−0.00015500
	H13	3.55525000	1.83419200	0.00029900
	H14	−0.18057000	−1.48111300	0.88483800
	H15	−0.18056400	−1.48135200	−0.88443300
	H16	−1.16238800	0.83422900	−0.89330800
	H17	−1.16245200	0.83443800	0.89308700
Anion	N1	0.67195600	2.05851800	0.00000000
	N2	−0.67221000	2.05845700	0.00000000
	N3	−1.11928500	0.81026500	0.00000000
	C4	0.00000000	0.07854900	0.00000000
	N5	1.11920100	0.81038500	0.00000000

	N6	0.00008100	−1.36159900	0.00000000
	O7	1.08603400	−1.94390800	0.00000000
	O8	−1.08580900	−1.94402600	0.00000000

SCF Done: E(B3LYP, cation) = −484.9431898 a.u.

SCF Done: E(B3LYP, anion) = −462.3675711 a.u.

Table S26: Salt 26

Cartesian coordinates

	Atom	X	Y	Z
Cation	C1	2.14463400	−0.30409000	0.00000000
	N2	1.08918100	−1.08486500	0.00003300
	N3	0.02309400	−0.23779200	0.00006700
	C4	0.39886900	1.02440800	0.00008400
	N5	1.74898300	1.02511000	0.00001600
	N6	3.41271400	−0.80956000	−0.00007700
	N7	4.37229100	−0.00859800	−0.00004700
	N8	5.34128000	0.56030400	−0.00004300
	C9	−1.33172400	−0.82827600	0.00009500
	C10	−2.44067200	0.21969900	−0.00003200
	N11	−3.67761500	−0.56532300	−0.00002900
	N12	−4.72730800	0.09390800	−0.00004300
	N13	−5.75155400	0.56833300	−0.00005600
	H14	−0.23101400	1.89855300	0.00010800
	H15	2.33162100	1.85408200	0.00011600
	H16	−1.39790900	−1.46273100	0.88444200
	H17	−1.39786600	−1.46289200	−0.88413900
	H18	−2.37442600	0.85589100	−0.89317900
	H19	−2.37450800	0.85602900	0.89302200
Anion	N1	0.67195600	2.05851800	0.00000000
	N2	−0.67221000	2.05845700	0.00000000
	N3	−1.11928500	0.81026500	0.00000000
	C4	0.00000000	0.07854900	0.00000000
	N5	1.11920100	0.81038500	0.00000000
	N6	0.00008100	−1.36159900	0.00000000
	O7	1.08603400	−1.94390800	0.00000000
	O8	−1.08580900	−1.94402600	0.00000000

SCF Done: E(B3LYP, cation) = −648.5639247 a.u.

SCF Done: E(B3LYP, anion) = −462.3675711 a.u.

Table S27: Salt 27

Cartesian coordinates

	Atom	X	Y	Z
Cation	C1	1.91162800	1.01660900	−0.00000500
	C2	2.33196700	−0.26931200	0.00000900
	N3	1.19384000	−1.06547000	−0.00000100
	C4	0.09727700	−0.29383100	−0.00000300
	N5	0.51721300	0.98561500	0.00000100
	N6	−1.14891600	−0.84019600	−0.00000700
	N7	−2.13984200	−0.07334800	0.00000100
	N8	−3.12429200	0.46490500	0.00000600
	H9	2.46023100	1.94259000	−0.00001000
	H10	3.32336300	−0.68820900	0.00001500
	H11	1.16522900	−2.07783300	0.00000300
	H12	−0.08007400	1.80211500	−0.00000100
Anion	C1	0.79717000	0.28342400	0.00000900
	C2	−0.58056800	0.53786700	0.00000200
	N3	−0.81036200	1.84623700	0.00000200
	C4	0.45875900	2.35802000	−0.00000800
	N5	1.45163600	1.47525100	0.00000500
	N6	1.44641200	−0.97668100	0.00001400
	O7	0.72010200	−1.98641900	−0.00001300
	O8	2.67855900	−1.02697400	−0.00000700
	N9	−1.71005700	−0.40124600	0.00000100
	O10	−2.15717700	−0.75001000	1.08450700
	O11	−2.15718700	−0.74999700	−1.08450600
	H12	0.63005500	3.42640600	−0.00001000

SCF Done: E(B3LYP, cation) = −390.2793719 a.u.

SCF Done: E(B3LYP, anion) = −634.882769 a.u.

Table S28: Salt 28

Cartesian coordinates

	Atom	X	Y	Z
Cation	C1	1.06640500	−0.41832800	−0.00003200
	N2	0.03611200	−1.21412900	−0.00000400
	N3	−1.03285300	−0.37700300	0.00003700
	C4	−0.67974900	0.89734900	−0.00005800
	N5	0.65865300	0.89769800	0.00002500
	H6	2.09788600	−0.73449200	−0.00004900
	H7	−1.97156900	−0.76356700	0.00008100
	H8	−1.33192900	1.75728600	−0.00010100

	H9	1.25228900	1.72069000	0.00019900
Anion	C1	0.79717000	0.28342400	0.00000900
	C2	−0.58056800	0.53786700	0.00000200
	N3	−0.81036200	1.84623700	0.00000200
	C4	0.45875900	2.35802000	−0.00000800
	N5	1.45163600	1.47525100	0.00000500
	N6	1.44641200	−0.97668100	0.00001400
	O7	0.72010200	−1.98641900	−0.00001300
	O8	2.67855900	−1.02697400	−0.00000700
	N9	−1.71005700	−0.40124600	0.00000100
	O10	−2.15717700	−0.75001000	1.08450700
	O11	−2.15718700	−0.74999700	−1.08450600
	H12	0.63005500	3.42640600	−0.00001000

SCF Done: E(B3LYP, cation) = −242.6694066 a.u.

SCF Done: E(B3LYP, anion) = −634.882769 a.u.

Table S29: Salt 29

Cartesian coordinates

	Atom	X	Y	Z
Cation	C1	−1.34395300	−0.76133900	−0.00002500
	N2	−0.09641400	−1.13546600	0.00009700
	N3	0.61937000	0.02363800	−0.00000100
	C4	−0.17515000	1.07971800	0.00011100
	N5	−1.43290700	0.60853900	−0.00005400
	C6	2.08835000	−0.02286700	−0.00006700
	H7	−2.19345800	−1.42615900	−0.00006900
	H8	0.12266600	2.11627900	0.00017900
	H9	−2.27850900	1.16781500	−0.00027500
	H10	2.46870200	0.99662400	−0.00024600
	H11	2.41742300	−0.55216200	0.89275000
	H12	2.41735100	−0.55244700	−0.89274100
Anion	C1	0.79717000	0.28342400	0.00000900
	C2	−0.58056800	0.53786700	0.00000200
	N3	−0.81036200	1.84623700	0.00000200
	C4	0.45875900	2.35802000	−0.00000800
	N5	1.45163600	1.47525100	0.00000500
	N6	1.44641200	−0.97668100	0.00001400
	O7	0.72010200	−1.98641900	−0.00001300
	O8	2.67855900	−1.02697400	−0.00000700
	N9	−1.71005700	−0.40124600	0.00000100
	O10	−2.15717700	−0.75001000	1.08450700

	O11	−2.15718700	−0.74999700	−1.08450600
	H12	0.63005500	3.42640600	−0.00001000

SCF Done: E(B3LYP, cation) = −282.0020614 a.u.

SCF Done: E(B3LYP, anion) = −634.882769 a.u.

Table S30: Salt 30

Cartesian coordinates

	Atom	X	Y	Z
Cation	C1	0.10928300	−0.37050500	−0.00001000
	N2	1.16549400	−1.15216600	−0.00009000
	N3	2.21450300	−0.29340200	0.00006400
	C4	1.85243700	0.97271400	−0.00003500
	N5	0.50786700	0.96531600	0.00002000
	N6	−1.15653700	−0.87371600	0.00003400
	N7	−2.11886300	−0.07468100	0.00000600
	N8	−3.09035500	0.48886000	0.00000100
	H9	3.15781300	−0.66781200	0.00015800
	H10	2.49471700	1.83993100	−0.00001900
	H11	−0.07760900	1.79315200	−0.00011500
Anion	C1	0.79717000	0.28342400	0.00000900
	C2	−0.58056800	0.53786700	0.00000200
	N3	−0.81036200	1.84623700	0.00000200
	C4	0.45875900	2.35802000	−0.00000800
	N5	1.45163600	1.47525100	0.00000500
	N6	1.44641200	−0.97668100	0.00001400
	O7	0.72010200	−1.98641900	−0.00001300
	O8	2.67855900	−1.02697400	−0.00000700
	N9	−1.71005700	−0.40124600	0.00000100
	O10	−2.15717700	−0.75001000	1.08450700
	O11	−2.15718700	−0.74999700	−1.08450600
	H12	0.63005500	3.42640600	−0.00001000

SCF Done: E(B3LYP, cation) = −406.2900901 a.u.

SCF Done: E(B3LYP, anion) = −634.882769 a.u.

Table S31: Salt 31

Cartesian coordinates

	Atom	X	Y	Z
Cation	C1	−0.32893900	−0.28721400	0.00001400
	N2	0.79794200	−0.96170600	0.00000800

	N3	1.77356600	−0.01366000	−0.00001900
	C4	1.27830400	1.20574900	0.00001500
	N5	−0.06489500	1.07521800	0.00004500
	N6	−1.54183000	−0.91313700	0.00004800
	N7	−2.57474900	−0.20924800	−0.00001300
	N8	−3.59430700	0.26297300	−0.00004500
	C9	3.18197800	−0.42974100	−0.00002900
	H10	1.82897400	2.13304200	0.00003700
	H11	−0.72595600	1.84313500	−0.00010900
	H12	3.80813200	0.46031100	−0.00018500
	H13	3.36539900	−1.02604800	0.89239800
	H14	3.36530300	−1.02629200	−0.89231100
Anion	C1	0.79717000	0.28342400	0.00000900
	C2	−0.58056800	0.53786700	0.00000200
	N3	−0.81036200	1.84623700	0.00000200
	C4	0.45875900	2.35802000	−0.00000800
	N5	1.45163600	1.47525100	0.00000500
	N6	1.44641200	−0.97668100	0.00001400
	O7	0.72010200	−1.98641900	−0.00001300
	O8	2.67855900	−1.02697400	−0.00000700
	N9	−1.71005700	−0.40124600	0.00000100
	O10	−2.15717700	−0.75001000	1.08450700
	O11	−2.15718700	−0.74999700	−1.08450600
	H12	0.63005500	3.42640600	−0.00001000

SCF Done: E(B3LYP, cation) = −445.6228493 a.u.

SCF Done: E(B3LYP, anion) = −634.882769 a.u.

Table S32: Salt 32

Cartesian coordinates

	Atom	X	Y	Z
Cation	N1	0.72573800	−1.43573300	0.00000000
	C2	1.10022700	−0.18529000	0.00000000
	N3	0.00000000	0.65115400	0.00000000
	C4	−1.08228900	−0.13710400	0.00000000
	N5	−0.61573600	−1.38850300	0.00000000
	N6	0.02662600	2.01061000	0.00000000
	H7	2.12050200	0.16391300	0.00000000
	H8	−2.11338600	0.17676100	0.00000000
	H9	−1.15210300	−2.24869500	0.00000000
	H10	0.92340300	2.46433500	0.00000000
	H11	−0.84244300	2.51536000	0.00000000

Anion	C1	0.79717000	0.28342400	0.00000900
	C2	−0.58056800	0.53786700	0.00000200
	N3	−0.81036200	1.84623700	0.00000200
	C4	0.45875900	2.35802000	−0.00000800
	N5	1.45163600	1.47525100	0.00000500
	N6	1.44641200	−0.97668100	0.00001400
	O7	0.72010200	−1.98641900	−0.00001300
	O8	2.67855900	−1.02697400	−0.00000700
	N9	−1.71005700	−0.40124600	0.00000100
	O10	−2.15717700	−0.75001000	1.08450700
	O11	−2.15718700	−0.74999700	−1.08450600
	H12	0.63005500	3.42640600	−0.00001000

SCF Done: E(B3LYP, cation) = −297.9953294 a.u.

SCF Done: E(B3LYP, anion) = −634.882769 a.u.

Table S33: Salt 33

Cartesian coordinates

	Atom	X	Y	Z
Cation	C1	−0.21497100	0.71391700	−0.00002900
	N2	−0.31228300	−0.62561700	−0.00019000
	N3	0.92846700	−1.23600100	−0.00004200
	C4	1.76605100	−0.25487100	0.00003500
	N5	1.11344200	0.97170900	0.00022200
	N6	−1.23484900	1.56243700	0.00002300
	N7	−1.52533600	−1.28624200	0.00006300
	H8	2.84023500	−0.35221900	0.00017300
	H9	1.54903700	1.88443600	−0.00106700
	H10	−2.17216900	1.18116500	−0.00010400
	H11	−1.10937000	2.56245200	0.00013800
	H12	−1.60005800	−1.86694400	0.83220300
	H13	−1.60024000	−1.86717100	−0.83190300
Anion	C1	0.79717000	0.28342400	0.00000900
	C2	−0.58056800	0.53786700	0.00000200
	N3	−0.81036200	1.84623700	0.00000200
	C4	0.45875900	2.35802000	−0.00000800
	N5	1.45163600	1.47525100	0.00000500
	N6	1.44641200	−0.97668100	0.00001400
	O7	0.72010200	−1.98641900	−0.00001300
	O8	2.67855900	−1.02697400	−0.00000700
	N9	−1.71005700	−0.40124600	0.00000100
	O10	−2.15717700	−0.75001000	1.08450700

	O11	−2.15718700	−0.74999700	−1.08450600
	H12	0.63005500	3.42640600	−0.00001000

SCF Done: E(B3LYP, cation) = −353.414357 a.u.

SCF Done: E(B3LYP, anion) = −634.882769 a.u.

Table S34: Salt 34

Cartesian coordinates

	Atom	X	Y	Z
Cation	C1	1.30897900	−0.76354400	0.00027600
	N2	0.05235900	−1.13072300	−0.00042700
	N3	−0.64226500	0.03501800	−0.00008200
	C4	0.15440800	1.09354800	−0.00026700
	N5	1.41502700	0.59184600	0.00011800
	N6	−1.98939000	−0.02302000	−0.00005200
	H7	2.14407700	−1.44581400	0.00053200
	H8	−0.13228000	2.13167200	−0.00047700
	H9	2.26724800	1.13951000	0.00045500
	H10	−2.38904500	−0.94767400	0.00093900
	H11	−2.52043400	0.83044200	0.00159200
Anion	C1	0.06990000	0.00372200	0.00003400
	N2	0.77179900	−1.13794900	0.00010800
	N3	2.05955200	−0.74304900	−0.00007000
	C4	2.02817300	0.60943000	−0.00009400
	N5	0.79932800	1.13670200	0.00014500
	N6	−1.37056900	0.01090600	−0.00006900
	O7	−1.95075800	1.10172600	−0.00002400
	O8	−1.96690600	−1.07057200	−0.00001100
	H9	2.93210800	1.20559000	−0.00015700

SCF Done: E(B3LYP, cation) = −298.0042867 a.u.

SCF Done: E(B3LYP, anion) = −446.3474789 a.u.

Table S35: Salt 35

Cartesian coordinates

	Atom	X	Y	Z
Cation	C1	1.91162800	1.01660900	−0.00000500
	C2	2.33196700	−0.26931200	0.00000900
	N3	1.19384000	−1.06547000	−0.00000100
	C4	0.09727700	−0.29383100	−0.00000300
	N5	0.51721300	0.98561500	0.00000100

	N6	−1.14891600	−0.84019600	−0.00000700
	N7	−2.13984200	−0.07334800	0.00000100
	N8	−3.12429200	0.46490500	0.00000600
	H9	2.46023100	1.94259000	−0.00001000
	H10	3.32336300	−0.68820900	0.00001500
	H11	1.16522900	−2.07783300	0.00000300
	H12	−0.08007400	1.80211500	−0.00000100
Anion	N1	0.67195600	2.05851800	0.00000000
	N2	−0.67221000	2.05845700	0.00000000
	N3	−1.11928500	0.81026500	0.00000000
	C4	0.00000000	0.07854900	0.00000000
	N5	1.11920100	0.81038500	0.00000000
	N6	0.00008100	−1.36159900	0.00000000
	O7	1.08603400	−1.94390800	0.00000000
	O8	−1.08580900	−1.94402600	0.00000000

SCF Done: E(B3LYP, cation) = −390.2793719 a.u.

SCF Done: E(B3LYP, anion) = −462.3675711 a.u.

Table S36: Salt 36

Cartesian coordinates

	Atom	X	Y	Z
Cation	C1	1.06640500	−0.41832800	−0.00003200
	N2	0.03611200	−1.21412900	−0.00000400
	N3	−1.03285300	−0.37700300	0.00003700
	C4	−0.67974900	0.89734900	−0.00005800
	N5	0.65865300	0.89769800	0.00002500
	H6	2.09788600	−0.73449200	−0.00004900
	H7	−1.97156900	−0.76356700	0.00008100
	H8	−1.33192900	1.75728600	−0.00010100
	H9	1.25228900	1.72069000	0.00019900
Anion	N1	0.67195600	2.05851800	0.00000000
	N2	−0.67221000	2.05845700	0.00000000
	N3	−1.11928500	0.81026500	0.00000000
	C4	0.00000000	0.07854900	0.00000000
	N5	1.11920100	0.81038500	0.00000000
	N6	0.00008100	−1.36159900	0.00000000
	O7	1.08603400	−1.94390800	0.00000000
	O8	−1.08580900	−1.94402600	0.00000000

SCF Done: E(B3LYP, cation) = −242.6694066 a.u.

SCF Done: E(B3LYP, anion) = −462.3675711 a.u.

Table S37: Salt 37

Cartesian coordinates

	Atom	X	Y	Z
Cation	C1	−1.34395300	−0.76133900	−0.00002500
	N2	−0.09641400	−1.13546600	0.00009700
	N3	0.61937000	0.02363800	−0.00000100
	C4	−0.17515000	1.07971800	0.00011100
	N5	−1.43290700	0.60853900	−0.00005400
	C6	2.08835000	−0.02286700	−0.00006700
	H7	−2.19345800	−1.42615900	−0.00006900
	H8	0.12266600	2.11627900	0.00017900
	H9	−2.27850900	1.16781500	−0.00027500
	H10	2.46870200	0.99662400	−0.00024600
	H11	2.41742300	−0.55216200	0.89275000
	H12	2.41735100	−0.55244700	−0.89274100
Anion	N1	0.67195600	2.05851800	0.00000000
	N2	−0.67221000	2.05845700	0.00000000
	N3	−1.11928500	0.81026500	0.00000000
	C4	0.00000000	0.07854900	0.00000000
	N5	1.11920100	0.81038500	0.00000000
	N6	0.00008100	−1.36159900	0.00000000
	O7	1.08603400	−1.94390800	0.00000000
	O8	−1.08580900	−1.94402600	0.00000000

SCF Done: E(B3LYP, cation) = −282.0020614 a.u.

SCF Done: E(B3LYP, anion) = −462.3675711 a.u.

Table S38: Salt 38

Cartesian coordinates

	Atom	X	Y	Z
Cation	C1	0.10928300	−0.37050500	−0.00001000
	N2	1.16549400	−1.15216600	−0.00009000
	N3	2.21450300	−0.29340200	0.00006400
	C4	1.85243700	0.97271400	−0.00003500
	N5	0.50786700	0.96531600	0.00002000
	N6	−1.15653700	−0.87371600	0.00003400
	N7	−2.11886300	−0.07468100	0.00000600
	N8	−3.09035500	0.48886000	0.00000100
	H9	3.15781300	−0.66781200	0.00015800
	H10	2.49471700	1.83993100	−0.00001900

	H11	−0.07760900	1.79315200	−0.00011500
Anion	N1	0.67195600	2.05851800	0.00000000
	N2	−0.67221000	2.05845700	0.00000000
	N3	−1.11928500	0.81026500	0.00000000
	C4	0.00000000	0.07854900	0.00000000
	N5	1.11920100	0.81038500	0.00000000
	N6	0.00008100	−1.36159900	0.00000000
	O7	1.08603400	−1.94390800	0.00000000
	O8	−1.08580900	−1.94402600	0.00000000

SCF Done: E(B3LYP, cation) = −406.2900901 a.u.

SCF Done: E(B3LYP, anion) = −462.3675711 a.u.

Table S39: Salt 39

Cartesian coordinates

	Atom	X	Y	Z
Cation	C1	−0.32893900	−0.28721400	0.00001400
	N2	0.79794200	−0.96170600	0.00000800
	N3	1.77356600	−0.01366000	−0.00001900
	C4	1.27830400	1.20574900	0.00001500
	N5	−0.06489500	1.07521800	0.00004500
	N6	−1.54183000	−0.91313700	0.00004800
	N7	−2.57474900	−0.20924800	−0.00001300
	N8	−3.59430700	0.26297300	−0.00004500
	C9	3.18197800	−0.42974100	−0.00002900
	H10	1.82897400	2.13304200	0.00003700
	H11	−0.72595600	1.84313500	−0.00010900
	H12	3.80813200	0.46031100	−0.00018500
	H13	3.36539900	−1.02604800	0.89239800
	H14	3.36530300	−1.02629200	−0.89231100
Anion	N1	0.67195600	2.05851800	0.00000000
	N2	−0.67221000	2.05845700	0.00000000
	N3	−1.11928500	0.81026500	0.00000000
	C4	0.00000000	0.07854900	0.00000000
	N5	1.11920100	0.81038500	0.00000000
	N6	0.00008100	−1.36159900	0.00000000
	O7	1.08603400	−1.94390800	0.00000000
	O8	−1.08580900	−1.94402600	0.00000000

SCF Done: E(B3LYP, cation) = −445.6228493 a.u.

SCF Done: E(B3LYP, anion) = −462.3675711 a.u.

Table S40: Salt 40

Cartesian coordinates

	Atom	X	Y	Z
Cation	C1	1.34509800	−0.32050900	−0.00003300
	N2	0.25046700	−1.04397700	−0.00005000
	N3	−0.76958100	−0.14153200	−0.00008300
	C4	−0.32773300	1.09779800	−0.00012800
	N5	1.02139600	1.02768800	−0.00010900
	N6	2.58684200	−0.89120000	−0.00000800
	N7	3.58509200	−0.14026000	0.00008400
	N8	4.58098200	0.38086700	0.00014900
	C9	−4.62478600	−0.11455300	0.00013300
	C10	−3.20701000	0.47166300	0.00011300
	C11	−2.16740600	−0.64221800	−0.00010900
	H12	−0.91486000	2.00082900	−0.00019000
	H13	1.64705500	1.82428500	0.00005700
	H14	−5.36295900	0.68832100	0.00027200
	H15	−4.80147900	−0.72997800	−0.88518500
	H16	−4.80136900	−0.73018400	0.88533000
	H17	−3.07768000	1.10408400	0.88534900
	H18	−3.07781400	1.10432400	−0.88497100
	H19	−2.25317000	−1.27779800	−0.88345200
	H20	−2.25308400	−1.27807200	0.88304500
Anion	N1	0.67195600	2.05851800	0.00000000
	N2	−0.67221000	2.05845700	0.00000000
	N3	−1.11928500	0.81026500	0.00000000
	C4	0.00000000	0.07854900	0.00000000
	N5	1.11920100	0.81038500	0.00000000
	N6	0.00008100	−1.36159900	0.00000000
	O7	1.08603400	−1.94390800	0.00000000
	O8	−1.08580900	−1.94402600	0.00000000

SCF Done: E(B3LYP, cation) = −524.2783429 a.u.

SCF Done: E(B3LYP, anion) = −462.3675711 a.u.

Table S41: Salt 41

Cartesian coordinates

	Atom	X	Y	Z
Cation	N1	0.72573800	−1.43573300	0.00000000
	C2	1.10022700	−0.18529000	0.00000000
	N3	0.00000000	0.65115400	0.00000000

	C4	−1.08228900	−0.13710400	0.00000000
	N5	−0.61573600	−1.38850300	0.00000000
	N6	0.02662600	2.01061000	0.00000000
	H7	2.12050200	0.16391300	0.00000000
	H8	−2.11338600	0.17676100	0.00000000
	H9	−1.15210300	−2.24869500	0.00000000
	H10	0.92340300	2.46433500	0.00000000
	H11	−0.84244300	2.51536000	0.00000000
Anion	N1	0.67195600	2.05851800	0.00000000
	N2	−0.67221000	2.05845700	0.00000000
	N3	−1.11928500	0.81026500	0.00000000
	C4	0.00000000	0.07854900	0.00000000
	N5	1.11920100	0.81038500	0.00000000
	N6	0.00008100	−1.36159900	0.00000000
	O7	1.08603400	−1.94390800	0.00000000
	O8	−1.08580900	−1.94402600	0.00000000

SCF Done: E(B3LYP, cation) = −297.9953294 a.u.

SCF Done: E(B3LYP, anion) = −462.3675711 a.u.

Table S42: Salt 42

Cartesian coordinates

	Atom	X	Y	Z
Cation	N1	−3.29840800	−0.93880500	0.00023200
	N2	−3.67320100	0.38678800	0.00000300
	C3	−2.59258100	1.10884400	−0.00017900
	N4	−1.48703500	0.28376600	−0.00003400
	C5	−1.99731800	−1.00216200	−0.00007300
	N6	2.20576300	0.49271600	0.00002900
	C7	0.91583700	−0.03675700	0.00000500
	N8	0.94704000	−1.35546600	−0.00004200
	N9	2.27850800	−1.64309600	−0.00003300
	C10	3.03472800	−0.56805900	−0.00003400
	N11	−0.17454100	0.74888300	0.00003200
	C12	2.58513300	1.91320700	0.00008000
	H13	−2.53737700	2.18737300	−0.00028200
	H14	−1.39086800	−1.88959800	−0.00003900
	H15	2.57539500	−2.61215300	−0.00004900
	H16	4.11278100	−0.53272700	−0.00004500
	H17	−0.08978200	1.75336500	0.00004300
	H18	3.67062100	1.98538300	0.00010200
	H19	2.19878900	2.39723600	−0.89828400

	H20	2.19875600	2.39718200	0.89845900
Anion	N1	0.67195600	2.05851800	0.00000000
	N2	−0.67221000	2.05845700	0.00000000
	N3	−1.11928500	0.81026500	0.00000000
	C4	0.00000000	0.07854900	0.00000000
	N5	1.11920100	0.81038500	0.00000000
	N6	0.00008100	−1.36159900	0.00000000
	O7	1.08603400	−1.94390800	0.00000000
	O8	−1.08580900	−1.94402600	0.00000000

SCF Done: E(B3LYP, cation) = −578.4101108 a.u.

SCF Done: E(B3LYP, anion) = −462.3675711 a.u.

Table S43: Salt 43

Cartesian coordinates

	Atom	X	Y	Z
Cation	C1	0.10928300	−0.37050500	−0.00001000
	N2	1.16549400	−1.15216600	−0.00009000
	N3	2.21450300	−0.29340200	0.00006400
	C4	1.85243700	0.97271400	−0.00003500
	N5	0.50786700	0.96531600	0.00002000
	N6	−1.15653700	−0.87371600	0.00003400
	N7	−2.11886300	−0.07468100	0.00000600
	N8	−3.09035500	0.48886000	0.00000100
	H9	3.15781300	−0.66781200	0.00015800
	H10	2.49471700	1.83993100	−0.00001900
	H11	−0.07760900	1.79315200	−0.00011500
Anion	C1	0.58282500	−1.22389200	−0.00000300
	C2	−0.79148400	−1.22195000	0.00001300
	C3	−1.49693900	−0.01630200	0.00001200
	C4	−0.81787400	1.20442100	0.00000600
	C5	0.55605200	1.23619400	−0.00000500
	C6	1.41054200	0.01531000	−0.00003500
	O7	2.63157300	0.02862800	−0.00015200
	N8	1.21373900	−2.54756700	0.00000400
	O9	2.43431100	−2.63285700	0.00013200
	O10	0.46971900	−3.54039400	−0.00014400
	N11	1.15804600	2.57326400	0.00000800
	O12	2.37646400	2.68515000	0.00031400
	O13	0.39260200	3.54965800	−0.00026500
	N14	−2.93812400	−0.03188200	0.00003700
	O15	−3.53876700	1.04886800	0.00003400

	O16	−3.51537200	−1.12530600	0.00004200
	H17	−1.32596500	−2.16021500	0.00002700
	H18	−1.37263400	2.13084200	0.00001500

SCF Done: E(B3LYP, cation) = −406.2900901 a.u.

SCF Done: E(B3LYP, anion) = −920.7359569 a.u.

Table S44: Salt 44

Cartesian coordinates

	Atom	X	Y	Z
Cation	C1	1.06640500	−0.41832800	−0.00003200
	N2	0.03611200	−1.21412900	−0.00000400
	N3	−1.03285300	−0.37700300	0.00003700
	C4	−0.67974900	0.89734900	−0.00005800
	N5	0.65865300	0.89769800	0.00002500
	H6	2.09788600	−0.73449200	−0.00004900
	H7	−1.97156900	−0.76356700	0.00008100
	H8	−1.33192900	1.75728600	−0.00010100
	H9	1.25228900	1.72069000	0.00019900
Anion	C1	0.58282500	−1.22389200	−0.00000300
	C2	−0.79148400	−1.22195000	0.00001300
	C3	−1.49693900	−0.01630200	0.00001200
	C4	−0.81787400	1.20442100	0.00000600
	C5	0.55605200	1.23619400	−0.00000500
	C6	1.41054200	0.01531000	−0.00003500
	O7	2.63157300	0.02862800	−0.00015200
	N8	1.21373900	−2.54756700	0.00000400
	O9	2.43431100	−2.63285700	0.00013200
	O10	0.46971900	−3.54039400	−0.00014400
	N11	1.15804600	2.57326400	0.00000800
	O12	2.37646400	2.68515000	0.00031400
	O13	0.39260200	3.54965800	−0.00026500
	N14	−2.93812400	−0.03188200	0.00003700
	O15	−3.53876700	1.04886800	0.00003400
	O16	−3.51537200	−1.12530600	0.00004200
	H17	−1.32596500	−2.16021500	0.00002700
	H18	−1.37263400	2.13084200	0.00001500

SCF Done: E(B3LYP, cation) = −242.6694066 a.u.

SCF Done: E(B3LYP, anion) = −920.7359569 a.u.

Table S45: Salt 45

Cartesian coordinates

	Atom	X	Y	Z
Cation	C1	−1.34395300	−0.76133900	−0.00002500
	N2	−0.09641400	−1.13546600	0.00009700
	N3	0.61937000	0.02363800	−0.00000100
	C4	−0.17515000	1.07971800	0.00011100
	N5	−1.43290700	0.60853900	−0.00005400
	C6	2.08835000	−0.02286700	−0.00006700
	H7	−2.19345800	−1.42615900	−0.00006900
	H8	0.12266600	2.11627900	0.00017900
	H9	−2.27850900	1.16781500	−0.00027500
	H10	2.46870200	0.99662400	−0.00024600
	H11	2.41742300	−0.55216200	0.89275000
	H12	2.41735100	−0.55244700	−0.89274100
Anion	C1	0.58282500	−1.22389200	−0.00000300
	C2	−0.79148400	−1.22195000	0.00001300
	C3	−1.49693900	−0.01630200	0.00001200
	C4	−0.81787400	1.20442100	0.00000600
	C5	0.55605200	1.23619400	−0.00000500
	C6	1.41054200	0.01531000	−0.00003500
	O7	2.63157300	0.02862800	−0.00015200
	N8	1.21373900	−2.54756700	0.00000400
	O9	2.43431100	−2.63285700	0.00013200
	O10	0.46971900	−3.54039400	−0.00014400
	N11	1.15804600	2.57326400	0.00000800
	O12	2.37646400	2.68515000	0.00031400
	O13	0.39260200	3.54965800	−0.00026500
	N14	−2.93812400	−0.03188200	0.00003700
	O15	−3.53876700	1.04886800	0.00003400
	O16	−3.51537200	−1.12530600	0.00004200
	H17	−1.32596500	−2.16021500	0.00002700
	H18	−1.37263400	2.13084200	0.00001500

SCF Done: E(B3LYP, cation) = −282.0020614 a.u.

SCF Done: E(B3LYP, anion) = −920.7359569 a.u.

Table S46: Salt 46

Cartesian coordinates

	Atom	X	Y	Z
Cation	C1	−0.59451600	1.20187700	−0.00003200
	N2	0.70987100	1.21521700	−0.00001400

	N3	1.06725400	−0.09451300	0.00000000
	C4	0.00077400	−0.88021700	−0.00013000
	N5	−1.08025000	−0.08242400	−0.00002500
	C6	−2.49413600	−0.50489600	0.00008000
	C7	2.48650600	−0.47089700	0.00007100
	H8	−1.22032700	2.08055400	−0.00001100
	H9	−0.00234600	−1.95863900	−0.00020400
	H10	−3.11391800	0.38900000	0.00000800
	H11	−2.70184400	−1.08933900	−0.89524100
	H12	−2.70178700	−1.08916200	0.89553200
	H13	2.55902600	−1.55669300	0.00020400
	H14	2.95566400	−0.05954800	−0.89231300
	H15	2.95564100	−0.05933200	0.89236600
Anion	C1	0.58282500	−1.22389200	−0.00000300
	C2	−0.79148400	−1.22195000	0.00001300
	C3	−1.49693900	−0.01630200	0.00001200
	C4	−0.81787400	1.20442100	0.00000600
	C5	0.55605200	1.23619400	−0.00000500
	C6	1.41054200	0.01531000	−0.00003500
	O7	2.63157300	0.02862800	−0.00015200
	N8	1.21373900	−2.54756700	0.00000400
	O9	2.43431100	−2.63285700	0.00013200
	O10	0.46971900	−3.54039400	−0.00014400
	N11	1.15804600	2.57326400	0.00000800
	O12	2.37646400	2.68515000	0.00031400
	O13	0.39260200	3.54965800	−0.00026500
	N14	−2.93812400	−0.03188200	0.00003700
	O15	−3.53876700	1.04886800	0.00003400
	O16	−3.51537200	−1.12530600	0.00004200
	H17	−1.32596500	−2.16021500	0.00002700
	H18	−1.37263400	2.13084200	0.00001500

SCF Done: E(B3LYP, cation) = −321.329351 a.u.

SCF Done: E(B3LYP, anion) = −920.7359569 a.u.

Table S47: Salt 47

Cartesian coordinates

	Atom	X	Y	Z
Cation	C1	0.18477700	−0.48806600	−0.00017100
	N2	−0.95937800	−1.14105000	−0.00018500
	N3	−1.91196800	−0.18023500	0.00005300
	C4	−1.38445400	1.02649200	0.00005300

	N5	−0.04134400	0.88642500	−0.00005800
	C6	0.89524300	2.03075200	−0.00012200
	C7	−3.32873300	−0.56230300	0.00021500
	N8	1.34903900	−1.20352600	−0.00035600
	N9	2.47345600	−0.67220400	0.00009700
	N10	3.56526000	−0.39981600	0.00040300
	H11	−1.91807500	1.96378500	0.00016800
	H12	1.91877100	1.67476400	−0.00053300
	H13	0.73147200	2.62963000	−0.89531300
	H14	0.73204600	2.62928600	0.89540500
	H15	−3.93391500	0.34219600	0.00043500
	H16	−3.52851500	−1.15391500	−0.89190000
	H17	−3.52824900	−1.15417100	0.89221900
Anion	C1	0.58282500	−1.22389200	−0.00000300
	C2	−0.79148400	−1.22195000	0.00001300
	C3	−1.49693900	−0.01630200	0.00001200
	C4	−0.81787400	1.20442100	0.00000600
	C5	0.55605200	1.23619400	−0.00000500
	C6	1.41054200	0.01531000	−0.00003500
	O7	2.63157300	0.02862800	−0.00015200
	N8	1.21373900	−2.54756700	0.00000400
	O9	2.43431100	−2.63285700	0.00013200
	O10	0.46971900	−3.54039400	−0.00014400
	N11	1.15804600	2.57326400	0.00000800
	O12	2.37646400	2.68515000	0.00031400
	O13	0.39260200	3.54965800	−0.00026500
	N14	−2.93812400	−0.03188200	0.00003700
	O15	−3.53876700	1.04886800	0.00003400
	O16	−3.51537200	−1.12530600	0.00004200
	H17	−1.32596500	−2.16021500	0.00002700
	H18	−1.37263400	2.13084200	0.00001500

SCF Done: E(B3LYP, cation) = −484.9450554 a.u.

SCF Done: E(B3LYP, anion) = −920.7359569 a.u.

Table S48: Salt 48

Cartesian coordinates

	Atom	X	Y	Z
Cation	C1	−0.67468300	0.00935200	−0.00001100
	N2	0.12778500	−1.06305900	0.00037200
	N3	1.46009600	−0.72163800	−0.00059300
	C4	1.46120000	0.56763800	0.00028800

	N5	0.16620400	1.07016600	−0.00002300
	N6	−2.00636100	0.03613400	−0.00011800
	H7	−0.13122800	−2.04067600	0.00063500
	H8	2.34086100	1.19145000	0.00034700
	H9	−0.09999700	2.04618100	0.00021400
	H10	−2.54623400	−0.81626400	0.00015500
	H11	−2.51656700	0.90615400	−0.00048200
Anion	C1	0.58282500	−1.22389200	−0.00000300
	C2	−0.79148400	−1.22195000	0.00001300
	C3	−1.49693900	−0.01630200	0.00001200
	C4	−0.81787400	1.20442100	0.00000600
	C5	0.55605200	1.23619400	−0.00000500
	C6	1.41054200	0.01531000	−0.00003500
	O7	2.63157300	0.02862800	−0.00015200
	N8	1.21373900	−2.54756700	0.00000400
	O9	2.43431100	−2.63285700	0.00013200
	O10	0.46971900	−3.54039400	−0.00014400
	N11	1.15804600	2.57326400	0.00000800
	O12	2.37646400	2.68515000	0.00031400
	O13	0.39260200	3.54965800	−0.00026500
	N14	−2.93812400	−0.03188200	0.00003700
	O15	−3.53876700	1.04886800	0.00003400
	O16	−3.51537200	−1.12530600	0.00004200
	H17	−1.32596500	−2.16021500	0.00002700
	H18	−1.37263400	2.13084200	0.00001500

SCF Done: E(B3LYP, cation) = −298.0636709 a.u.

SCF Done: E(B3LYP, anion) = −920.7359569 a.u.

Table S49: Salt 49

Cartesian coordinates

	Atom	X	Y	Z
Cation	N1	0.72573800	−1.43573300	0.00000000
	C2	1.10022700	−0.18529000	0.00000000
	N3	0.00000000	0.65115400	0.00000000
	C4	−1.08228900	−0.13710400	0.00000000
	N5	−0.61573600	−1.38850300	0.00000000
	N6	0.02662600	2.01061000	0.00000000
	H7	2.12050200	0.16391300	0.00000000
	H8	−2.11338600	0.17676100	0.00000000
	H9	−1.15210300	−2.24869500	0.00000000
	H10	0.92340300	2.46433500	0.00000000

	H11	−0.84244300	2.51536000	0.00000000
Anion	C1	0.58282500	−1.22389200	−0.00000300
	C2	−0.79148400	−1.22195000	0.00001300
	C3	−1.49693900	−0.01630200	0.00001200
	C4	−0.81787400	1.20442100	0.00000600
	C5	0.55605200	1.23619400	−0.00000500
	C6	1.41054200	0.01531000	−0.00003500
	O7	2.63157300	0.02862800	−0.00015200
	N8	1.21373900	−2.54756700	0.00000400
	O9	2.43431100	−2.63285700	0.00013200
	O10	0.46971900	−3.54039400	−0.00014400
	N11	1.15804600	2.57326400	0.00000800
	O12	2.37646400	2.68515000	0.00031400
	O13	0.39260200	3.54965800	−0.00026500
	N14	−2.93812400	−0.03188200	0.00003700
	O15	−3.53876700	1.04886800	0.00003400
	O16	−3.51537200	−1.12530600	0.00004200
	H17	−1.32596500	−2.16021500	0.00002700
	H18	−1.37263400	2.13084200	0.00001500

SCF Done: E(B3LYP, cation) = −297.9953294 a.u.

SCF Done: E(B3LYP, anion) = −920.7359569 a.u.

Table S50: Salt 50

Cartesian coordinates

	Atom	X	Y	Z
Cation	N1	−1.47534100	−0.62785300	0.00003000
	N2	−1.47587000	0.62752200	−0.00006500
	N3	−0.17047500	1.05257900	0.00004300
	C4	0.66687500	0.00044000	0.00000300
	N5	−0.17063300	−1.05243600	−0.00001100
	N6	1.99178000	−0.00011800	−0.00001900
	H7	0.03292400	2.04532000	0.00005500
	H8	0.03343500	−2.04505300	0.00011200
	H9	2.51851900	0.86180900	0.00006500
	H10	2.51764600	−0.86257100	−0.00009200
Anion	C1	0.58282500	−1.22389200	−0.00000300
	C2	−0.79148400	−1.22195000	0.00001300
	C3	−1.49693900	−0.01630200	0.00001200
	C4	−0.81787400	1.20442100	0.00000600
	C5	0.55605200	1.23619400	−0.00000500
	C6	1.41054200	0.01531000	−0.00003500

	O7	2.63157300	0.02862800	−0.00015200
	N8	1.21373900	−2.54756700	0.00000400
	O9	2.43431100	−2.63285700	0.00013200
	O10	0.46971900	−3.54039400	−0.00014400
	N11	1.15804600	2.57326400	0.00000800
	O12	2.37646400	2.68515000	0.00031400
	O13	0.39260200	3.54965800	−0.00026500
	N14	−2.93812400	−0.03188200	0.00003700
	O15	−3.53876700	1.04886800	0.00003400
	O16	−3.51537200	−1.12530600	0.00004200
	H17	−1.32596500	−2.16021500	0.00002700
	H18	−1.37263400	2.13084200	0.00001500

SCF Done: E(B3LYP, cation) = −314.0552154 a.u.

SCF Done: E(B3LYP, anion) = −920.7359569 a.u.

Table S51: Salt 51

Cartesian coordinates

	Atom	X	Y	Z
Cation	C1	1.64787200	1.13566200	−0.06142100
	C2	2.86433100	0.94984000	−0.63606800
	N3	3.16446100	−0.39148900	−0.49628800
	C4	2.17674400	−1.01703300	0.13675800
	N5	1.22537500	−0.10623900	0.41089400
	C6	0.00916300	−0.37648500	1.19147100
	N7	−1.22880200	−0.16276700	0.43452600
	C8	−1.83487400	−1.06326300	−0.36118800
	N9	−2.95332600	−0.50874200	−0.81761500
	C10	−3.09431100	0.76884800	−0.31097100
	C11	−2.01279400	0.99019700	0.48007900
	H12	1.06497600	2.03401400	0.05321800
	H13	3.53221200	1.64678700	−1.11641500
	H14	4.01885700	−0.83999300	−0.81505800
	H15	2.15645500	−2.06343700	0.39998000
	H16	0.04690200	−1.40701800	1.54124300
	H17	−0.01128500	0.28186300	2.05938100
	H18	−1.48966000	−2.06121100	−0.58203800
	H19	−3.61538100	−0.97269300	−1.43358900
	H20	−3.94084200	1.39511600	−0.54270700
	H21	−1.75297700	1.84464100	1.08340200
Anion	C1	0.58282500	−1.22389200	−0.00000300
	C2	−0.79148400	−1.22195000	0.00001300

	C3	−1.49693900	−0.01630200	0.00001200
	C4	−0.81787400	1.20442100	0.00000600
	C5	0.55605200	1.23619400	−0.00000500
	C6	1.41054200	0.01531000	−0.00003500
	O7	2.63157300	0.02862800	−0.00015200
	N8	1.21373900	−2.54756700	0.00000400
	O9	2.43431100	−2.63285700	0.00013200
	O10	0.46971900	−3.54039400	−0.00014400
	N11	1.15804600	2.57326400	0.00000800
	O12	2.37646400	2.68515000	0.00031400
	O13	0.39260200	3.54965800	−0.00026500
	N14	−2.93812400	−0.03188200	0.00003700
	O15	−3.53876700	1.04886800	0.00003400
	O16	−3.51537200	−1.12530600	0.00004200
	H17	−1.32596500	−2.16021500	0.00002700
	H18	−1.37263400	2.13084200	0.00001500

SCF Done: E(B3LYP, cation) = −491.3068027 a.u.

SCF Done: E(B3LYP, anion) = −920.7359569 a.u.

Table S52: Salt 52

Cartesian coordinates

	Atom	X	Y	Z
Cation	C1	−1.62853500	−1.19121100	−0.61498300
	C2	−2.82122300	−0.68378700	−1.02058800
	N3	−3.12051700	0.38479600	−0.19242000
	C4	−2.13842900	0.52984100	0.69082700
	N5	−1.20365400	−0.41489800	0.45708200
	C6	−0.01379400	−0.64351300	1.28615800
	N7	1.24769400	−0.43448400	0.57262000
	C8	1.89795200	0.73998100	0.43106700
	N9	3.03072800	0.52105500	−0.22763200
	C10	3.12680200	−0.82940700	−0.51866400
	C11	2.01764300	−1.43138600	−0.01746600
	C12	−4.34671100	1.20972000	−0.27251100
	C13	4.04645600	1.54044700	−0.57283500
	H14	−1.06502300	−2.02810800	−0.99183000
	H15	−3.47993600	−0.99520000	−1.81525500
	H16	−2.10666500	1.26737200	1.47735200
	H17	−0.06002500	0.02617800	2.14353100
	H18	−0.02476900	−1.67015700	1.65085200
	H19	1.56558900	1.69613000	0.80278800

	H20	3.97333600	−1.24466100	−1.04138600
	H21	1.72994000	−2.46995600	−0.00827900
	H22	−5.21426400	0.56913800	−0.11828700
	H23	−4.31095600	1.97227700	0.50258800
	H24	−4.39404600	1.68159900	−1.25310200
	H25	4.17199900	1.56363900	−1.65460100
	H26	3.70719600	2.51187000	−0.21968800
	H27	4.98690900	1.28047200	−0.08826000
Anion	C1	0.58282500	−1.22389200	−0.00000300
	C2	−0.79148400	−1.22195000	0.00001300
	C3	−1.49693900	−0.01630200	0.00001200
	C4	−0.81787400	1.20442100	0.00000600
	C5	0.55605200	1.23619400	−0.00000500
	C6	1.41054200	0.01531000	−0.00003500
	O7	2.63157300	0.02862800	−0.00015200
	N8	1.21373900	−2.54756700	0.00000400
	O9	2.43431100	−2.63285700	0.00013200
	O10	0.46971900	−3.54039400	−0.00014400
	N11	1.15804600	2.57326400	0.00000800
	O12	2.37646400	2.68515000	0.00031400
	O13	0.39260200	3.54965800	−0.00026500
	N14	−2.93812400	−0.03188200	0.00003700
	O15	−3.53876700	1.04886800	0.00003400
	O16	−3.51537200	−1.12530600	0.00004200
	H17	−1.32596500	−2.16021500	0.00002700
	H18	−1.37263400	2.13084200	0.00001500

SCF Done: E(B3LYP, cation) = −569.967895 a.u.

SCF Done: E(B3LYP, anion) = −920.7359569 a.u.

Table S53: Salt 53

Cartesian coordinates

	Atom	X	Y	Z
Cation	N1	−1.37381300	−1.31820500	−0.06154200
	C2	−2.53355700	−1.16897500	−0.63609400
	N3	−3.09988100	0.04683100	−0.31343000
	C4	−2.23229500	0.65978900	0.49349800
	N5	−1.19498500	−0.16706400	0.64411900
	C6	0.00005100	−0.00357900	1.46664200
	N7	1.19494100	0.16381000	0.64461500
	C8	2.23280800	−0.66175700	0.49088200
	N9	3.10004400	−0.04510800	−0.31361700

	Atom	X	Y	Z
	C10	2.53293500	1.17154500	−0.63162300
	N11	1.37305900	1.31779300	−0.05655400
	C12	−4.41959000	0.55459300	−0.76432300
	C13	4.42006500	−0.55035400	−0.76643900
	H14	−3.00289400	−1.89933500	−1.27900000
	H15	−2.35011000	1.63604800	0.94005500
	H16	−0.12016200	0.88030000	2.09162300
	H17	0.12040900	−0.89048000	2.08729600
	H18	2.35127400	−1.63965400	0.93367400
	H19	3.00181000	1.90468800	−1.27169200
	H20	−4.42754800	0.59760800	−1.85251500
	H21	−5.19739500	−0.11587900	−0.40091200
	H22	−4.56886400	1.55081800	−0.35386600
	H23	4.42817900	−0.58885500	−1.85479700
	H24	5.19751100	0.11897200	−0.40015400
	H25	4.56973100	−1.54820200	−0.36009800
Anion	C1	0.58282500	−1.22389200	−0.00000300
	C2	−0.79148400	−1.22195000	0.00001300
	C3	−1.49693900	−0.01630200	0.00001200
	C4	−0.81787400	1.20442100	0.00000600
	C5	0.55605200	1.23619400	−0.00000500
	C6	1.41054200	0.01531000	−0.00003500
	O7	2.63157300	0.02862800	−0.00015200
	N8	1.21373900	−2.54756700	0.00000400
	O9	2.43431100	−2.63285700	0.00013200
	O10	0.46971900	−3.54039400	−0.00014400
	N11	1.15804600	2.57326400	0.00000800
	O12	2.37646400	2.68515000	0.00031400
	O13	0.39260200	3.54965800	−0.00026500
	N14	−2.93812400	−0.03188200	0.00003700
	O15	−3.53876700	1.04886800	0.00003400
	O16	−3.51537200	−1.12530600	0.00004200
	H17	−1.32596500	−2.16021500	0.00002700
	H18	−1.37263400	2.13084200	0.00001500

SCF Done: E(B3LYP, cation) = −602.0167882 a.u.

SCF Done: E(B3LYP, anion) = −920.7359569 a.u.

Table S54: Salt 54

Cartesian coordinates

	Atom	X	Y	Z
Cation	C1	0.78681300	1.15357500	−0.00018800

	C2	2.14493400	1.26663800	−0.00005400
	N3	2.66044300	−0.01289200	0.00004400
	C4	1.63922300	−0.87746600	0.00001500
	N5	0.48930400	−0.19414000	−0.00013000
	C6	−4.54602400	0.43180200	0.00023600
	C7	−3.36875500	−0.54640400	−0.00002800
	C8	−2.01005100	0.17217000	0.00003700
	C9	−0.86201500	−0.83065100	−0.00025200
	C10	4.08826000	−0.37508200	0.00022500
	H11	0.02711100	1.91396500	−0.00030400
	H12	2.77586200	2.13896300	−0.00003800
	H13	1.72872000	−1.95159400	0.00008200
	H14	−5.49569200	−0.10660600	0.00017800
	H15	−4.52983400	1.07464300	0.88491500
	H16	−4.52993400	1.07499800	−0.88418700
	H17	−3.43357000	−1.19878700	−0.87800800
	H18	−3.43346700	−1.19914200	0.87769700
	H19	−1.94441900	0.81567700	0.88409700
	H20	−1.94454600	0.81605100	−0.88376000
	H21	−0.91103200	−1.46733700	−0.88607000
	H22	−0.91097100	−1.46776900	0.88525800
	H23	4.67311000	0.54169400	−0.00006200
	H24	4.32302700	−0.95121500	0.89461200
	H25	4.32309800	−0.95180700	−0.89376100
Anion	N1	0.66551300	3.68113200	0.00000000
	N2	−0.66551300	3.83151700	0.00000000
	N3	−1.24462900	2.63331700	0.00000000
	C4	−0.22031300	1.75474100	0.00000000
	N5	0.97206800	2.38478800	0.00000000
	C6	0.22031300	−1.75474100	0.00000000
	N7	1.24462900	−2.63331700	0.00000000
	N8	0.66551300	−3.83151700	0.00000000
	N9	−0.66551300	−3.68113200	0.00000000
	N10	−0.97206800	−2.38478800	0.00000000
	N11	−0.50128100	0.38137300	0.00000000
	N12	0.50128100	−0.38137300	0.00000000

SCF Done: E(B3LYP, cation) = −423.2848953 a.u.

SCF Done: E(B3LYP, anion) = −623.7876469 a.u.

Table S55: Salt 55

Cartesian coordinates

	Atom	X	Y	Z
Cation	C1	−0.37645700	−0.26725600	0.00013500
	C2	0.72052100	−1.07657400	0.00015500
	N3	1.81260500	−0.24567800	0.00001000
	C4	1.38279000	1.02472300	−0.00013800
	N5	0.04834900	1.05155300	−0.00009100
	N6	−1.76672600	−0.69539800	0.00000400
	O7	−1.94271500	−1.89826000	−0.00027700
	O8	−2.61598000	0.18102900	0.00020900
	C9	−0.75882600	2.29636600	−0.00001800
	C10	3.22317100	−0.67991200	0.00003500
	H11	0.78492900	−2.15207200	0.00026700
	H12	2.01767400	1.89617200	−0.00032800
	H13	−1.38498700	2.32179800	−0.88835600
	H14	−1.38471900	2.32182800	0.88850400
	H15	−0.06349400	3.13332300	−0.00014200
	H16	3.41642000	−1.27071100	0.89449900
	H17	3.41618700	−1.27155400	−0.89392100
	H18	3.86075600	0.20164600	−0.00045100
Anion	N1	0.66551300	3.68113200	0.00000000
	N2	−0.66551300	3.83151700	0.00000000
	N3	−1.24462900	2.63331700	0.00000000
	C4	−0.22031300	1.75474100	0.00000000
	N5	0.97206800	2.38478800	0.00000000
	C6	0.22031300	−1.75474100	0.00000000
	N7	1.24462900	−2.63331700	0.00000000
	N8	0.66551300	−3.83151700	0.00000000
	N9	−0.66551300	−3.68113200	0.00000000
	N10	−0.97206800	−2.38478800	0.00000000
	N11	−0.50128100	0.38137300	0.00000000
	N12	0.50128100	−0.38137300	0.00000000

SCF Done: E(B3LYP, cation) = −509.839047 a.u.

SCF Done: E(B3LYP, anion) = −623.7876469 a.u.

Table S56: Salt 56

Cartesian coordinates

	Atom	X	Y	Z
Cation	C1	−0.59451600	1.20187700	−0.00003200
	N2	0.70987100	1.21521700	−0.00001400
	N3	1.06725400	−0.09451300	0.00000000
	C4	0.00077400	−0.88021700	−0.00013000

	N5	−1.08025000	−0.08242400	−0.00002500
	C6	−2.49413600	−0.50489600	0.00008000
	C7	2.48650600	−0.47089700	0.00007100
	H8	−1.22032700	2.08055400	−0.00001100
	H9	−0.00234600	−1.95863900	−0.00020400
	H10	−3.11391800	0.38900000	0.00000800
	H11	−2.70184400	−1.08933900	−0.89524100
	H12	−2.70178700	−1.08916200	0.89553200
	H13	2.55902600	−1.55669300	0.00020400
	H14	2.95566400	−0.05954800	−0.89231300
	H15	2.95564100	−0.05933200	0.89236600
Anion	N1	0.66551300	3.68113200	0.00000000
	N2	−0.66551300	3.83151700	0.00000000
	N3	−1.24462900	2.63331700	0.00000000
	C4	−0.22031300	1.75474100	0.00000000
	N5	0.97206800	2.38478800	0.00000000
	C6	0.22031300	−1.75474100	0.00000000
	N7	1.24462900	−2.63331700	0.00000000
	N8	0.66551300	−3.83151700	0.00000000
	N9	−0.66551300	−3.68113200	0.00000000
	N10	−0.97206800	−2.38478800	0.00000000
	N11	−0.50128100	0.38137300	0.00000000
	N12	0.50128100	−0.38137300	0.00000000

SCF Done: E(B3LYP, cation) = −321.329351 a.u.

SCF Done: E(B3LYP, anion) = −623.7876469 a.u.

Table S57: Salt 57

Cartesian coordinates

	Atom	X	Y	Z
Cation	C1	0.18477700	−0.48806600	−0.00017100
	N2	−0.95937800	−1.14105000	−0.00018500
	N3	−1.91196800	−0.18023500	0.00005300
	C4	−1.38445400	1.02649200	0.00005300
	N5	−0.04134400	0.88642500	−0.00005800
	C6	0.89524300	2.03075200	−0.00012200
	C7	−3.32873300	−0.56230300	0.00021500
	N8	1.34903900	−1.20352600	−0.00035600
	N9	2.47345600	−0.67220400	0.00009700
	N10	3.56526000	−0.39981600	0.00040300
	H11	−1.91807500	1.96378500	0.00016800
	H12	1.91877100	1.67476400	−0.00053300

	H13	0.73147200	2.62963000	−0.89531300
	H14	0.73204600	2.62928600	0.89540500
	H15	−3.93391500	0.34219600	0.00043500
	H16	−3.52851500	−1.15391500	−0.89190000
	H17	−3.52824900	−1.15417100	0.89221900
Anion	N1	0.66551300	3.68113200	0.00000000
	N2	−0.66551300	3.83151700	0.00000000
	N3	−1.24462900	2.63331700	0.00000000
	C4	−0.22031300	1.75474100	0.00000000
	N5	0.97206800	2.38478800	0.00000000
	C6	0.22031300	−1.75474100	0.00000000
	N7	1.24462900	−2.63331700	0.00000000
	N8	0.66551300	−3.83151700	0.00000000
	N9	−0.66551300	−3.68113200	0.00000000
	N10	−0.97206800	−2.38478800	0.00000000
	N11	−0.50128100	0.38137300	0.00000000
	N12	0.50128100	−0.38137300	0.00000000

SCF Done: E(B3LYP, cation) = −484.9450554 a.u.

SCF Done: E(B3LYP, anion) = −623.7876469 a.u.

Table S58: Salt 58

Cartesian coordinates

	Atom	X	Y	Z
Cation	N1	−0.67062800	1.21160500	0.00003500
	C2	0.63691100	1.18917700	−0.00001800
	N3	1.09525400	−0.10727600	−0.00002800
	C4	0.00744600	−0.89435100	0.00000600
	N5	−1.04914100	−0.07890200	0.00001700
	N6	2.39810400	−0.50640700	−0.00006700
	C7	−2.47273800	−0.43828300	0.00006100
	H8	1.27858000	2.05575300	−0.00003600
	H9	−0.00304800	−1.97160800	0.00000600
	H10	3.10846400	0.20392000	−0.00009900
	H11	2.60559400	−1.48918100	−0.00007600
	H12	−2.55730100	−1.52323800	−0.00009900
	H13	−2.93850600	−0.02415500	0.89301100
	H14	−2.93861800	−0.02388600	−0.89270400
Anion	N1	0.66551300	3.68113200	0.00000000
	N2	−0.66551300	3.83151700	0.00000000
	N3	−1.24462900	2.63331700	0.00000000
	C4	−0.22031300	1.75474100	0.00000000

	N5	0.97206800	2.38478800	0.00000000
	C6	0.22031300	−1.75474100	0.00000000
	N7	1.24462900	−2.63331700	0.00000000
	N8	0.66551300	−3.83151700	0.00000000
	N9	−0.66551300	−3.68113200	0.00000000
	N10	−0.97206800	−2.38478800	0.00000000
	N11	−0.50128100	0.38137300	0.00000000
	N12	0.50128100	−0.38137300	0.00000000

SCF Done: E(B3LYP, cation) = −337.3261717 a.u.

SCF Done: E(B3LYP, anion) = −623.7876469 a.u.

Table S59: Salt 59

Cartesian coordinates

	Atom	X	Y	Z
Cation	C1	0.59401000	1.20341000	−0.00008700
	N2	−0.70712400	1.21582900	0.00017200
	N3	−1.06118300	−0.10562400	0.00022600
	C4	−0.00316900	−0.90017700	−0.00023200
	N5	1.07066800	−0.09532700	−0.00063300
	N6	2.38079800	−0.58790700	0.00029200
	N7	−2.37221900	−0.56044600	0.00014800
	H8	1.22888500	2.07678200	−0.00042500
	H9	0.00090700	−1.97858500	−0.00050200
	H10	2.86658100	−0.28191500	0.83865900
	H11	2.86833300	−0.28067400	−0.83657800
	H12	−2.84360300	−0.20986000	0.83125400
	H13	−2.84272500	−0.21082700	−0.83192800
Anion	N1	0.66551300	3.68113200	0.00000000
	N2	−0.66551300	3.83151700	0.00000000
	N3	−1.24462900	2.63331700	0.00000000
	C4	−0.22031300	1.75474100	0.00000000
	N5	0.97206800	2.38478800	0.00000000
	C6	0.22031300	−1.75474100	0.00000000
	N7	1.24462900	−2.63331700	0.00000000
	N8	0.66551300	−3.83151700	0.00000000
	N9	−0.66551300	−3.68113200	0.00000000
	N10	−0.97206800	−2.38478800	0.00000000
	N11	−0.50128100	0.38137300	0.00000000
	N12	0.50128100	−0.38137300	0.00000000

SCF Done: E(B3LYP, cation) = −353.3536473 a.u.

SCF Done: E(B3LYP, anion) = −623.7876469 a.u.

Table S60: Salt 60

Cartesian coordinates

	Atom	X	Y	Z
Cation	N1	0.63151800	−1.53175700	0.00017900
	N2	−0.63750600	−1.53002600	−0.00069800
	N3	−1.05571500	−0.23777700	0.00005900
	C4	0.00062000	0.58293300	−0.00017600
	N5	1.05612100	−0.24001700	0.00002300
	C6	2.49454500	0.06414800	0.00026300
	C7	0.00297500	2.06623300	−0.00026200
	C8	−2.49346800	0.06453600	0.00041600
	H9	3.01255800	−0.89198200	0.00052200
	H10	2.75264700	0.62484100	−0.89737600
	H11	2.75226100	0.62510600	0.89784600
	H12	1.02206200	2.44947700	−0.00006000
	H13	−0.50977200	2.44820700	−0.88677800
	H14	−0.51016600	2.44831600	0.88597600
	H15	−2.62545000	1.14379800	0.00106400
	H16	−2.94165000	−0.36839200	−0.89268300
	H17	−2.94145100	−0.36943600	0.89310700
Anion	N1	0.66551300	3.68113200	0.00000000
	N2	−0.66551300	3.83151700	0.00000000
	N3	−1.24462900	2.63331700	0.00000000
	C4	−0.22031300	1.75474100	0.00000000
	N5	0.97206800	2.38478800	0.00000000
	C6	0.22031300	−1.75474100	0.00000000
	N7	1.24462900	−2.63331700	0.00000000
	N8	0.66551300	−3.83151700	0.00000000
	N9	−0.66551300	−3.68113200	0.00000000
	N10	−0.97206800	−2.38478800	0.00000000
	N11	−0.50128100	0.38137300	0.00000000
	N12	0.50128100	−0.38137300	0.00000000

SCF Done: E(B3LYP, cation) = −376.6673328 a.u.

SCF Done: E(B3LYP, anion) = −623.7876469 a.u.

Table S61: Salt 61

Cartesian coordinates

	Atom	X	Y	Z

Cation	N1	0.72573800	−1.43573300	0.00000000
	C2	1.10022700	−0.18529000	0.00000000
	N3	0.00000000	0.65115400	0.00000000
	C4	−1.08228900	−0.13710400	0.00000000
	N5	−0.61573600	−1.38850300	0.00000000
	N6	0.02662600	2.01061000	0.00000000
	H7	2.12050200	0.16391300	0.00000000
	H8	−2.11338600	0.17676100	0.00000000
	H9	−1.15210300	−2.24869500	0.00000000
	H10	0.92340300	2.46433500	0.00000000
	H11	−0.84244300	2.51536000	0.00000000
Anion	N1	0.00000000	−3.01602700	−0.99279100
	N2	0.00000000	−3.41216300	0.25817100
	N3	0.00000000	−2.33510900	1.07581300
	C4	0.00000000	−1.28379000	0.23118600
	N5	0.00000000	−1.65522400	−1.05079800
	C6	0.00000000	1.28379000	0.23118600
	N7	0.00000000	2.33510900	1.07581300
	N8	0.00000000	3.41216300	0.25817100
	N9	0.00000000	3.01602700	−0.99279100
	N10	0.00000000	1.65522400	−1.05079800
	N11	0.00000000	0.00000000	0.76918300
	H12	0.00000000	0.00000000	1.77595000

SCF Done: E(B3LYP, cation) = −297.9953294 a.u.

SCF Done: E(B3LYP, anion) = −569.6559441 a.u.

Table S62: Salt 62

Cartesian coordinates

	Atom	X	Y	Z
Cation	N1	0.72573800	−1.43573300	0.00000000
	C2	1.10022700	−0.18529000	0.00000000
	N3	0.00000000	0.65115400	0.00000000
	C4	−1.08228900	−0.13710400	0.00000000
	N5	−0.61573600	−1.38850300	0.00000000
	N6	0.02662600	2.01061000	0.00000000
	H7	2.12050200	0.16391300	0.00000000
	H8	−2.11338600	0.17676100	0.00000000
	H9	−1.15210300	−2.24869500	0.00000000
	H10	0.92340300	2.46433500	0.00000000
	H11	−0.84244300	2.51536000	0.00000000
Anion	N1	−0.36965900	2.81889800	0.00000000

N2	0.94888300	2.68000500	0.00000000
N3	1.26302000	1.37463600	0.00000000
C4	0.07711200	0.73207500	0.00000000
N5	−0.94888300	1.60762500	0.00000000
C6	−0.07711200	−0.73207500	0.00000000
N7	−1.26302000	−1.37463600	0.00000000
N8	−0.94888300	−2.68000500	0.00000000
N9	0.36965900	−2.81889800	0.00000000
N10	0.94888300	−1.60762500	0.00000000

SCF Done: E(B3LYP, cation) = −297.9953294 a.u.

SCF Done: E(B3LYP, anion) = −514.2825578 a.u.

Table S63: Salt 63

Cartesian coordinates

	Atom	X	Y	Z
Cation	N1	−2.30865200	0.81588100	0.00000000
	C2	−1.21694900	0.03718400	0.00000000
	N3	0.00000000	0.64962600	0.00000000
	N4	−1.29726400	−1.27578000	0.00000000
	C5	1.27171000	0.00451000	0.00000000
	N6	2.30605800	0.88525700	0.00000000
	N7	1.30701300	−1.27093700	0.00000000
	H8	−3.23217400	0.41098700	0.00000000
	H9	−2.25308100	1.82198600	0.00000000
	H10	−0.00498400	1.65842700	0.00000000
	H11	−0.38525300	−1.77463900	0.00000000
	H12	−2.18726700	−1.75083000	0.00000000
	H13	3.25154100	0.53751900	0.00000000
	H14	2.19157500	1.88552200	0.00000000
	H15	2.24099900	−1.66747000	0.00000000
Anion	N1	−0.65342100	−1.43332300	0.00000000
	N2	0.65342800	−1.43330900	0.00000000
	N3	1.11552800	−0.15197500	0.00000000
	C4	0.00000000	0.57947300	0.00000000
	N5	−1.11552100	−0.15199200	0.00000000
	N6	−0.00001000	1.96951600	0.00000000
	H7	0.87011600	2.46537900	0.00000000
	H8	−0.87014500	2.46536400	0.00000000

SCF Done: E(B3LYP, cation) = −354.7051979 a.u.

SCF Done: E(B3LYP, anion) = −313.1609514 a.u.

Table S64: Salt 64

Cartesian coordinates

	Atom	X	Y	Z
Cation	N1	0.72573800	−1.43573300	0.00000000
	C2	1.10022700	−0.18529000	0.00000000
	N3	0.00000000	0.65115400	0.00000000
	C4	−1.08228900	−0.13710400	0.00000000
	N5	−0.61573600	−1.38850300	0.00000000
	N6	0.02662600	2.01061000	0.00000000
	H7	2.12050200	0.16391300	0.00000000
	H8	−2.11338600	0.17676100	0.00000000
	H9	−1.15210300	−2.24869500	0.00000000
	H10	0.92340300	2.46433500	0.00000000
	H11	−0.84244300	2.51536000	0.00000000
Anion	N1	−0.65342100	−1.43332300	0.00000000
	N2	0.65342800	−1.43330900	0.00000000
	N3	1.11552800	−0.15197500	0.00000000
	C4	0.00000000	0.57947300	0.00000000
	N5	−1.11552100	−0.15199200	0.00000000
	N6	−0.00001000	1.96951600	0.00000000
	H7	0.87011600	2.46537900	0.00000000
	H8	−0.87014500	2.46536400	0.00000000

SCF Done: E(B3LYP, cation) = −297.9953294 a.u.

SCF Done: E(B3LYP, anion) = −313.1609514 a.u.

Table S65: Salt 65

Cartesian coordinates

	Atom	X	Y	Z
Cation	N1	0.01505600	1.33482600	−0.00016400
	C2	0.00005600	0.00000600	0.00002800
	N3	1.14853800	−0.68050900	0.00012700
	N4	−1.16365100	−0.65433800	−0.00009900
	H5	0.86148500	1.85114100	−0.18670200
	H6	−0.81953400	1.86956400	0.18784900
	H7	2.02923800	−0.22498500	0.18640200
	H8	1.17252800	−1.67159400	−0.18697800
	H9	−1.20975100	−1.64457800	0.18733500
	H10	−2.03389600	−0.17943500	−0.18712000
Anion	N1	−0.65342100	−1.43332300	0.00000000

N2	0.65342800	−1.43330900	0.00000000
N3	1.11552800	−0.15197500	0.00000000
C4	0.00000000	0.57947300	0.00000000
N5	−1.11552100	−0.15199200	0.00000000
N6	−0.00001000	1.96951600	0.00000000
H7	0.87011600	2.46537900	0.00000000
H8	−0.87014500	2.46536400	0.00000000

SCF Done: E(B3LYP, cation) = −205.8356358 a.u.

SCF Done: E(B3LYP, anion) = −313.1609514 a.u.

Table S66: Salt 66

Cartesian coordinates

	Atom	X	Y	Z
Cation	N1	−0.71309600	1.63579100	0.00000000
	C2	0.00000000	0.49075400	0.00000000
	N3	1.34087600	0.55400700	0.00000000
	N4	−0.63397000	−0.66964200	0.00000000
	N5	−0.05044100	−1.92877000	0.00000000
	H6	−1.72036800	1.63935700	0.00000000
	H7	−0.25478500	2.53240900	0.00000000
	H8	1.92947100	−0.26070500	0.00000000
	H9	1.81369600	1.44397200	0.00000000
	H10	−1.64306300	−0.68139000	0.00000000
	H11	−0.67178300	−2.71571000	0.00000000
	H12	0.94325200	−2.04216900	0.00000000
Anion	N1	−0.65342100	−1.43332300	0.00000000
	N2	0.65342800	−1.43330900	0.00000000
	N3	1.11552800	−0.15197500	0.00000000
	C4	0.00000000	0.57947300	0.00000000
	N5	−1.11552100	−0.15199200	0.00000000
	N6	−0.00001000	1.96951600	0.00000000
	H7	0.87011600	2.46537900	0.00000000
	H8	−0.87014500	2.46536400	0.00000000

SCF Done: E(B3LYP, cation) = −261.143769 a.u.

SCF Done: E(B3LYP, anion) = −313.1609514 a.u.

Table S67: Salt 67

Cartesian coordinates

Atom	X	Y	Z

Cation	N1	−0.67062800	1.21160500	0.00003500
	C2	0.63691100	1.18917700	−0.00001800
	N3	1.09525400	−0.10727600	−0.00002800
	C4	0.00744600	−0.89435100	0.00000600
	N5	−1.04914100	−0.07890200	0.00001700
	N6	2.39810400	−0.50640700	−0.00006700
	C7	−2.47273800	−0.43828300	0.00006100
	H8	1.27858000	2.05575300	−0.00003600
	H9	−0.00304800	−1.97160800	0.00000600
	H10	3.10846400	0.20392000	−0.00009900
	H11	2.60559400	−1.48918100	−0.00007600
	H12	−2.55730100	−1.52323800	−0.00009900
	H13	−2.93850600	−0.02415500	0.89301100
	H14	−2.93861800	−0.02388600	−0.89270400
Anion	N1	−0.65342100	−1.43332300	0.00000000
	N2	0.65342800	−1.43330900	0.00000000
	N3	1.11552800	−0.15197500	0.00000000
	C4	0.00000000	0.57947300	0.00000000
	N5	−1.11552100	−0.15199200	0.00000000
	N6	−0.00001000	1.96951600	0.00000000
	H7	0.87011600	2.46537900	0.00000000
	H8	−0.87014500	2.46536400	0.00000000

SCF Done: E(B3LYP, cation) = −337.3261717 a.u.

SCF Done: E(B3LYP, anion) = −313.1609514 a.u.

Table S68: Salt68

Cartesian coordinates

	Atom	X	Y	Z
Cation	N1	−0.59982300	0.37475000	0.00003300
	C2	0.18818000	−0.70188400	−0.00004000
	N3	1.45301300	−0.24863300	−0.00002100
	C4	1.37531000	1.12268300	0.00005700
	N5	0.12574600	1.50822600	0.00006800
	C6	−2.08310200	0.45012700	0.00007500
	C7	−2.74410400	−0.91760400	−0.00006600
	N8	2.59284000	−0.99779000	−0.00007200
	H9	−0.11646900	−1.73397100	−0.00009600
	H10	2.23286600	1.77632500	0.00008500
	H11	−2.35238200	1.03043200	0.88364600
	H12	−2.35241300	1.03062900	−0.88335700
	H13	−3.82529800	−0.77126500	−0.00003900

	H14	−2.49786600	−1.49687700	−0.89341700
	H15	−2.49785300	−1.49706900	0.89315700
	H16	3.47355600	−0.51498900	−0.00005400
	H17	2.51572000	−1.99902500	−0.00013900
Anion	N1	−0.65342100	−1.43332300	0.00000000
	N2	0.65342800	−1.43330900	0.00000000
	N3	1.11552800	−0.15197500	0.00000000
	C4	0.00000000	0.57947300	0.00000000
	N5	−1.11552100	−0.15199200	0.00000000
	N6	−0.00001000	1.96951600	0.00000000
	H7	0.87011600	2.46537900	0.00000000
	H8	−0.87014500	2.46536400	0.00000000

SCF Done: E(B3LYP, cation) = −376.655599 a.u.

SCF Done: E(B3LYP, anion) = −313.1609514 a.u.

Table S69: Salt 69

Cartesian coordinates

	Atom	X	Y	Z
Cation	N1	−0.63269500	−1.51124800	−0.00011300
	N2	0.62505200	−1.53675300	−0.00001300
	N3	1.07010900	−0.23384900	0.00000600
	C4	0.01446400	0.60794500	−0.00009200
	N5	−1.05586200	−0.19902300	−0.00023900
	C6	−2.48172900	0.13532800	0.00023400
	N7	0.08185300	1.93048400	−0.00009700
	N8	2.38626800	0.17796600	0.00015600
	H9	−3.02338200	−0.80823600	−0.00003000
	H10	−2.73269300	0.69853500	0.90000200
	H11	−2.73312300	0.69925100	−0.89896200
	H12	0.99472500	2.36812900	0.00001100
	H13	−0.74240900	2.51138700	−0.00015600
	H14	2.85860500	−0.16092000	0.83499900
	H15	2.85878700	−0.16083300	−0.83461900
Anion	N1	−0.65342100	−1.43332300	0.00000000
	N2	0.65342800	−1.43330900	0.00000000
	N3	1.11552800	−0.15197500	0.00000000
	C4	0.00000000	0.57947300	0.00000000
	N5	−1.11552100	−0.15199200	0.00000000
	N6	−0.00001000	1.96951600	0.00000000
	H7	0.87011600	2.46537900	0.00000000
	H8	−0.87014500	2.46536400	0.00000000

SCF Done: E(B3LYP, cation) = −408.7379057 a.u.
SCF Done: E(B3LYP, anion) = −313.1609514 a.u.

Table S70: Salt 70
Cartesian coordinates

	Atom	X	Y	Z
Cation	N1	0.60584700	−1.52255200	0.00008900
	N2	−0.65427800	−1.50393700	−0.00028300
	N3	−1.07049400	−0.19824200	0.00003200
	C4	0.00675400	0.60547600	−0.00006000
	N5	1.06035500	−0.23187400	−0.00001500
	C6	2.49252300	0.07273700	0.00010900
	N7	0.04688700	1.93796900	−0.00011200
	C8	−2.50303000	0.11401600	0.00015500
	H9	3.01461000	−0.88165800	0.00024300
	H10	2.75697800	0.63012500	−0.89975200
	H11	2.75677200	0.63026100	0.89994600
	H12	−0.79576700	2.49123100	−0.00002800
	H13	0.92630800	2.43182100	−0.00019300
	H14	−2.63741500	1.19426500	0.00067300
	H15	−2.95858400	−0.30906800	−0.89398900
	H16	−2.95859700	−0.30990500	0.89389500
Anion	N1	−0.65342100	−1.43332300	0.00000000
	N2	0.65342800	−1.43330900	0.00000000
	N3	1.11552800	−0.15197500	0.00000000
	C4	0.00000000	0.57947300	0.00000000
	N5	−1.11552100	−0.15199200	0.00000000
	N6	−0.00001000	1.96951600	0.00000000
	H7	0.87011600	2.46537900	0.00000000
	H8	−0.87014500	2.46536400	0.00000000

SCF Done: E(B3LYP, cation) = −392.7171077 a.u.
SCF Done: E(B3LYP, anion) = −313.1609514 a.u.

Table S71: Salt 71
Cartesian coordinates

	Atom	X	Y	Z
Cation	N1	1.27417300	−0.01552500	0.00000500
	N2	0.55269800	−1.08300800	−0.00002100
	N3	−0.69851400	−0.61966400	−0.00000600

	C4	−0.66889800	0.74822700	0.00000100
	N5	0.60502600	1.13911600	−0.00009800
	C6	2.74552400	−0.06569500	0.00004900
	N7	−1.73412800	1.55395200	0.00002100
	C8	−1.84749600	−1.53064500	−0.00000200
	H9	3.09281800	0.96331900	−0.00039000
	H10	3.07745200	−0.58854200	0.89545800
	H11	3.07745600	−0.58931300	−0.89490600
	H12	−2.67778700	1.20353900	0.00023000
	H13	−1.59277400	2.55314800	0.00003200
	H14	−1.45920400	−2.54619500	−0.00015600
	H15	−2.44368800	−1.36579600	0.89843700
	H16	−2.44384400	−1.36558900	−0.89829900
Anion	N1	−0.65342100	−1.43332300	0.00000000
	N2	0.65342800	−1.43330900	0.00000000
	N3	1.11552800	−0.15197500	0.00000000
	C4	0.00000000	0.57947300	0.00000000
	N5	−1.11552100	−0.15199200	0.00000000
	N6	−0.00001000	1.96951600	0.00000000
	H7	0.87011600	2.46537900	0.00000000
	H8	−0.87014500	2.46536400	0.00000000

SCF Done: E(B3LYP, cation) = −392.7214667 a.u.

SCF Done: E(B3LYP, anion) = −313.1609514 a.u.

Table S72: Salt 72

Cartesian coordinates

	Atom	X	Y	Z
Cation	N1	−1.42331100	0.37619100	0.00000900
	N2	−1.53125400	−0.98612800	−0.00000800
	N3	−0.36799900	−1.46344200	0.00003100
	N4	0.53644700	−0.42712400	−0.00002100
	C5	−0.12717000	0.74116100	−0.00000700
	N6	0.36787200	1.97495300	0.00001600
	C7	1.97512400	−0.71509900	−0.00000300
	H8	−2.25955300	0.94762800	−0.00012500
	H9	1.36252400	2.14220400	−0.00004700
	H10	−0.23662100	2.78319300	0.00006300
	H11	2.53118400	0.22069200	−0.00040200
	H12	2.22129800	−1.28529400	0.89448400
	H13	2.22115600	−1.28594600	−0.89411100
Anion	C1	0.58282500	−1.22389200	−0.00000300

	C2	−0.79148400	−1.22195000	0.00001300
	C3	−1.49693900	−0.01630200	0.00001200
	C4	−0.81787400	1.20442100	0.00000600
	C5	0.55605200	1.23619400	−0.00000500
	C6	1.41054200	0.01531000	−0.00003500
	O7	2.63157300	0.02862800	−0.00015200
	N8	1.21373900	−2.54756700	0.00000400
	O9	2.43431100	−2.63285700	0.00013200
	O10	0.46971900	−3.54039400	−0.00014400
	N11	1.15804600	2.57326400	0.00000800
	O12	2.37646400	2.68515000	0.00031400
	O13	0.39260200	3.54965800	−0.00026500
	N14	−2.93812400	−0.03188200	0.00003700
	O15	−3.53876700	1.04886800	0.00003400
	O16	−3.51537200	−1.12530600	0.00004200
	H17	−1.32596500	−2.16021500	0.00002700
	H18	−1.37263400	2.13084200	0.00001500

SCF Done: E(B3LYP, cation) = −353.3859539 a.u.

SCF Done: E(B3LYP, anion) = −920.7359569 a.u.

Table S73: Salt 73

Cartesian coordinates

	Atom	X	Y	Z
Cation	C1	−1.08662300	−0.15534200	−0.00000800
	N2	−0.65252700	1.14258300	0.00002400
	N3	0.68150600	1.18024000	−0.00004600
	N4	1.00507800	−0.06381800	−0.00003300
	N5	−0.00612000	−0.93627200	−0.00010500
	C6	2.40732000	−0.50516200	0.00006900
	N7	−2.35101200	−0.57034900	0.00006500
	H8	−1.16666800	2.01634000	0.00002000
	H9	3.03008400	0.38546200	−0.00005900
	H10	2.57322900	−1.10420700	0.89413500
	H11	2.57324900	−1.10448700	−0.89380500
	H12	−3.13260900	0.06557500	0.00003500
	H13	−2.53993900	−1.56234600	−0.00002400
Anion	C1	0.58282500	−1.22389200	−0.00000300
	C2	−0.79148400	−1.22195000	0.00001300
	C3	−1.49693900	−0.01630200	0.00001200
	C4	−0.81787400	1.20442100	0.00000600
	C5	0.55605200	1.23619400	−0.00000500

	C6	1.41054200	0.01531000	−0.00003500
	O7	2.63157300	0.02862800	−0.00015200
	N8	1.21373900	−2.54756700	0.00000400
	O9	2.43431100	−2.63285700	0.00013200
	O10	0.46971900	−3.54039400	−0.00014400
	N11	1.15804600	2.57326400	0.00000800
	O12	2.37646400	2.68515000	0.00031400
	O13	0.39260200	3.54965800	−0.00026500
	N14	−2.93812400	−0.03188200	0.00003700
	O15	−3.53876700	1.04886800	0.00003400
	O16	−3.51537200	−1.12530600	0.00004200
	H17	−1.32596500	−2.16021500	0.00002700
	H18	−1.37263400	2.13084200	0.00001500

SCF Done: E(B3LYP, cation) = −353.391606 a.u.

SCF Done: E(B3LYP, anion) = −920.7359569 a.u.

Table S74: Salt 74

Cartesian coordinates

	Atom	X	Y	Z
Cation	N1	0.60584700	−1.52255200	0.00008900
	N2	−0.65427800	−1.50393700	−0.00028300
	N3	−1.07049400	−0.19824200	0.00003200
	C4	0.00675400	0.60547600	−0.00006000
	N5	1.06035500	−0.23187400	−0.00001500
	C6	2.49252300	0.07273700	0.00010900
	N7	0.04688700	1.93796900	−0.00011200
	C8	−2.50303000	0.11401600	0.00015500
	H9	3.01461000	−0.88165800	0.00024300
	H10	2.75697800	0.63012500	−0.89975200
	H11	2.75677200	0.63026100	0.89994600
	H12	−0.79576700	2.49123100	−0.00002800
	H13	0.92630800	2.43182100	−0.00019300
	H14	−2.63741500	1.19426500	0.00067300
	H15	−2.95858400	−0.30906800	−0.89398900
	H16	−2.95859700	−0.30990500	0.89389500
Anion	C1	0.58282500	−1.22389200	−0.00000300
	C2	−0.79148400	−1.22195000	0.00001300
	C3	−1.49693900	−0.01630200	0.00001200
	C4	−0.81787400	1.20442100	0.00000600
	C5	0.55605200	1.23619400	−0.00000500
	C6	1.41054200	0.01531000	−0.00003500

	O7	2.63157300	0.02862800	−0.00015200
	N8	1.21373900	−2.54756700	0.00000400
	O9	2.43431100	−2.63285700	0.00013200
	O10	0.46971900	−3.54039400	−0.00014400
	N11	1.15804600	2.57326400	0.00000800
	O12	2.37646400	2.68515000	0.00031400
	O13	0.39260200	3.54965800	−0.00026500
	N14	−2.93812400	−0.03188200	0.00003700
	O15	−3.53876700	1.04886800	0.00003400
	O16	−3.51537200	−1.12530600	0.00004200
	H17	−1.32596500	−2.16021500	0.00002700
	H18	−1.37263400	2.13084200	0.00001500

SCF Done: E(B3LYP, cation) = −392.7171077 a.u.

SCF Done: E(B3LYP, anion) = −920.7359569 a.u.

Table S75: Salt 75

Cartesian coordinates

	Atom	X	Y	Z
Cation	N1	0.60584700	−1.52255200	0.00008900
	N2	−0.65427800	−1.50393700	−0.00028300
	N3	−1.07049400	−0.19824200	0.00003200
	C4	0.00675400	0.60547600	−0.00006000
	N5	1.06035500	−0.23187400	−0.00001500
	C6	2.49252300	0.07273700	0.00010900
	N7	0.04688700	1.93796900	−0.00011200
	C8	−2.50303000	0.11401600	0.00015500
	H9	3.01461000	−0.88165800	0.00024300
	H10	2.75697800	0.63012500	−0.89975200
	H11	2.75677200	0.63026100	0.89994600
	H12	−0.79576700	2.49123100	−0.00002800
	H13	0.92630800	2.43182100	−0.00019300
	H14	−2.63741500	1.19426500	0.00067300
	H15	−2.95858400	−0.30906800	−0.89398900
	H16	−2.95859700	−0.30990500	0.89389500
Anion	N1	−0.65342100	−1.43332300	0.00000000
	N2	0.65342800	−1.43330900	0.00000000
	N3	1.11552800	−0.15197500	0.00000000
	C4	0.00000000	0.57947300	0.00000000
	N5	−1.11552100	−0.15199200	0.00000000
	N6	−0.00001000	1.96951600	0.00000000
	H7	0.87011600	2.46537900	0.00000000

	Atom	X	Y	Z
	H8	−0.87014500	2.46536400	0.00000000

SCF Done: E(B3LYP, cation) = −392.7171077 a.u.

SCF Done: E(B3LYP, anion) = −313.1609514 a.u.

Table S76: Salt 76

Cartesian coordinates

	Atom	X	Y	Z
Cation	N1	−1.42331100	0.37619100	0.00000900
	N2	−1.53125400	−0.98612800	−0.00000800
	N3	−0.36799900	−1.46344200	0.00003100
	N4	0.53644700	−0.42712400	−0.00002100
	C5	−0.12717000	0.74116100	−0.00000700
	N6	0.36787200	1.97495300	0.00001600
	C7	1.97512400	−0.71509900	−0.00000300
	H8	−2.25955300	0.94762800	−0.00012500
	H9	1.36252400	2.14220400	−0.00004700
	H10	−0.23662100	2.78319300	0.00006300
	H11	2.53118400	0.22069200	−0.00040200
	H12	2.22129800	−1.28529400	0.89448400
	H13	2.22115600	−1.28594600	−0.89411100
Anion	N1	−2.32247300	0.87855400	−0.00183500
	N2	−2.67204900	−0.36336800	0.00118900
	N3	−1.51240300	−1.05963800	0.00193100
	C4	−0.44794900	−0.20055100	−0.00041000
	N5	−0.97156800	1.03677900	−0.00288600
	N6	0.77606400	−0.78173600	−0.00100400
	N7	1.86650100	0.00782200	0.00028500
	O8	2.96076700	−0.59346500	−0.00249500
	O9	1.79276000	1.24847900	0.00420900
	H10	−1.48903200	−2.06571300	0.00499100

SCF Done: E(B3LYP, cation) = −353.3859539 a.u.

SCF Done: E(B3LYP, anion) = −517.7219762 a.u.

Table S77: Salt 77

Cartesian coordinates

	Atom	X	Y	Z
Cation	N1	0.72573800	−1.43573300	0.00000000
	C2	1.10022700	−0.18529000	0.00000000
	N3	0.00000000	0.65115400	0.00000000

	Atom	X	Y	Z
	C4	−1.08228900	−0.13710400	0.00000000
	N5	−0.61573600	−1.38850300	0.00000000
	N6	0.02662600	2.01061000	0.00000000
	H7	2.12050200	0.16391300	0.00000000
	H8	−2.11338600	0.17676100	0.00000000
	H9	−1.15210300	−2.24869500	0.00000000
	H10	0.92340300	2.46433500	0.00000000
	H11	−0.84244300	2.51536000	0.00000000
Anion	N1	−2.32247300	0.87855400	−0.00183500
	N2	−2.67204900	−0.36336800	0.00118900
	N3	−1.51240300	−1.05963800	0.00193100
	C4	−0.44794900	−0.20055100	−0.00041000
	N5	−0.97156800	1.03677900	−0.00288600
	N6	0.77606400	−0.78173600	−0.00100400
	N7	1.86650100	0.00782200	0.00028500
	O8	2.96076700	−0.59346500	−0.00249500
	O9	1.79276000	1.24847900	0.00420900
	H10	−1.48903200	−2.06571300	0.00499100

SCF Done: E(B3LYP, cation) = −297.9953294 a.u.

SCF Done: E(B3LYP, anion) = −517.7219762 a.u.

Table S78: Salt 78

Cartesian coordinates

	Atom	X	Y	Z
Cation	N1	−1.47534100	−0.62785300	0.00003000
	N2	−1.47587000	0.62752200	−0.00006500
	N3	−0.17047500	1.05257900	0.00004300
	C4	0.66687500	0.00044000	0.00000300
	N5	−0.17063300	−1.05243600	−0.00001100
	N6	1.99178000	−0.00011800	−0.00001900
	H7	0.03292400	2.04532000	0.00005500
	H8	0.03343500	−2.04505300	0.00011200
	H9	2.51851900	0.86180900	0.00006500
	H10	2.51764600	−0.86257100	−0.00009200
Anion	N1	−2.32247300	0.87855400	−0.00183500
	N2	−2.67204900	−0.36336800	0.00118900
	N3	−1.51240300	−1.05963800	0.00193100
	C4	−0.44794900	−0.20055100	−0.00041000
	N5	−0.97156800	1.03677900	−0.00288600
	N6	0.77606400	−0.78173600	−0.00100400
	N7	1.86650100	0.00782200	0.00028500

	Atom	X	Y	Z
	O8	2.96076700	−0.59346500	−0.00249500
	O9	1.79276000	1.24847900	0.00420900
	H10	−1.48903200	−2.06571300	0.00499100

SCF Done: E(B3LYP, cation) = −314.0552154 a.u.

SCF Done: E(B3LYP, anion) = −517.7219762 a.u.

Table S79: Salt 79

Cartesian coordinates

	Atom	X	Y	Z
Cation	C1	1.06640500	−0.41832800	−0.00003200
	N2	0.03611200	−1.21412900	−0.00000400
	N3	−1.03285300	−0.37700300	0.00003700
	C4	−0.67974900	0.89734900	−0.00005800
	N5	0.65865300	0.89769800	0.00002500
	H6	2.09788600	−0.73449200	−0.00004900
	H7	−1.97156900	−0.76356700	0.00008100
	H8	−1.33192900	1.75728600	−0.00010100
	H9	1.25228900	1.72069000	0.00019900
Anion	N1	−2.32247300	0.87855400	−0.00183500
	N2	−2.67204900	−0.36336800	0.00118900
	N3	−1.51240300	−1.05963800	0.00193100
	C4	−0.44794900	−0.20055100	−0.00041000
	N5	−0.97156800	1.03677900	−0.00288600
	N6	0.77606400	−0.78173600	−0.00100400
	N7	1.86650100	0.00782200	0.00028500
	O8	2.96076700	−0.59346500	−0.00249500
	O9	1.79276000	1.24847900	0.00420900
	H10	−1.48903200	−2.06571300	0.00499100

SCF Done: E(B3LYP, cation) = −242.6694066 a.u.

SCF Done: E(B3LYP, anion) = −517.7219762 a.u.

Table S80: Salt 80

Cartesian coordinates

	Atom	X	Y	Z
Cation	N1	1.40163300	−1.16761600	−0.00007100
	C2	2.51483200	−0.49231500	0.00013100
	N3	2.25644600	0.85524600	0.00007900
	C4	0.91889400	0.99427800	−0.00020700
	N5	0.41456800	−0.22667700	−0.00007100

	C6	−3.43539600	−0.01830700	0.00007700
	C7	−1.99159400	0.49998300	0.00007900
	C8	−1.00583700	−0.66235400	−0.00008000
	H9	3.50410500	−0.92217700	0.00027700
	H10	2.93320700	1.60949100	0.00033100
	H11	0.37374200	1.92323300	−0.00035800
	H12	−4.13480900	0.81855200	0.00017800
	H13	−3.64096400	−0.62465800	−0.88531000
	H14	−3.64090600	−0.62482500	0.88536300
	H5	−1.83161800	1.12538300	0.88534200
	H16	−1.83169100	1.12557000	−0.88506600
	H17	−1.12251300	−1.29236900	−0.88378500
	H18	−1.12247600	−1.29258600	0.88347700
Anion	N1	−2.32247300	0.87855400	−0.00183500
	N2	−2.67204900	−0.36336800	0.00118900
	N3	−1.51240300	−1.05963800	0.00193100
	C4	−0.44794900	−0.20055100	−0.00041000
	N5	−0.97156800	1.03677900	−0.00288600
	N6	0.77606400	−0.78173600	−0.00100400
	N7	1.86650100	0.00782200	0.00028500
	O8	2.96076700	−0.59346500	−0.00249500
	O9	1.79276000	1.24847900	0.00420900
	H10	−1.48903200	−2.06571300	0.00499100

SCF Done: E(B3LYP, cation) = −360.6577174 a.u.

SCF Done: E(B3LYP, anion) = −517.7219762 a.u.

Table S81: Salt 81

Cartesian coordinates

	Atom	X	Y	Z
Cation	N1	1.16549400	−1.15216600	−0.00009000
	C2	0.10928300	−0.37050500	−0.00001000
	N3	0.50786700	0.96531600	0.00002000
	C4	1.85243700	0.97271400	−0.00003500
	N5	2.21450300	−0.29340200	0.00006400
	N6	−1.15653700	−0.87371600	0.00003400
	N7	−2.11886300	−0.07468100	0.00000600
	N8	−3.09035500	0.48886000	0.00000100
	H9	−0.07760900	1.79315200	−0.00011500
	H10	2.49471700	1.83993100	−0.00001900
	H11	3.15781300	−0.66781200	0.00015800
Anion	N1	−2.32247300	0.87855400	−0.00183500

	N2	−2.67204900	−0.36336800	0.00118900
	N3	−1.51240300	−1.05963800	0.00193100
	C4	−0.44794900	−0.20055100	−0.00041000
	N5	−0.97156800	1.03677900	−0.00288600
	N6	0.77606400	−0.78173600	−0.00100400
	N7	1.86650100	0.00782200	0.00028500
	O8	2.96076700	−0.59346500	−0.00249500
	O9	1.79276000	1.24847900	0.00420900
	H10	−1.48903200	−2.06571300	0.00499100

SCF Done: E(B3LYP, cation) = −406.2900901 a.u.

SCF Done: E(B3LYP, anion) = −517.7219762 a.u.

Table S82: Salt 82

Cartesian coordinates

	Atom	X	Y	Z
Cation	N1	0.01505600	1.33482600	−0.00016400
	C2	0.00005600	0.00000600	0.00002800
	N3	1.14853800	−0.68050900	0.00012700
	N4	−1.16365100	−0.65433800	−0.00009900
	H5	0.86148500	1.85114100	−0.18670200
	H6	−0.81953400	1.86956400	0.18784900
	H7	2.02923800	−0.22498500	0.18640200
	H8	1.17252800	−1.67159400	−0.18697800
	H9	−1.20975100	−1.64457800	0.18733500
	H10	−2.03389600	−0.17943500	−0.18712000
Anion	N1	1.90351300	−1.54547200	−0.00199900
	N2	2.47467700	−0.38740100	0.00007700
	N3	1.47137600	0.52258100	0.00055500
	C4	0.26720600	−0.13807500	−0.00127600
	N5	0.55138700	−1.45154400	−0.00282800
	N6	−0.84142100	0.64269300	−0.00277100
	C7	1.72226000	1.94519700	0.00298900
	N8	−2.05277700	0.05897300	0.00028300
	O9	−3.02371300	0.84619000	−0.00418400
	O10	−2.20279300	−1.17448400	0.00740700
	H11	0.75268400	2.44088200	0.00194300
	H12	2.28606900	2.23075300	0.89503200
	H13	2.28921300	2.23318000	−0.88626600

SCF Done: E(B3LYP, cation) = −205.8356358 a.u.

SCF Done: E(B3LYP, anion) = −557.0437665 a.u.

Table S83: Salt 83

Cartesian coordinates

	Atom	X	Y	Z
Cation	N1	−0.71309600	1.63579100	0.00000000
	C2	0.00000000	0.49075400	0.00000000
	N3	1.34087600	0.55400700	0.00000000
	N4	−0.63397000	−0.66964200	0.00000000
	N5	−0.05044100	−1.92877000	0.00000000
	H6	−1.72036800	1.63935700	0.00000000
	H7	−0.25478500	2.53240900	0.00000000
	H8	1.92947100	−0.26070500	0.00000000
	H9	1.81369600	1.44397200	0.00000000
	H10	−1.64306300	−0.68139000	0.00000000
	H11	−0.67178300	−2.71571000	0.00000000
	H12	0.94325200	−2.04216900	0.00000000
Anion	N1	1.90351300	−1.54547200	−0.00199900
	N2	2.47467700	−0.38740100	0.00007700
	N3	1.47137600	0.52258100	0.00055500
	C4	0.26720600	−0.13807500	−0.00127600
	N5	0.55138700	−1.45154400	−0.00282800
	N6	−0.84142100	0.64269300	−0.00277100
	C7	1.72226000	1.94519700	0.00298900
	N8	−2.05277700	0.05897300	0.00028300
	O9	−3.02371300	0.84619000	−0.00418400
	O10	−2.20279300	−1.17448400	0.00740700
	H11	0.75268400	2.44088200	0.00194300
	H12	2.28606900	2.23075300	0.89503200
	H13	2.28921300	2.23318000	−0.88626600

SCF Done: E(B3LYP, cation) = −261.143769 a.u.

SCF Done: E(B3LYP, anion) = −557.0437665 a.u.

Table S84: Salt 84

Cartesian coordinates

	Atom	X	Y	Z
Cation	N1	0.00000000	2.42944600	−0.09893800
	N2	0.00000000	1.14998700	−0.65450900
	C3	0.00000000	0.00000000	0.02528500
	N4	0.00000000	−1.14998700	−0.65450900
	N5	0.00000000	−2.42944600	−0.09893800

	N6	0.00000000	0.00000000	1.37233500
	H7	0.00000000	3.19851000	−0.74127800
	H8	0.00000000	2.56996300	0.89042200
	H9	0.00000000	1.13876300	−1.66204000
	H10	0.00000000	−1.13876300	−1.66204000
	H11	0.00000000	−3.19851000	−0.74127800
	H12	0.00000000	−2.56996300	0.89042200
	H13	0.00000000	−0.84963300	1.90800300
	H14	0.00000000	0.84963300	1.90800300
Anion	N1	1.90351300	−1.54547200	−0.00199900
	N2	2.47467700	−0.38740100	0.00007700
	N3	1.47137600	0.52258100	0.00055500
	C4	0.26720600	−0.13807500	−0.00127600
	N5	0.55138700	−1.45154400	−0.00282800
	N6	−0.84142100	0.64269300	−0.00277100
	C7	1.72226000	1.94519700	0.00298900
	N8	−2.05277700	0.05897300	0.00028300
	O9	−3.02371300	0.84619000	−0.00418400
	O10	−2.20279300	−1.17448400	0.00740700
	H11	0.75268400	2.44088200	0.00194300
	H12	2.28606900	2.23075300	0.89503200
	H13	2.28921300	2.23318000	−0.88626600

SCF Done: E(B3LYP, cation) = −316.4385003 a.u.

SCF Done: E(B3LYP, anion) = −557.0437665 a.u.

Table S85: Salt 85

Cartesian coordinates

	Atom	X	Y	Z
Cation	N1	−0.73090600	1.42465800	0.00000000
	C2	0.00000000	0.28737400	0.00000000
	N3	1.33364100	0.45849100	0.00000000
	N4	−0.65902100	−0.87579600	0.00000000
	N5	2.38734200	−0.46354400	0.00000000
	N6	−0.19531500	−2.19352200	0.00000000
	N7	−2.12655000	1.51794100	0.00000000
	H8	−0.26473700	2.31587700	0.00000000
	H9	1.66735400	1.40846300	0.00000000
	H10	−1.66209300	−0.83875500	0.00000000
	H11	3.30922600	−0.07173500	0.00000000
	H12	2.24095500	−1.44742300	0.00000000
	H13	−0.89921200	−2.90635300	0.00000000

	H14	0.77363900	−2.41899000	0.00000000
	H15	−2.51076700	2.44330700	0.00000000
	H16	−2.71870400	0.71376600	0.00000000
Anion	N1	1.90351300	−1.54547200	−0.00199900
	N2	2.47467700	−0.38740100	0.00007700
	N3	1.47137600	0.52258100	0.00055500
	C4	0.26720600	−0.13807500	−0.00127600
	N5	0.55138700	−1.45154400	−0.00282800
	N6	−0.84142100	0.64269300	−0.00277100
	C7	1.72226000	1.94519700	0.00298900
	N8	−2.05277700	0.05897300	0.00028300
	O9	−3.02371300	0.84619000	−0.00418400
	O10	−2.20279300	−1.17448400	0.00740700
	H11	0.75268400	2.44088200	0.00194300
	H12	2.28606900	2.23075300	0.89503200
	H13	2.28921300	2.23318000	−0.88626600

SCF Done: E(B3LYP, cation) = −371.7391764 a.u.

SCF Done: E(B3LYP, anion) = −557.0437665 a.u.

Table S86: Salt 86

Cartesian coordinates

	Atom	X	Y	Z
Cation	N1	−0.68202200	1.93302800	0.00000000
	C2	0.00000000	0.79762100	0.00000000
	N3	1.33107100	0.77740900	0.00000000
	N4	−0.78337800	−0.32289900	0.00000000
	N5	−0.22337400	−1.44878300	0.00000000
	N6	0.10039400	−2.52020000	0.00000000
	H7	−1.69293200	1.89950700	0.00000000
	H8	−0.23394500	2.83769500	0.00000000
	H9	1.85682500	−0.08467500	0.00000000
	H10	1.87121500	1.63185600	0.00000000
Anion	N1	1.90351300	−1.54547200	−0.00199900
	N2	2.47467700	−0.38740100	0.00007700
	N3	1.47137600	0.52258100	0.00055500
	C4	0.26720600	−0.13807500	−0.00127600
	N5	0.55138700	−1.45154400	−0.00282800
	N6	−0.84142100	0.64269300	−0.00277100
	C7	1.72226000	1.94519700	0.00298900
	N8	−2.05277700	0.05897300	0.00028300
	O9	−3.02371300	0.84619000	−0.00418400

	O10	−2.20279300	−1.17448400	0.00740700
	H11	0.75268400	2.44088200	0.00194300
	H12	2.28606900	2.23075300	0.89503200
	H13	2.28921300	2.23318000	−0.88626600

SCF Done: E(B3LYP, cation) = −314.0656767 a.u.

SCF Done: E(B3LYP, anion) = −557.0437665 a.u.

Table S87: Salt 87

Cartesian coordinates

	Atom	X	Y	Z
Cation	C1	−0.67468300	0.00935200	−0.00001100
	N2	0.12778500	−1.06305900	0.00037200
	N3	1.46009600	−0.72163800	−0.00059300
	C4	1.46120000	0.56763800	0.00028800
	N5	0.16620400	1.07016600	−0.00002300
	N6	−2.00636100	0.03613400	−0.00011800
	H7	−0.13122800	−2.04067600	0.00063500
	H8	2.34086100	1.19145000	0.00034700
	H9	−0.09999700	2.04618100	0.00021400
	H10	−2.54623400	−0.81626400	0.00015500
	H11	−2.51656700	0.90615400	−0.00048200
Anion	N1	0.67195600	2.05851800	0.00000000
	N2	−0.67221000	2.05845700	0.00000000
	N3	−1.11928500	0.81026500	0.00000000
	C4	0.00000000	0.07854900	0.00000000
	N5	1.11920100	0.81038500	0.00000000
	N6	0.00008100	−1.36159900	0.00000000
	O7	1.08603400	−1.94390800	0.00000000
	O8	−1.08580900	−1.94402600	0.00000000

SCF Done: E(B3LYP, cation) = −298.0636709 a.u.

SCF Done: E(B3LYP, anion) = −462.3675711 a.u.

Table S88: Salt 88

Cartesian coordinates

	Atom	X	Y	Z
Cation	N1	1.13908000	1.02887800	0.00012700
	N2	1.84297800	−0.02428100	−0.00001800
	N3	0.98945400	−1.07733700	−0.00006300
	C4	−0.28251500	−0.66614700	0.00001400
	N5	−0.17767500	0.66418700	−0.00002000

	C6	−1.21991400	1.70107100	−0.00005700
	C7	−1.50611900	−1.50347000	0.00004900
	H8	1.35542800	−2.02391100	−0.00020800
	H9	−2.19481100	1.21927500	−0.00039900
	H10	−1.10178000	2.31242300	0.89333900
	H11	−1.10134100	2.31279800	−0.89313600
	H12	−1.24471600	−2.56122200	−0.00017600
	H13	−2.10903400	−1.29425900	0.88730100
	H14	−2.10930800	−1.29395100	−0.88694100
Anion	N1	0.67195600	2.05851800	0.00000000
	N2	−0.67221000	2.05845700	0.00000000
	N3	−1.11928500	0.81026500	0.00000000
	C4	0.00000000	0.07854900	0.00000000
	N5	1.11920100	0.81038500	0.00000000
	N6	0.00008100	−1.36159900	0.00000000
	O7	1.08603400	−1.94390800	0.00000000
	O8	−1.08580900	−1.94402600	0.00000000

SCF Done: E(B3LYP, cation) = −337.3360546 a.u.

SCF Done: E(B3LYP, anion) = −462.3675711 a.u.

Table S89: Salt 89

Cartesian coordinates

	Atom	X	Y	Z
Cation	N1	−1.47534100	−0.62785300	0.00003000
	N2	−1.47587000	0.62752200	−0.00006500
	N3	−0.17047500	1.05257900	0.00004300
	C4	0.66687500	0.00044000	0.00000300
	N5	−0.17063300	−1.05243600	−0.00001100
	N6	1.99178000	−0.00011800	−0.00001900
	H7	0.03292400	2.04532000	0.00005500
	H8	0.03343500	−2.04505300	0.00011200
	H9	2.51851900	0.86180900	0.00006500
	H10	2.51764600	−0.86257100	−0.00009200
Anion	N1	0.67195600	2.05851800	0.00000000
	N2	−0.67221000	2.05845700	0.00000000
	N3	−1.11928500	0.81026500	0.00000000
	C4	0.00000000	0.07854900	0.00000000
	N5	1.11920100	0.81038500	0.00000000
	N6	0.00008100	−1.36159900	0.00000000
	O7	1.08603400	−1.94390800	0.00000000
	O8	−1.08580900	−1.94402600	0.00000000

SCF Done: E(B3LYP, cation) = −314.0552154 a.u.

SCF Done: E(B3LYP, anion) = −462.3675711 a.u.

Table S90: Salt 90

Cartesian coordinates

	Atom	X	Y	Z
Cation	N1	0.95084300	−1.17263000	−0.00095200
	N2	1.79153100	−0.22542300	0.00058800
	N3	1.10330500	0.94038700	0.00025100
	C4	−0.22970700	0.71021600	−0.00009100
	N5	−0.30366700	−0.62651500	−0.00007800
	N6	−1.38823800	−1.44672200	0.00003000
	N7	−1.22440700	1.60185200	−0.00028100
	H8	1.60344900	1.82132800	0.00070400
	H9	−1.18849000	−2.43403400	0.00065800
	H10	−2.31486600	−1.06327600	0.00306000
	H11	−2.19333300	1.32523000	−0.00046100
	H12	−1.03408900	2.59281600	−0.00032600
Anion	N1	0.67195600	2.05851800	0.00000000
	N2	−0.67221000	2.05845700	0.00000000
	N3	−1.11928500	0.81026500	0.00000000
	C4	0.00000000	0.07854900	0.00000000
	N5	1.11920100	0.81038500	0.00000000
	N6	0.00008100	−1.36159900	0.00000000
	O7	1.08603400	−1.94390800	0.00000000
	O8	−1.08580900	−1.94402600	0.00000000

SCF Done: E(B3LYP, cation) = −369.3807307 a.u.

SCF Done: E(B3LYP, anion) = −462.3675711 a.u.

Table S91: Salt 91

Cartesian coordinates

	Atom	X	Y	Z
Cation	N1	0.01505600	1.33482600	−0.00016400
	C2	0.00005600	0.00000600	0.00002800
	N3	1.14853800	−0.68050900	0.00012700
	N4	−1.16365100	−0.65433800	−0.00009900
	H5	0.86148500	1.85114100	−0.18670200
	H6	−0.81953400	1.86956400	0.18784900
	H7	2.02923800	−0.22498500	0.18640200

	H8	1.17252800	−1.67159400	−0.18697800
	H9	−1.20975100	−1.64457800	0.18733500
	H10	−2.03389600	−0.17943500	−0.18712000
Anion	N1	−2.61644400	−2.70900200	0.00000000
	N2	−1.36097900	−3.10512500	0.00000000
	N3	−0.53208100	−2.03354200	0.00000000
	C4	−1.37410600	−1.01060100	0.00000000
	N5	−2.66455800	−1.36581900	0.00000000
	N6	−1.11669900	0.37771600	0.00000000
	C7	0.00000000	1.13043900	0.00000000
	N8	−0.20677100	2.47584100	0.00000000
	N9	1.18230100	0.50638500	0.00000000
	N10	2.32550500	1.23860400	0.00000000
	O11	3.36905500	0.59625800	0.00000000
	O12	2.34069800	2.50334500	0.00000000
	H13	−1.99470100	0.87833500	0.00000000
	H14	0.62593000	3.05339800	0.00000000
	H15	−1.13653800	2.85700400	0.00000000

SCF Done: E(B3LYP, cation) = −205.8356358 a.u.

SCF Done: E(B3LYP, anion) = −666.5842593 a.u.

Table S92: Salt 92

Cartesian coordinates

	Atom	X	Y	Z
Cation	N1	−0.71309600	1.63579100	0.00000000
	C2	0.00000000	0.49075400	0.00000000
	N3	1.34087600	0.55400700	0.00000000
	N4	−0.63397000	−0.66964200	0.00000000
	N5	−0.05044100	−1.92877000	0.00000000
	H6	−1.72036800	1.63935700	0.00000000
	H7	−0.25478500	2.53240900	0.00000000
	H8	1.92947100	−0.26070500	0.00000000
	H9	1.81369600	1.44397200	0.00000000
	H10	−1.64306300	−0.68139000	0.00000000
	H11	−0.67178300	−2.71571000	0.00000000
	H12	0.94325200	−2.04216900	0.00000000
Anion	N1	−2.61644400	−2.70900200	0.00000000
	N2	−1.36097900	−3.10512500	0.00000000
	N3	−0.53208100	−2.03354200	0.00000000
	C4	−1.37410600	−1.01060100	0.00000000
	N5	−2.66455800	−1.36581900	0.00000000

	N6	−1.11669900	0.37771600	0.00000000
	C7	0.00000000	1.13043900	0.00000000
	N8	−0.20677100	2.47584100	0.00000000
	N9	1.18230100	0.50638500	0.00000000
	N10	2.32550500	1.23860400	0.00000000
	O11	3.36905500	0.59625800	0.00000000
	O12	2.34069800	2.50334500	0.00000000
	H13	−1.99470100	0.87833500	0.00000000
	H14	0.62593000	3.05339800	0.00000000
	H15	−1.13653800	2.85700400	0.00000000

SCF Done: E(B3LYP, cation) = −261.143769 a.u.

SCF Done: E(B3LYP, anion) = −666.5842593 a.u.

Table S93: Salt 93

Cartesian coordinates

	Atom	X	Y	Z
Cation	N1	0.00000000	2.42944600	−0.09893800
	N2	0.00000000	1.14998700	−0.65450900
	C3	0.00000000	0.00000000	0.02528500
	N4	0.00000000	−1.14998700	−0.65450900
	N5	0.00000000	−2.42944600	−0.09893800
	N6	0.00000000	0.00000000	1.37233500
	H7	0.00000000	3.19851000	−0.74127800
	H8	0.00000000	2.56996300	0.89042200
	H9	0.00000000	1.13876300	−1.66204000
	H10	0.00000000	−1.13876300	−1.66204000
	H11	0.00000000	−3.19851000	−0.74127800
	H12	0.00000000	−2.56996300	0.89042200
	H13	0.00000000	−0.84963300	1.90800300
	H14	0.00000000	0.84963300	1.90800300
Anion	N1	−2.61644400	−2.70900200	0.00000000
	N2	−1.36097900	−3.10512500	0.00000000
	N3	−0.53208100	−2.03354200	0.00000000
	C4	−1.37410600	−1.01060100	0.00000000
	N5	−2.66455800	−1.36581900	0.00000000
	N6	−1.11669900	0.37771600	0.00000000
	C7	0.00000000	1.13043900	0.00000000
	N8	−0.20677100	2.47584100	0.00000000
	N9	1.18230100	0.50638500	0.00000000
	N10	2.32550500	1.23860400	0.00000000
	O11	3.36905500	0.59625800	0.00000000

	O12	2.34069800	2.50334500	0.00000000
	H13	−1.99470100	0.87833500	0.00000000
	H14	0.62593000	3.05339800	0.00000000
	H15	−1.13653800	2.85700400	0.00000000

SCF Done: E(B3LYP, cation) = −316.4385003 a.u.

SCF Done: E(B3LYP, anion) = −666.5842593 a.u.

Table S94: Salt 94

Cartesian coordinates

	Atom	X	Y	Z
Cation	N1	−1.26743958	−0.76210486	−0.11251109
	C2	−0.27680858	0.11490214	0.09680091
	N3	−2.62460158	−0.63154486	0.22361791
	N4	0.99154442	−0.35337886	0.08387891
	N5	2.06350642	0.54806814	0.11087491
	N6	−0.45997458	1.42000114	0.32222491
	N7	−1.63937558	2.07287214	−0.08446609
	H8	−1.02863758	−1.65502386	−0.52334309
	H9	−2.73284058	−0.61934086	1.23390591
	H10	−2.99096558	0.22618414	−0.17552609
	H11	1.15006942	−1.34982986	0.17710691
	H12	1.29594258	1.19219586	0.27820109
	H13	2.62334342	0.40968814	0.94722791
	H14	2.64196342	0.43870114	−0.71719109
	H15	−1.96709158	2.66545914	0.67256191
	H16	−1.44770758	2.64882414	−0.90086409
Anion	N1	−2.61644400	−2.70900200	0.00000000
	N2	−1.36097900	−3.10512500	0.00000000
	N3	−0.53208100	−2.03354200	0.00000000
	C4	−1.37410600	−1.01060100	0.00000000
	N5	−2.66455800	−1.36581900	0.00000000
	N6	−1.11669900	0.37771600	0.00000000
	C7	0.00000000	1.13043900	0.00000000
	N8	−0.20677100	2.47584100	0.00000000
	N9	1.18230100	0.50638500	0.00000000
	N10	2.32550500	1.23860400	0.00000000
	O11	3.36905500	0.59625800	0.00000000
	O12	2.34069800	2.50334500	0.00000000
	H13	−1.99470100	0.87833500	0.00000000
	H14	0.62593000	3.05339800	0.00000000
	H15	−1.13653800	2.85700400	0.00000000

SCF Done: E(B3LYP, cation) = −371.8407102 a.u.

SCF Done: E(B3LYP, anion) = −666.5842593 a.u.

Table S95: Salt 95

Cartesian coordinates

	Atom	X	Y	Z
Cation	N1	2.31218400	0.76862300	0.00000000
	C2	1.20229600	0.02255600	0.00000000
	N3	0.00000000	0.66359500	0.00000000
	N4	1.26145600	−1.29536300	0.00000000
	C5	−1.27820400	0.00713000	0.00000000
	N6	−2.32216300	0.85491600	0.00000000
	O7	−1.35801900	−1.20338400	0.00000000
	H8	3.22630700	0.34135400	0.00000000
	H9	2.28234200	1.77628500	0.00000000
	H10	0.02372300	1.67372500	0.00000000
	H11	0.37690600	−1.80805400	0.00000000
	H12	2.14512200	−1.78250700	0.00000000
	H13	−3.24569300	0.44611200	0.00000000
	H14	−2.24945800	1.85963800	0.00000000
Anion	N1	−2.61644400	−2.70900200	0.00000000
	N2	−1.36097900	−3.10512500	0.00000000
	N3	−0.53208100	−2.03354200	0.00000000
	C4	−1.37410600	−1.01060100	0.00000000
	N5	−2.66455800	−1.36581900	0.00000000
	N6	−1.11669900	0.37771600	0.00000000
	C7	0.00000000	1.13043900	0.00000000
	N8	−0.20677100	2.47584100	0.00000000
	N9	1.18230100	0.50638500	0.00000000
	N10	2.32550500	1.23860400	0.00000000
	O11	3.36905500	0.59625800	0.00000000
	O12	2.34069800	2.50334500	0.00000000
	H13	−1.99470100	0.87833500	0.00000000
	H14	0.62593000	3.05339800	0.00000000
	H15	−1.13653800	2.85700400	0.00000000

SCF Done: E(B3LYP, cation) = −374.5987024 a.u.

SCF Done: E(B3LYP, anion) = −666.5842593 a.u.

Table S96: Salt 96

Cartesian coordinates

	Atom	X	Y	Z
Cation	N1	−1.23126100	−0.61392200	−0.00005700
	C2	−0.06944600	0.01029100	−0.00002200
	N3	−2.41564800	0.10939600	−0.00009600
	N4	1.12200500	−0.76734500	0.00008400
	N5	2.24162600	0.08278500	0.00006100
	O6	0.09192400	1.23885400	0.00003400
	H7	−1.31636200	−1.61920200	−0.00004200
	H8	−3.27674900	−0.40124900	−0.00008900
	H9	−2.33833700	1.11046000	−0.00012000
	H10	1.25457300	−1.76698500	0.00002500
	H11	1.70267300	1.03124200	0.00000300
	H12	2.81920300	−0.00159200	0.84339400
	H13	2.81922300	−0.00165200	−0.84325300
Anion	N1	−2.61644400	−2.70900200	0.00000000
	N2	−1.36097900	−3.10512500	0.00000000
	N3	−0.53208100	−2.03354200	0.00000000
	C4	−1.37410600	−1.01060100	0.00000000
	N5	−2.66455800	−1.36581900	0.00000000
	N6	−1.11669900	0.37771600	0.00000000
	C7	0.00000000	1.13043900	0.00000000
	N8	−0.20677100	2.47584100	0.00000000
	N9	1.18230100	0.50638500	0.00000000
	N10	2.32550500	1.23860400	0.00000000
	O11	3.36905500	0.59625800	0.00000000
	O12	2.34069800	2.50334500	0.00000000
	H13	−1.99470100	0.87833500	0.00000000
	H14	0.62593000	3.05339800	0.00000000
	H15	−1.13653800	2.85700400	0.00000000

SCF Done: E(B3LYP, cation) = −336.3373235 a.u.

SCF Done: E(B3LYP, anion) = −666.5842593 a.u.

Table S97: Salt 97

Cartesian coordinates

	Atom	X	Y	Z
Cation	N1	−0.39033800	1.87880800	−0.00009500
	C2	−0.65775000	0.58470300	−0.00009100
	N3	−1.99058900	0.17360200	0.00015500
	N4	−2.14178400	−1.22860500	0.00000100
	N5	0.13883600	−0.46530000	−0.00009200

	N6	1.54801100	−0.32932400	0.00002000
	O7	2.14711400	−1.37170000	−0.00003100
	O8	2.02664500	0.80094200	0.00014300
	H9	0.58973600	2.14741300	−0.00025000
	H10	−1.11132200	2.58517400	0.00002500
	H11	−2.80652400	0.76708000	0.00022100
	H12	−1.05236600	−1.48783800	−0.00041100
	H13	−2.60580500	−1.57923800	0.84408800
	H14	−2.60623400	−1.57900700	−0.84394600
Anion	N1	−2.61644400	−2.70900200	0.00000000
	N2	−1.36097900	−3.10512500	0.00000000
	N3	−0.53208100	−2.03354200	0.00000000
	C4	−1.37410600	−1.01060100	0.00000000
	N5	−2.66455800	−1.36581900	0.00000000
	N6	−1.11669900	0.37771600	0.00000000
	C7	0.00000000	1.13043900	0.00000000
	N8	−0.20677100	2.47584100	0.00000000
	N9	1.18230100	0.50638500	0.00000000
	N10	2.32550500	1.23860400	0.00000000
	O11	3.36905500	0.59625800	0.00000000
	O12	2.34069800	2.50334500	0.00000000
	H13	−1.99470100	0.87833500	0.00000000
	H14	0.62593000	3.05339800	0.00000000
	H15	−1.13653800	2.85700400	0.00000000

SCF Done: E(B3LYP, cation) = −465.6555662 a.u.

SCF Done: E(B3LYP, anion) = −666.5842593 a.u.

Table S98: Salt 98

Cartesian coordinates

	Atom	X	Y	Z
Cation	N1	0.63151800	−1.53175700	0.00017900
	N2	−0.63750600	−1.53002600	−0.00069800
	N3	−1.05571500	−0.23777700	0.00005900
	C4	0.00062000	0.58293300	−0.00017600
	N5	1.05612100	−0.24001700	0.00002300
	C6	2.49454500	0.06414800	0.00026300
	C7	0.00297500	2.06623300	−0.00026200
	C8	−2.49346800	0.06453600	0.00041600
	H9	3.01255800	−0.89198200	0.00052200
	H10	2.75264700	0.62484100	−0.89737600
	H11	2.75226100	0.62510600	0.89784600

	Atom	X	Y	Z
	H12	1.02206200	2.44947700	−0.00006000
	H13	−0.50977200	2.44820700	−0.88677800
	H14	−0.51016600	2.44831600	0.88597600
	H15	−2.62545000	1.14379800	0.00106400
	H16	−2.94165000	−0.36839200	−0.89268300
	H17	−2.94145100	−0.36943600	0.89310700
Anion	N1	−2.61644400	−2.70900200	0.00000000
	N2	−1.36097900	−3.10512500	0.00000000
	N3	−0.53208100	−2.03354200	0.00000000
	C4	−1.37410600	−1.01060100	0.00000000
	N5	−2.66455800	−1.36581900	0.00000000
	N6	−1.11669900	0.37771600	0.00000000
	C7	0.00000000	1.13043900	0.00000000
	N8	−0.20677100	2.47584100	0.00000000
	N9	1.18230100	0.50638500	0.00000000
	N10	2.32550500	1.23860400	0.00000000
	O11	3.36905500	0.59625800	0.00000000
	O12	2.34069800	2.50334500	0.00000000
	H13	−1.99470100	0.87833500	0.00000000
	H14	0.62593000	3.05339800	0.00000000
	H15	−1.13653800	2.85700400	0.00000000

SCF Done: E(B3LYP, cation) = −376.6673328 a.u.

SCF Done: E(B3LYP, anion) = −666.5842593 a.u.

Table S99: Salt 99

Cartesian coordinates

	Atom	X	Y	Z
Cation	N1	0.60584700	−1.52255200	0.00008900
	N2	−0.65427800	−1.50393700	−0.00028300
	N3	−1.07049400	−0.19824200	0.00003200
	C4	0.00675400	0.60547600	−0.00006000
	N5	1.06035500	−0.23187400	−0.00001500
	C6	2.49252300	0.07273700	0.00010900
	N7	0.04688700	1.93796900	−0.00011200
	C8	−2.50303000	0.11401600	0.00015500
	H9	3.01461000	−0.88165800	0.00024300
	H10	2.75697800	0.63012500	−0.89975200
	H11	2.75677200	0.63026100	0.89994600
	H12	−0.79576700	2.49123100	−0.00002800
	H13	0.92630800	2.43182100	−0.00019300
	H14	−2.63741500	1.19426500	0.00067300

	H15	−2.95858400	−0.30906800	−0.89398900
	H16	−2.95859700	−0.30990500	0.89389500
Anion	N1	−2.61644400	−2.70900200	0.00000000
	N2	−1.36097900	−3.10512500	0.00000000
	N3	−0.53208100	−2.03354200	0.00000000
	C4	−1.37410600	−1.01060100	0.00000000
	N5	−2.66455800	−1.36581900	0.00000000
	N6	−1.11669900	0.37771600	0.00000000
	C7	0.00000000	1.13043900	0.00000000
	N8	−0.20677100	2.47584100	0.00000000
	N9	1.18230100	0.50638500	0.00000000
	N10	2.32550500	1.23860400	0.00000000
	O11	3.36905500	0.59625800	0.00000000
	O12	2.34069800	2.50334500	0.00000000
	H13	−1.99470100	0.87833500	0.00000000
	H14	0.62593000	3.05339800	0.00000000
	H15	−1.13653800	2.85700400	0.00000000

SCF Done: E(B3LYP, cation) = −392.7171077 a.u.

SCF Done: E(B3LYP, anion) = −666.5842593 a.u.

Table S100: Salt 100

Cartesian coordinates

	Atom	X	Y	Z
Cation	C1	−0.93563500	−0.44076800	1.10484500
	C2	−1.23017700	1.06824900	0.78225000
	N3	−0.03668700	1.49735700	0.11426700
	C4	0.93779000	0.51701700	0.10567000
	N5	0.39582300	−0.63018600	0.61295000
	N6	−1.98026300	−1.13838600	0.32629600
	C7	−2.74428200	−0.29187600	−0.36956600
	N8	−2.41956500	0.97595600	−0.09283900
	N9	−3.71245800	−0.65796400	−1.20204100
	N10	2.11750100	0.81141500	−0.34160500
	N11	3.09401000	−0.20931600	−0.31432000
	O12	4.12378300	0.06300600	−0.87523900
	O13	2.86243900	−1.27923500	0.27060200
	H14	−1.03465800	−0.70595400	2.15770800
	H15	−1.47438000	1.67616500	1.65352200
	H16	0.25814600	2.46303400	0.08001700
	H17	1.03230300	−1.38354100	0.85526100
	H18	−2.05280800	−2.14382800	0.27536900

	H19	−2.79488200	1.77163400	−0.58804200
	H20	−4.27963800	0.02517500	−1.68129500
	H21	−3.91855700	−1.63071700	−1.37358600
Anion	N1	−2.61644400	−2.70900200	0.00000000
	N2	−1.36097900	−3.10512500	0.00000000
	N3	−0.53208100	−2.03354200	0.00000000
	C4	−1.37410600	−1.01060100	0.00000000
	N5	−2.66455800	−1.36581900	0.00000000
	N6	−1.11669900	0.37771600	0.00000000
	C7	0.00000000	1.13043900	0.00000000
	N8	−0.20677100	2.47584100	0.00000000
	N9	1.18230100	0.50638500	0.00000000
	N10	2.32550500	1.23860400	0.00000000
	O11	3.36905500	0.59625800	0.00000000
	O12	2.34069800	2.50334500	0.00000000
	H13	−1.99470100	0.87833500	0.00000000
	H14	0.62593000	3.05339800	0.00000000
	H15	−1.13653800	2.85700400	0.00000000

SCF Done: E(B3LYP, cation) = −690.819319 a.u.

SCF Done: E(B3LYP, anion) = −666.5842593 a.u.

Table S101: Salt 101

Cartesian coordinates

	Atom	X	Y	Z
Cation	N1	−0.00001200	0.00000000	0.00000000
	H2	0.09263200	0.83857300	−0.58434300
	H3	0.10779100	−0.83730900	−0.58355400
	H4	0.72571300	0.00690600	0.72562600
	H5	−0.92605200	−0.00816900	0.44227100
Anion	N1	2.80157900	0.71401300	−0.00000900
	N2	3.00370900	−0.59546200	0.00005600
	N3	1.83247700	−1.24297800	−0.00000500
	C4	0.91587000	−0.26585200	0.00000000
	N5	1.48783600	0.95606100	0.00002600
	C6	−0.52956300	−0.40499200	−0.00001700
	N7	−1.21858900	−1.51344400	−0.00004500
	O8	−2.54597900	−1.14967400	−0.00008600
	N9	−2.68894200	0.24496000	−0.00001000
	C10	−1.46557700	0.70561100	0.00001400
	N11	−1.10914400	2.01489800	0.00007000
	H12	−0.10936200	2.20889500	0.00007000

	H13	−1.80967200	2.73356000	0.00007100

SCF Done: E(B3LYP, cation) = −56.9203571 a.u.

SCF Done: E(B3LYP, anion) = −574.1182786 a.u.

Table S102: Salt 102

Cartesian coordinates

	Atom	X	Y	Z
Cation	C1	−0.16030900	1.13421300	0.00001100
	N2	0.64022800	0.00541000	−0.00001400
	C3	−0.14235700	−1.07565000	0.00000300
	N4	−1.41450500	−0.66298500	0.00000500
	C5	−1.45516100	0.71501800	−0.00000800
	C6	2.11612700	−0.00840000	0.00000300
	H7	0.25356300	2.12801300	0.00001300
	H8	0.19084100	−2.10049900	−0.00000100
	H9	−2.21768000	−1.27914800	0.00000700
	H10	−2.37804200	1.26900300	−0.00001200
	H11	2.46634300	1.02069300	−0.00026000
	H12	2.47754500	−0.51284000	0.89485800
	H13	2.47756200	−0.51328600	−0.89459400
Anion	N1	2.80157900	0.71401300	−0.00000900
	N2	3.00370900	−0.59546200	0.00005600
	N3	1.83247700	−1.24297800	−0.00000500
	C4	0.91587000	−0.26585200	0.00000000
	N5	1.48783600	0.95606100	0.00002600
	C6	−0.52956300	−0.40499200	−0.00001700
	N7	−1.21858900	−1.51344400	−0.00004500
	O8	−2.54597900	−1.14967400	−0.00008600
	N9	−2.68894200	0.24496000	−0.00001000
	C10	−1.46557700	0.70561100	0.00001400
	N11	−1.10914400	2.01489800	0.00007000
	H12	−0.10936200	2.20889500	0.00007000
	H13	−1.80967200	2.73356000	0.00007100

SCF Done: E(B3LYP, cation) = −265.9810056 a.u.

SCF Done: E(B3LYP, anion) = −574.1182786 a.u.

Table S103: Salt 103

Cartesian coordinates

	Atom	X	Y	Z
Cation	N1	0.65541700	−0.00411800	0.00005100

	N2	−0.75045600	−0.00232700	−0.00003900
	H3	0.99718400	0.96411800	−0.00012100
	H4	1.05415400	−0.45557600	0.83700000
	H5	1.05425800	−0.45589200	−0.83667700
	H6	−1.21844400	−0.89213100	−0.00011000
	H7	−1.22187800	0.88459500	−0.00017400
Anion	N1	2.80157900	0.71401300	−0.00000900
	N2	3.00370900	−0.59546200	0.00005600
	N3	1.83247700	−1.24297800	−0.00000500
	C4	0.91587000	−0.26585200	0.00000000
	N5	1.48783600	0.95606100	0.00002600
	C6	−0.52956300	−0.40499200	−0.00001700
	N7	−1.21858900	−1.51344400	−0.00004500
	O8	−2.54597900	−1.14967400	−0.00008600
	N9	−2.68894200	0.24496000	−0.00001000
	C10	−1.46557700	0.70561100	0.00001400
	N11	−1.10914400	2.01489800	0.00007000
	H12	−0.10936200	2.20889500	0.00007000
	H13	−1.80967200	2.73356000	0.00007100

SCF Done: E(B3LYP, cation) = −112.2418137 a.u.

SCF Done: E(B3LYP, anion) = −574.1182786 a.u.

Table S104: Salt 104

Cartesian coordinates

	Atom	X	Y	Z
Cation	N1	0.01505600	1.33482600	−0.00016400
	C2	0.00005600	0.00000600	0.00002800
	N3	1.14853800	−0.68050900	0.00012700
	N4	−1.16365100	−0.65433800	−0.00009900
	H5	0.86148500	1.85114100	−0.18670200
	H6	−0.81953400	1.86956400	0.18784900
	H7	2.02923800	−0.22498500	0.18640200
	H8	1.17252800	−1.67159400	−0.18697800
	H9	−1.20975100	−1.64457800	0.18733500
	H10	−2.03389600	−0.17943500	−0.18712000
Anion	N1	2.80157900	0.71401300	−0.00000900
	N2	3.00370900	−0.59546200	0.00005600
	N3	1.83247700	−1.24297800	−0.00000500
	C4	0.91587000	−0.26585200	0.00000000
	N5	1.48783600	0.95606100	0.00002600
	C6	−0.52956300	−0.40499200	−0.00001700

	N7	−1.21858900	−1.51344400	−0.00004500
	O8	−2.54597900	−1.14967400	−0.00008600
	N9	−2.68894200	0.24496000	−0.00001000
	C10	−1.46557700	0.70561100	0.00001400
	N11	−1.10914400	2.01489800	0.00007000
	H12	−0.10936200	2.20889500	0.00007000
	H13	−1.80967200	2.73356000	0.00007100

SCF Done: E(B3LYP, cation) = −205.8356358 a.u.

SCF Done: E(B3LYP, anion) = −574.1182786 a.u.

Table S105: Salt 105

Cartesian coordinates

	Atom	X	Y	Z
Cation	N1	−0.71309600	1.63579100	0.00000000
	C2	0.00000000	0.49075400	0.00000000
	N3	1.34087600	0.55400700	0.00000000
	N4	−0.63397000	−0.66964200	0.00000000
	N5	−0.05044100	−1.92877000	0.00000000
	H6	−1.72036800	1.63935700	0.00000000
	H7	−0.25478500	2.53240900	0.00000000
	H8	1.92947100	−0.26070500	0.00000000
	H9	1.81369600	1.44397200	0.00000000
	H10	−1.64306300	−0.68139000	0.00000000
	H11	−0.67178300	−2.71571000	0.00000000
	H12	0.94325200	−2.04216900	0.00000000
Anion	N1	2.80157900	0.71401300	−0.00000900
	N2	3.00370900	−0.59546200	0.00005600
	N3	1.83247700	−1.24297800	−0.00000500
	C4	0.91587000	−0.26585200	0.00000000
	N5	1.48783600	0.95606100	0.00002600
	C6	−0.52956300	−0.40499200	−0.00001700
	N7	−1.21858900	−1.51344400	−0.00004500
	O8	−2.54597900	−1.14967400	−0.00008600
	N9	−2.68894200	0.24496000	−0.00001000
	C10	−1.46557700	0.70561100	0.00001400
	N11	−1.10914400	2.01489800	0.00007000
	H12	−0.10936200	2.20889500	0.00007000
	H13	−1.80967200	2.73356000	0.00007100

SCF Done: E(B3LYP, cation) = −261.143769 a.u.

SCF Done: E(B3LYP, anion) = −574.1182786 a.u.

Table S106: Salt 106

Cartesian coordinates

	Atom	X	Y	Z
Cation	N1	0.00000000	2.42944600	−0.09893800
	N2	0.00000000	1.14998700	−0.65450900
	C3	0.00000000	0.00000000	0.02528500
	N4	0.00000000	−1.14998700	−0.65450900
	N5	0.00000000	−2.42944600	−0.09893800
	N6	0.00000000	0.00000000	1.37233500
	H7	0.00000000	3.19851000	−0.74127800
	H8	0.00000000	2.56996300	0.89042200
	H9	0.00000000	1.13876300	−1.66204000
	H10	0.00000000	−1.13876300	−1.66204000
	H11	0.00000000	−3.19851000	−0.74127800
	H12	0.00000000	−2.56996300	0.89042200
	H13	0.00000000	−0.84963300	1.90800300
	H14	0.00000000	0.84963300	1.90800300
Anion	N1	2.80157900	0.71401300	−0.00000900
	N2	3.00370900	−0.59546200	0.00005600
	N3	1.83247700	−1.24297800	−0.00000500
	C4	0.91587000	−0.26585200	0.00000000
	N5	1.48783600	0.95606100	0.00002600
	C6	−0.52956300	−0.40499200	−0.00001700
	N7	−1.21858900	−1.51344400	−0.00004500
	O8	−2.54597900	−1.14967400	−0.00008600
	N9	−2.68894200	0.24496000	−0.00001000
	C10	−1.46557700	0.70561100	0.00001400
	N11	−1.10914400	2.01489800	0.00007000
	H12	−0.10936200	2.20889500	0.00007000
	H13	−1.80967200	2.73356000	0.00007100

SCF Done: E(B3LYP, cation) = −316.4385003 a.u.

SCF Done: E(B3LYP, anion) = −574.1182786 a.u.

Table S107: Salt 107

Cartesian coordinates

	Atom	X	Y	Z
Cation	N1	−0.73090600	1.42465800	0.00000000
	C2	0.00000000	0.28737400	0.00000000
	N3	1.33364100	0.45849100	0.00000000

	N4	−0.65902100	−0.87579600	0.00000000
	N5	2.38734200	−0.46354400	0.00000000
	N6	−0.19531500	−2.19352200	0.00000000
	N7	−2.12655000	1.51794100	0.00000000
	H8	−0.26473700	2.31587700	0.00000000
	H9	1.66735400	1.40846300	0.00000000
	H10	−1.66209300	−0.83875500	0.00000000
	H11	3.30922600	−0.07173500	0.00000000
	H12	2.24095500	−1.44742300	0.00000000
	H13	−0.89921200	−2.90635300	0.00000000
	H14	0.77363900	−2.41899000	0.00000000
	H15	−2.51076700	2.44330700	0.00000000
	H16	−2.71870400	0.71376600	0.00000000
Anion	N1	2.80157900	0.71401300	−0.00000900
	N2	3.00370900	−0.59546200	0.00005600
	N3	1.83247700	−1.24297800	−0.00000500
	C4	0.91587000	−0.26585200	0.00000000
	N5	1.48783600	0.95606100	0.00002600
	C6	−0.52956300	−0.40499200	−0.00001700
	N7	−1.21858900	−1.51344400	−0.00004500
	O8	−2.54597900	−1.14967400	−0.00008600
	N9	−2.68894200	0.24496000	−0.00001000
	C10	−1.46557700	0.70561100	0.00001400
	N11	−1.10914400	2.01489800	0.00007000
	H12	−0.10936200	2.20889500	0.00007000
	H13	−1.80967200	2.73356000	0.00007100

SCF Done: E(B3LYP, cation) = −371.7391764 a.u.

SCF Done: E(B3LYP, anion) = −574.1182786 a.u.

Table S108: Salt 108

Cartesian coordinates

	Atom	X	Y	Z
Cation	N1	2.31218400	0.76862300	0.00000000
	C2	1.20229600	0.02255600	0.00000000
	N3	0.00000000	0.66359500	0.00000000
	N4	1.26145600	−1.29536300	0.00000000
	C5	−1.27820400	0.00713000	0.00000000
	N6	−2.32216300	0.85491600	0.00000000
	O7	−1.35801900	−1.20338400	0.00000000
	H8	3.22630700	0.34135400	0.00000000
	H9	2.28234200	1.77628500	0.00000000

	H10	0.02372300	1.67372500	0.00000000
	H11	0.37690600	−1.80805400	0.00000000
	H12	2.14512200	−1.78250700	0.00000000
	H13	−3.24569300	0.44611200	0.00000000
	H14	−2.24945800	1.85963800	0.00000000
Anion	N1	2.80157900	0.71401300	−0.00000900
	N2	3.00370900	−0.59546200	0.00005600
	N3	1.83247700	−1.24297800	−0.00000500
	C4	0.91587000	−0.26585200	0.00000000
	N5	1.48783600	0.95606100	0.00002600
	C6	−0.52956300	−0.40499200	−0.00001700
	N7	−1.21858900	−1.51344400	−0.00004500
	O8	−2.54597900	−1.14967400	−0.00008600
	N9	−2.68894200	0.24496000	−0.00001000
	C10	−1.46557700	0.70561100	0.00001400
	N11	−1.10914400	2.01489800	0.00007000
	H12	−0.10936200	2.20889500	0.00007000
	H13	−1.80967200	2.73356000	0.00007100

SCF Done: E(B3LYP, cation) = −374.5987024 a.u.

SCF Done: E(B3LYP, anion) = −574.1182786 a.u.

Table S109: Salt 109

Cartesian coordinates

	Atom	X	Y	Z
Cation	N1	−2.30865200	0.81588100	0.00000000
	C2	−1.21694900	0.03718400	0.00000000
	N3	0.00000000	0.64962600	0.00000000
	N4	−1.29726400	−1.27578000	0.00000000
	C5	1.27171000	0.00451000	0.00000000
	N6	2.30605800	0.88525700	0.00000000
	N7	1.30701300	−1.27093700	0.00000000
	H8	−3.23217400	0.41098700	0.00000000
	H9	−2.25308100	1.82198600	0.00000000
	H10	−0.00498400	1.65842700	0.00000000
	H11	−0.38525300	−1.77463900	0.00000000
	H12	−2.18726700	−1.75083000	0.00000000
	H13	3.25154100	0.53751900	0.00000000
	H14	2.19157500	1.88552200	0.00000000
	H15	2.24099900	−1.66747000	0.00000000
Anion	N1	2.80157900	0.71401300	−0.00000900
	N2	3.00370900	−0.59546200	0.00005600

	N3	1.83247700	−1.24297800	−0.00000500
	C4	0.91587000	−0.26585200	0.00000000
	N5	1.48783600	0.95606100	0.00002600
	C6	−0.52956300	−0.40499200	−0.00001700
	N7	−1.21858900	−1.51344400	−0.00004500
	O8	−2.54597900	−1.14967400	−0.00008600
	N9	−2.68894200	0.24496000	−0.00001000
	C10	−1.46557700	0.70561100	0.00001400
	N11	−1.10914400	2.01489800	0.00007000
	H12	−0.10936200	2.20889500	0.00007000
	H13	−1.80967200	2.73356000	0.00007100

SCF Done: E(B3LYP, cation) = −354.7051979 a.u.

SCF Done: E(B3LYP, anion) = −574.1182786 a.u.

Table S110: Salt 110

Cartesian coordinates

	Atom	X	Y	Z
Cation	N1	−1.23126100	−0.61392200	−0.00005700
	C2	−0.06944600	0.01029100	−0.00002200
	N3	−2.41564800	0.10939600	−0.00009600
	N4	1.12200500	−0.76734500	0.00008400
	N5	2.24162600	0.08278500	0.00006100
	O6	0.09192400	1.23885400	0.00003400
	H7	−1.31636200	−1.61920200	−0.00004200
	H8	−3.27674900	−0.40124900	−0.00008900
	H9	−2.33833700	1.11046000	−0.00012000
	H10	1.25457300	−1.76698500	0.00002500
	H11	1.70267300	1.03124200	0.00000300
	H12	2.81920300	−0.00159200	0.84339400
	H13	2.81922300	−0.00165200	−0.84325300
Anion	N1	2.80157900	0.71401300	−0.00000900
	N2	3.00370900	−0.59546200	0.00005600
	N3	1.83247700	−1.24297800	−0.00000500
	C4	0.91587000	−0.26585200	0.00000000
	N5	1.48783600	0.95606100	0.00002600
	C6	−0.52956300	−0.40499200	−0.00001700
	N7	−1.21858900	−1.51344400	−0.00004500
	O8	−2.54597900	−1.14967400	−0.00008600
	N9	−2.68894200	0.24496000	−0.00001000
	C10	−1.46557700	0.70561100	0.00001400
	N11	−1.10914400	2.01489800	0.00007000

H12	−0.10936200	2.20889500	0.00007000
H13	−1.80967200	2.73356000	0.00007100

SCF Done: E(B3LYP, cation) = −336.3373235 a.u.

SCF Done: E(B3LYP, anion) = −574.1182786 a.u.

Table S111: Salt 111

Cartesian coordinates

	Atom	X	Y	Z
Cation	C1	−0.59451600	1.20187700	−0.00003200
	N2	0.70987100	1.21521700	−0.00001400
	N3	1.06725400	−0.09451300	0.00000000
	C4	0.00077400	−0.88021700	−0.00013000
	N5	−1.08025000	−0.08242400	−0.00002500
	C6	−2.49413600	−0.50489600	0.00008000
	C7	2.48650600	−0.47089700	0.00007100
	H8	−1.22032700	2.08055400	−0.00001100
	H9	−0.00234600	−1.95863900	−0.00020400
	H10	−3.11391800	0.38900000	0.00000800
	H11	−2.70184400	−1.08933900	−0.89524100
	H12	−2.70178700	−1.08916200	0.89553200
	H13	2.55902600	−1.55669300	0.00020400
	H14	2.95566400	−0.05954800	−0.89231300
	H15	2.95564100	−0.05933200	0.89236600
Anion	N1	2.80157900	0.71401300	−0.00000900
	N2	3.00370900	−0.59546200	0.00005600
	N3	1.83247700	−1.24297800	−0.00000500
	C4	0.91587000	−0.26585200	0.00000000
	N5	1.48783600	0.95606100	0.00002600
	C6	−0.52956300	−0.40499200	−0.00001700
	N7	−1.21858900	−1.51344400	−0.00004500
	O8	−2.54597900	−1.14967400	−0.00008600
	N9	−2.68894200	0.24496000	−0.00001000
	C10	−1.46557700	0.70561100	0.00001400
	N11	−1.10914400	2.01489800	0.00007000
	H12	−0.10936200	2.20889500	0.00007000
	H13	−1.80967200	2.73356000	0.00007100

SCF Done: E(B3LYP, cation) = −321.329351 a.u.

SCF Done: E(B3LYP, anion) = −574.1182786 a.u.

Table S112: Salt 112

Cartesian coordinates

	Atom	X	Y	Z
Cation	N1	0.63151800	−1.53175700	0.00017900
	N2	−0.63750600	−1.53002600	−0.00069800
	N3	−1.05571500	−0.23777700	0.00005900
	C4	0.00062000	0.58293300	−0.00017600
	N5	1.05612100	−0.24001700	0.00002300
	C6	2.49454500	0.06414800	0.00026300
	C7	0.00297500	2.06623300	−0.00026200
	C8	−2.49346800	0.06453600	0.00041600
	H9	3.01255800	−0.89198200	0.00052200
	H10	2.75264700	0.62484100	−0.89737600
	H11	2.75226100	0.62510600	0.89784600
	H12	1.02206200	2.44947700	−0.00006000
	H13	−0.50977200	2.44820700	−0.88677800
	H14	−0.51016600	2.44831600	0.88597600
	H15	−2.62545000	1.14379800	0.00106400
	H16	−2.94165000	−0.36839200	−0.89268300
	H17	−2.94145100	−0.36943600	0.89310700
Anion	N1	2.80157900	0.71401300	−0.00000900
	N2	3.00370900	−0.59546200	0.00005600
	N3	1.83247700	−1.24297800	−0.00000500
	C4	0.91587000	−0.26585200	0.00000000
	N5	1.48783600	0.95606100	0.00002600
	C6	−0.52956300	−0.40499200	−0.00001700
	N7	−1.21858900	−1.51344400	−0.00004500
	O8	−2.54597900	−1.14967400	−0.00008600
	N9	−2.68894200	0.24496000	−0.00001000
	C10	−1.46557700	0.70561100	0.00001400
	N11	−1.10914400	2.01489800	0.00007000
	H12	−0.10936200	2.20889500	0.00007000
	H13	−1.80967200	2.73356000	0.00007100

SCF Done: E(B3LYP, cation) = −376.6673328 a.u.

SCF Done: E(B3LYP, anion) = −574.1182786 A.U

Table S113: Salt 113

Cartesian coordinates

	Atom	X	Y	Z
Cation	N1	0.60584700	−1.52255200	0.00008900
	N2	−0.65427800	−1.50393700	−0.00028300

	N3	−1.07049400	−0.19824200	0.00003200
	C4	0.00675400	0.60547600	−0.00006000
	N5	1.06035500	−0.23187400	−0.00001500
	C6	2.49252300	0.07273700	0.00010900
	N7	0.04688700	1.93796900	−0.00011200
	C8	−2.50303000	0.11401600	0.00015500
	H9	3.01461000	−0.88165800	0.00024300
	H10	2.75697800	0.63012500	−0.89975200
	H11	2.75677200	0.63026100	0.89994600
	H12	−0.79576700	2.49123100	−0.00002800
	H13	0.92630800	2.43182100	−0.00019300
	H14	−2.63741500	1.19426500	0.00067300
	H15	−2.95858400	−0.30906800	−0.89398900
	H16	−2.95859700	−0.30990500	0.89389500
Anion	N1	2.80157900	0.71401300	−0.00000900
	N2	3.00370900	−0.59546200	0.00005600
	N3	1.83247700	−1.24297800	−0.00000500
	C4	0.91587000	−0.26585200	0.00000000
	N5	1.48783600	0.95606100	0.00002600
	C6	−0.52956300	−0.40499200	−0.00001700
	N7	−1.21858900	−1.51344400	−0.00004500
	O8	−2.54597900	−1.14967400	−0.00008600
	N9	−2.68894200	0.24496000	−0.00001000
	C10	−1.46557700	0.70561100	0.00001400
	N11	−1.10914400	2.01489800	0.00007000
	H12	−0.10936200	2.20889500	0.00007000
	H13	−1.80967200	2.73356000	0.00007100

SCF Done: E(B3LYP, cation) = −392.7171077 a.u.

SCF Done: E(B3LYP, anion) = −574.1182786 a.u.

Table S114: Salt 114

Cartesian coordinates

	Atom	X	Y	Z
Cation	C1	−0.67569800	0.42810400	−1.21284500
	C2	0.69084800	0.98423600	−0.83358600
	N3	1.50822200	0.00317200	0.00559000
	C4	0.67576900	−0.42786800	1.21290300
	C5	−0.69082100	−0.98410200	0.83384700
	N6	−1.50835400	−0.00320300	−0.00556300
	N7	−2.73768500	−0.67633900	−0.35499600
	N8	−1.97373700	1.18092400	0.68413700

	N9	2.73807600	0.67601800	0.35473700
	N10	1.97338600	−1.18101900	−0.68427700
	H11	−0.60085700	−0.44934200	−1.85746400
	H12	−1.24740700	1.19668900	−1.73392700
	H13	1.27182300	1.19844600	−1.73153400
	H14	0.61444600	1.90982700	−0.25969200
	H15	0.60090000	0.44967900	1.85739000
	H16	1.24743700	−1.19638200	1.73413900
	H17	−1.27179300	−1.19813300	1.73183000
	H18	−0.61444900	−1.90981200	0.26015200
	H19	−2.53680000	−1.49154200	−0.93190700
	H20	−3.31871800	−0.01503500	−0.87267700
	H21	−1.20653000	1.65625400	1.15106500
	H22	−2.66217800	0.87606400	1.37422200
	H23	2.53725300	1.49157500	0.93123700
	H24	3.31831300	0.01469300	0.87334700
	H25	1.20624700	−1.65593100	−1.15168700
	H26	2.66237800	−0.87613200	−1.37379700
Anion	N1	−0.36965900	2.81889800	0.00000000
	N2	0.94888300	2.68000500	0.00000000
	N3	1.26302000	1.37463600	0.00000000
	C4	0.07711200	0.73207500	0.00000000
	N5	−0.94888300	1.60762500	0.00000000
	C6	−0.07711200	−0.73207500	0.00000000
	N7	−1.26302000	−1.37463600	0.00000000
	N8	−0.94888300	−2.68000500	0.00000000
	N9	0.36965900	−2.81889800	0.00000000
	N10	0.94888300	−1.60762500	0.00000000

SCF Done: E(B3LYP, cation) = −489.9479396 a.u.

SCF Done: E(B3LYP, anion) = −514.2825578 a.u.

Table S115: Salt 115

Cartesian coordinates

	Atom	X	Y	Z
Cation	N1	0.65541700	−0.00411800	0.00005100
	N2	−0.75045600	−0.00232700	−0.00003900
	H3	0.99718400	0.96411800	−0.00012100
	H4	1.05415400	−0.45557600	0.83700000
	H5	1.05425800	−0.45589200	−0.83667700
	H6	−1.21844400	−0.89213100	−0.00011000
	H7	−1.22187800	0.88459500	−0.00017400

Anion	C1	0.00000000	0.37938600	0.00000000
	N2	−0.42855600	1.63963700	0.00000000
	N3	−1.77005300	1.50771800	0.00000000
	N4	−2.09637900	0.22834300	0.00000000
	N5	−0.97985900	−0.52614600	0.00000000
	N6	1.38152500	0.07435500	0.00000000
	N7	1.71473300	−1.10429000	0.00000000
	N8	2.17859000	−2.14480400	0.00000000

SCF Done: E(B3LYP, cation) = −112.2418137 a.u.

SCF Done: E(B3LYP, anion) = −421.4323883 a.u.

Table S116: Salt 116

Cartesian coordinates

	Atom	X	Y	Z
Cation	N1	−0.00001200	0.00000000	0.00000000
	H2	0.09263200	0.83857300	−0.58434300
	H3	0.10779100	−0.83730900	−0.58355400
	H4	0.72571300	0.00690600	0.72562600
	H5	−0.92605200	−0.00816900	0.44227100
Anion	C1	0.00000000	0.37938600	0.00000000
	N2	−0.42855600	1.63963700	0.00000000
	N3	−1.77005300	1.50771800	0.00000000
	N4	−2.09637900	0.22834300	0.00000000
	N5	−0.97985900	−0.52614600	0.00000000
	N6	1.38152500	0.07435500	0.00000000
	N7	1.71473300	−1.10429000	0.00000000
	N8	2.17859000	−2.14480400	0.00000000

SCF Done: E(B3LYP, cation) = −56.9203571 a.u.

SCF Done: E(B3LYP, anion) = −421.4323883 a.u.

Table S117: Salt 117

Cartesian coordinates

	Atom	X	Y	Z
Cation	N1	0.01505600	1.33482600	−0.00016400
	C2	0.00005600	0.00000600	0.00002800

	N3	1.14853800	−0.68050900	0.00012700
	N4	−1.16365100	−0.65433800	−0.00009900
	H5	0.86148500	1.85114100	−0.18670200
	H6	−0.81953400	1.86956400	0.18784900
	H7	2.02923800	−0.22498500	0.18640200
	H8	1.17252800	−1.67159400	−0.18697800
	H9	−1.20975100	−1.64457800	0.18733500
	H10	−2.03389600	−0.17943500	−0.18712000
Anion	C1	0.00000000	0.37938600	0.00000000
	N2	−0.42855600	1.63963700	0.00000000
	N3	−1.77005300	1.50771800	0.00000000
	N4	−2.09637900	0.22834300	0.00000000
	N5	−0.97985900	−0.52614600	0.00000000
	N6	1.38152500	0.07435500	0.00000000
	N7	1.71473300	−1.10429000	0.00000000
	N8	2.17859000	−2.14480400	0.00000000

SCF Done: E(B3LYP, cation) = −205.8356358 a.u.

SCF Done: E(B3LYP, anion) = −421.4323883 a.u.

Table S118: Salt 118

Cartesian coordinates

	Atom	X	Y	Z
Cation	N1	−0.71309600	1.63579100	0.00000000
	C2	0.00000000	0.49075400	0.00000000
	N3	1.34087600	0.55400700	0.00000000
	N4	−0.63397000	−0.66964200	0.00000000
	N5	−0.05044100	−1.92877000	0.00000000
	H6	−1.72036800	1.63935700	0.00000000
	H7	−0.25478500	2.53240900	0.00000000
	H8	1.92947100	−0.26070500	0.00000000
	H9	1.81369600	1.44397200	0.00000000
	H10	−1.64306300	−0.68139000	0.00000000
	H11	−0.67178300	−2.71571000	0.00000000
	H12	0.94325200	−2.04216900	0.00000000
Anion	C1	0.00000000	0.37938600	0.00000000
	N2	−0.42855600	1.63963700	0.00000000
	N3	−1.77005300	1.50771800	0.00000000
	N4	−2.09637900	0.22834300	0.00000000
	N5	−0.97985900	−0.52614600	0.00000000
	N6	1.38152500	0.07435500	0.00000000
	N7	1.71473300	−1.10429000	0.00000000

	N8	2.17859000	−2.14480400	0.00000000

SCF Done: E(B3LYP, cation) = −261.143769 a.u.

SCF Done: E(B3LYP, anion) = −421.4323883 a.u.

Table S119: Salt 119

Cartesian coordinates

	Atom	X	Y	Z
Cation	C1	1.73873200	−1.43272900	0.06523700
	C2	2.78115000	−0.53199600	−0.09618600
	C3	2.58272300	0.83836400	−0.08296900
	C4	1.28876100	1.30925800	0.08646600
	C5	0.18564400	0.45946600	0.27650200
	C6	0.47216300	−0.91411900	0.27061800
	C7	−1.19631900	0.94965900	0.46912400
	N8	−2.13865200	0.28782100	−0.07950600
	N9	−3.42660700	0.69305100	0.12274000
	C10	−4.41899100	−0.09219600	−0.35406800
	N11	−5.68230800	0.33106500	−0.24703800
	N12	−4.11616000	−1.25828800	−0.90322900
	N13	−0.62189000	−1.88684500	0.53065600
	O14	−1.07337100	−1.92087600	1.65905600
	O15	−0.98524100	−2.57172500	−0.41467900
	N16	1.09775600	2.78355000	0.00382200
	O17	−0.03649000	3.18356500	−0.24463100
	O18	2.07313700	3.48293000	0.17674500
	N19	4.16165300	−1.06148900	−0.30003200
	O20	5.04731300	−0.23940300	−0.44505100
	O21	4.28523500	−2.27384100	−0.30307900
	H22	1.92627500	−2.49880100	0.04810600
	H23	3.40941000	1.52275400	−0.21875300
	H24	−1.36434000	1.83442100	1.07924800
	H25	−3.64335000	1.52660700	0.66309300
	H26	−6.45564100	−0.26488200	−0.50002900
	H27	−5.90473400	1.26755400	0.05394700
	H28	−4.81945600	−1.83307300	−1.34151400
	H29	−3.15254800	−1.57608700	−0.91123000
Anion	C1	1.18847300	1.30965400	0.00000000
	N2	2.44302700	0.81478800	0.00000000
	N3	3.22740900	1.89149100	0.00000000
	N4	2.47494200	2.99969600	0.00000000
	N5	1.18847300	2.65915100	0.00000000

	N6	−0.03688000	0.62878200	0.00000000
	N7	0.03688000	−0.62878200	0.00000000
	C8	−1.18847300	−1.30965400	0.00000000
	N9	−2.44302700	−0.81478800	0.00000000
	N10	−3.22740900	−1.89149100	0.00000000
	N11	−2.47494200	−2.99969600	0.00000000
	N12	−1.18847300	−2.65915100	0.00000000

SCF Done: E(B3LYP, cation) = −1144.0380666 a.u.

SCF Done: E(B3LYP, anion) = −623.7876564 a.u.

Table S120: Salt 120

Cartesian coordinates

	Atom	X	Y	Z
Cation	C1	1.73873200	−1.43272900	0.06523700
	C2	2.78115000	−0.53199600	−0.09618600
	C3	2.58272300	0.83836400	−0.08296900
	C4	1.28876100	1.30925800	0.08646600
	C5	0.18564400	0.45946600	0.27650200
	C6	0.47216300	−0.91411900	0.27061800
	C7	−1.19631900	0.94965900	0.46912400
	N8	−2.13865200	0.28782100	−0.07950600
	N9	−3.42660700	0.69305100	0.12274000
	C10	−4.41899100	−0.09219600	−0.35406800
	N11	−5.68230800	0.33106500	−0.24703800
	N12	−4.11616000	−1.25828800	−0.90322900
	N13	−0.62189000	−1.88684500	0.53065600
	O14	−1.07337100	−1.92087600	1.65905600
	O15	−0.98524100	−2.57172500	−0.41467900
	N16	1.09775600	2.78355000	0.00382200
	O17	−0.03649000	3.18356500	−0.24463100
	O18	2.07313700	3.48293000	0.17674500
	N19	4.16165300	−1.06148900	−0.30003200
	O20	5.04731300	−0.23940300	−0.44505100
	O21	4.28523500	−2.27384100	−0.30307900
	H22	1.92627500	−2.49880100	0.04810600
	H23	3.40941000	1.52275400	−0.21875300
	H24	−1.36434000	1.83442100	1.07924800
	H25	−3.64335000	1.52660700	0.66309300
	H26	−6.45564100	−0.26488200	−0.50002900
	H27	−5.90473400	1.26755400	0.05394700
	H28	−4.81945600	−1.83307300	−1.34151400

	H29	−3.15254800	−1.57608700	−0.91123000
Anion	N1	−3.97109800	−1.75691000	1.87261300
	N2	−2.98430100	−1.03901700	1.33777000
	C3	−3.60589500	−0.19915300	0.49279600
	N4	−4.94010300	−0.37919200	0.48746600
	N5	−5.13769100	−1.36719600	1.35956200
	C6	−2.91663900	0.79966300	−0.30367000
	N7	−3.49857500	1.89095200	−0.74118100
	O8	−2.53466200	2.56555700	−1.48307000
	N9	−1.34649500	1.90459000	−1.48073500
	C10	−1.54382200	0.82814500	−0.76292100
	N11	−0.59307300	−0.20001700	−0.64757200
	N12	0.59309300	0.20049800	−0.64776100
	C13	1.54379900	−0.82777700	−0.76327000
	C14	2.91666100	−0.79952000	−0.30413200
	N15	3.49840900	−1.89089800	−0.74166000
	O16	2.53422700	−2.56539200	−1.48347400
	N17	1.34622000	−1.90424100	−1.48096800
	C18	3.60606800	0.19911300	0.49241600
	N19	4.94048900	0.37781200	0.48864000
	N20	5.13801500	1.36599300	1.36052800
	N21	3.97113100	1.75706800	1.87205000
	N22	2.98432700	1.03996500	1.33625200

SCF Done: E(B3LYP, cation) = −1144.0380666 a.u.

SCF Done: E(B3LYP, anion) = −1145.6620709 a.u.

Table S121: Salt 121

Cartesian coordinates

	Atom	X	Y	Z
Cation	C1	1.73873200	−1.43272900	0.06523700
	C2	2.78115000	−0.53199600	−0.09618600
	C3	2.58272300	0.83836400	−0.08296900
	C4	1.28876100	1.30925800	0.08646600
	C5	0.18564400	0.45946600	0.27650200
	C6	0.47216300	−0.91411900	0.27061800
	C7	−1.19631900	0.94965900	0.46912400
	N8	−2.13865200	0.28782100	−0.07950600
	N9	−3.42660700	0.69305100	0.12274000
	C10	−4.41899100	−0.09219600	−0.35406800
	N11	−5.68230800	0.33106500	−0.24703800
	N12	−4.11616000	−1.25828800	−0.90322900

	N13	−0.62189000	−1.88684500	0.53065600
	O14	−1.07337100	−1.92087600	1.65905600
	O15	−0.98524100	−2.57172500	−0.41467900
	N16	1.09775600	2.78355000	0.00382200
	O17	−0.03649000	3.18356500	−0.24463100
	O18	2.07313700	3.48293000	0.17674500
	N19	4.16165300	−1.06148900	−0.30003200
	O20	5.04731300	−0.23940300	−0.44505100
	O21	4.28523500	−2.27384100	−0.30307900
	H22	1.92627500	−2.49880100	0.04810600
	H23	3.40941000	1.52275400	−0.21875300
	H24	−1.36434000	1.83442100	1.07924800
	H25	−3.64335000	1.52660700	0.66309300
	H26	−6.45564100	−0.26488200	−0.50002900
	H27	−5.90473400	1.26755400	0.05394700
	H28	−4.81945600	−1.83307300	−1.34151400
	H29	−3.15254800	−1.57608700	−0.91123000
Anion	N1	0.67195600	2.05851800	0.00000000
	N2	−0.67221000	2.05845700	0.00000000
	N3	−1.11928500	0.81026500	0.00000000
	C4	0.00000000	0.07854900	0.00000000
	N5	1.11920100	0.81038500	0.00000000
	N6	0.00008100	−1.36159900	0.00000000
	O7	1.08603400	−1.94390800	0.00000000
	O8	−1.08580900	−1.94402600	0.00000000

SCF Done: E(B3LYP, cation) = −1144.0380666 a.u.

SCF Done: E(B3LYP, anion) = −462.3675711 a.u.

Table S122: Salt 122

Cartesian coordinates

	Atom	X	Y	Z
Cation	C1	1.73873200	−1.43272900	0.06523700
	C2	2.78115000	−0.53199600	−0.09618600
	C3	2.58272300	0.83836400	−0.08296900
	C4	1.28876100	1.30925800	0.08646600
	C5	0.18564400	0.45946600	0.27650200
	C6	0.47216300	−0.91411900	0.27061800
	C7	−1.19631900	0.94965900	0.46912400
	N8	−2.13865200	0.28782100	−0.07950600
	N9	−3.42660700	0.69305100	0.12274000
	C10	−4.41899100	−0.09219600	−0.35406800

	N11	−5.68230800	0.33106500	−0.24703800
	N12	−4.11616000	−1.25828800	−0.90322900
	N13	−0.62189000	−1.88684500	0.53065600
	O14	−1.07337100	−1.92087600	1.65905600
	O15	−0.98524100	−2.57172500	−0.41467900
	N16	1.09775600	2.78355000	0.00382200
	O17	−0.03649000	3.18356500	−0.24463100
	O18	2.07313700	3.48293000	0.17674500
	N19	4.16165300	−1.06148900	−0.30003200
	O20	5.04731300	−0.23940300	−0.44505100
	O21	4.28523500	−2.27384100	−0.30307900
	H22	1.92627500	−2.49880100	0.04810600
	H23	3.40941000	1.52275400	−0.21875300
	H24	−1.36434000	1.83442100	1.07924800
	H25	−3.64335000	1.52660700	0.66309300
	H26	−6.45564100	−0.26488200	−0.50002900
	H27	−5.90473400	1.26755400	0.05394700
	H28	−4.81945600	−1.83307300	−1.34151400
	H29	−3.15254800	−1.57608700	−0.91123000
Anion	C1	−0.32331000	0.09924700	0.00018400
	N2	0.18994600	1.32878300	0.00059600
	N3	1.49701900	1.17153100	−0.00033900
	N4	1.72208000	−0.18822300	−0.00011300
	N5	0.58879600	−0.88756000	0.00072200
	O6	2.87185800	−0.68467900	−0.00033400
	N7	−1.73407500	−0.14934000	−0.00034800
	O8	−2.11102300	−1.32486900	−0.00005500
	O9	−2.49914900	0.81932000	−0.00020200

SCF Done: E(B3LYP, cation) = −1144.0380666 a.u.

SCF Done: E(B3LYP, anion) = −537.5669456 a.u.

Table S123: Salt 123

Cartesian coordinates

	Atom	X	Y	Z
Cation	C1	1.73873200	−1.43272900	0.06523700
	C2	2.78115000	−0.53199600	−0.09618600
	C3	2.58272300	0.83836400	−0.08296900
	C4	1.28876100	1.30925800	0.08646600
	C5	0.18564400	0.45946600	0.27650200
	C6	0.47216300	−0.91411900	0.27061800
	C7	−1.19631900	0.94965900	0.46912400

	N8	−2.13865200	0.28782100	−0.07950600
	N9	−3.42660700	0.69305100	0.12274000
	C10	−4.41899100	−0.09219600	−0.35406800
	N11	−5.68230800	0.33106500	−0.24703800
	N12	−4.11616000	−1.25828800	−0.90322900
	N13	−0.62189000	−1.88684500	0.53065600
	O14	−1.07337100	−1.92087600	1.65905600
	O15	−0.98524100	−2.57172500	−0.41467900
	N16	1.09775600	2.78355000	0.00382200
	O17	−0.03649000	3.18356500	−0.24463100
	O18	2.07313700	3.48293000	0.17674500
	N19	4.16165300	−1.06148900	−0.30003200
	O20	5.04731300	−0.23940300	−0.44505100
	O21	4.28523500	−2.27384100	−0.30307900
	H22	1.92627500	−2.49880100	0.04810600
	H23	3.40941000	1.52275400	−0.21875300
	H24	−1.36434000	1.83442100	1.07924800
	H25	−3.64335000	1.52660700	0.66309300
	H26	−6.45564100	−0.26488200	−0.50002900
	H27	−5.90473400	1.26755400	0.05394700
	H28	−4.81945600	−1.83307300	−1.34151400
	H29	−3.15254800	−1.57608700	−0.91123000
Anion	N1	2.80157900	0.71401300	−0.00000900
	N2	3.00370900	−0.59546200	0.00005600
	N3	1.83247700	−1.24297800	−0.00000500
	C4	0.91587000	−0.26585200	0.00000000
	N5	1.48783600	0.95606100	0.00002600
	C6	−0.52956300	−0.40499200	−0.00001700
	N7	−1.21858900	−1.51344400	−0.00004500
	O8	−2.54597900	−1.14967400	−0.00008600
	N9	−2.68894200	0.24496000	−0.00001000
	C10	−1.46557700	0.70561100	0.00001400
	N11	−1.10914400	2.01489800	0.00007000
	H12	−0.10936200	2.20889500	0.00007000
	H13	−1.80967200	2.73356000	0.00007100

SCF Done: E(B3LYP, cation) = −1144.0380666 a.u.

SCF Done: E(B3LYP, anion) = −574.1182786 a.u.

Table S124: Salt 124

Cartesian coordinates

	Atom	X	Y	Z

Cation	C1	1.73873200	−1.43272900	0.06523700
	C2	2.78115000	−0.53199600	−0.09618600
	C3	2.58272300	0.83836400	−0.08296900
	C4	1.28876100	1.30925800	0.08646600
	C5	0.18564400	0.45946600	0.27650200
	C6	0.47216300	−0.91411900	0.27061800
	C7	−1.19631900	0.94965900	0.46912400
	N8	−2.13865200	0.28782100	−0.07950600
	N9	−3.42660700	0.69305100	0.12274000
	C10	−4.41899100	−0.09219600	−0.35406800
	N11	−5.68230800	0.33106500	−0.24703800
	N12	−4.11616000	−1.25828800	−0.90322900
	N13	−0.62189000	−1.88684500	0.53065600
	O14	−1.07337100	−1.92087600	1.65905600
	O15	−0.98524100	−2.57172500	−0.41467900
	N16	1.09775600	2.78355000	0.00382200
	O17	−0.03649000	3.18356500	−0.24463100
	O18	2.07313700	3.48293000	0.17674500
	N19	4.16165300	−1.06148900	−0.30003200
	O20	5.04731300	−0.23940300	−0.44505100
	O21	4.28523500	−2.27384100	−0.30307900
	H22	1.92627500	−2.49880100	0.04810600
	H23	3.40941000	1.52275400	−0.21875300
	H24	−1.36434000	1.83442100	1.07924800
	H25	−3.64335000	1.52660700	0.66309300
	H26	−6.45564100	−0.26488200	−0.50002900
	H27	−5.90473400	1.26755400	0.05394700
	H28	−4.81945600	−1.83307300	−1.34151400
	H29	−3.15254800	−1.57608700	−0.91123000
Anion	N1	0.00875500	−0.68767200	−0.00008400
	C2	−1.04271900	0.15278000	0.00006800
	C3	−0.60820500	1.49561500	−0.00001000
	N4	0.72848700	1.49980500	−0.00013900
	C5	1.02289200	0.17449800	0.00021400
	N6	2.39809700	−0.28461200	0.00067900
	O7	3.29154400	0.56477300	−0.00031100
	O8	2.60950000	−1.49786000	−0.00025100
	N9	−2.39909000	−0.30038300	0.00022400
	O10	−3.28335800	0.57377800	−0.00011300
	O11	−2.64065600	−1.50622800	−0.00012600
	H12	−1.20179300	2.39696700	0.00003300

SCF Done: E(B3LYP, cation) = −1144.0380666 a.u.

SCF Done: E(B3LYP, anion) = −634.8962734 a.u.

Table S125: Salt 125

Cartesian coordinates

	Atom	X	Y	Z
Cation	C1	1.73873200	−1.43272900	0.06523700
	C2	2.78115000	−0.53199600	−0.09618600
	C3	2.58272300	0.83836400	−0.08296900
	C4	1.28876100	1.30925800	0.08646600
	C5	0.18564400	0.45946600	0.27650200
	C6	0.47216300	−0.91411900	0.27061800
	C7	−1.19631900	0.94965900	0.46912400
	N8	−2.13865200	0.28782100	−0.07950600
	N9	−3.42660700	0.69305100	0.12274000
	C10	−4.41899100	−0.09219600	−0.35406800
	N11	−5.68230800	0.33106500	−0.24703800
	N12	−4.11616000	−1.25828800	−0.90322900
	N13	−0.62189000	−1.88684500	0.53065600
	O14	−1.07337100	−1.92087600	1.65905600
	O15	−0.98524100	−2.57172500	−0.41467900
	N16	1.09775600	2.78355000	0.00382200
	O17	−0.03649000	3.18356500	−0.24463100
	O18	2.07313700	3.48293000	0.17674500
	N19	4.16165300	−1.06148900	−0.30003200
	O20	5.04731300	−0.23940300	−0.44505100
	O21	4.28523500	−2.27384100	−0.30307900
	H22	1.92627500	−2.49880100	0.04810600
	H23	3.40941000	1.52275400	−0.21875300
	H24	−1.36434000	1.83442100	1.07924800
	H25	−3.64335000	1.52660700	0.66309300
	H26	−6.45564100	−0.26488200	−0.50002900
	H27	−5.90473400	1.26755400	0.05394700
	H28	−4.81945600	−1.83307300	−1.34151400
	H29	−3.15254800	−1.57608700	−0.91123000
Anion	N1	−1.81120700	1.72418200	0.00000000
	N2	−0.79668000	2.57503900	0.00000000
	N3	0.35873200	1.89717100	0.00000000
	C4	0.00000000	0.58544800	0.00000000
	N5	−1.34623900	0.45662100	0.00000000
	N6	1.04723100	−0.34007800	0.00000000
	N7	0.81628700	−1.62454700	0.00000000
	O8	1.85074100	−2.38952400	0.00000000

	O9	−0.33534800	−2.15190100	0.00000000

SCF Done: E(B3LYP, cation) = −1144.0380666 a.u.

SCF Done: E(B3LYP, anion) = −517.0427728 a.u.

Table S126: Salt 126

Cartesian coordinates

	Atom	X	Y	Z
Cation	O1	−2.41721000	−1.63070300	0.00001300
	N2	−3.21717600	−0.34953200	0.00001200
	C3	−2.31548800	0.62805900	−0.00001500
	C4	−1.03473300	0.00120200	−0.00000200
	N5	−1.12730300	−1.31188400	−0.00003000
	O6	−4.41855900	−0.42314100	0.00002200
	C7	−2.72749400	2.06173600	−0.00003100
	C8	0.25640000	0.67453000	0.00002500
	N9	1.34268400	−0.00020500	0.00000800
	N10	2.52825500	0.68461500	0.00001700
	C11	3.66751000	−0.03976900	−0.00000100
	N12	4.84602300	0.59214800	0.00000500
	N13	3.57842000	−1.36176800	−0.00002100
	H14	−3.81569700	2.12749100	−0.00041800
	H15	−2.35725500	2.58076800	0.88829000
	H16	−2.35660700	2.58092500	−0.88798800
	H17	0.24820400	1.76774100	0.00004600
	H18	2.56191000	1.70080000	0.00003800
	H19	5.71362000	0.07824100	−0.00000200
	H20	4.91099100	1.59823400	0.00001800
	H21	4.39380900	−1.95431400	−0.00003700
	H22	2.65367600	−1.77728600	−0.00002200
Anion	N1	0.67195600	2.05851800	0.00000000
	N2	−0.67221000	2.05845700	0.00000000
	N3	−1.11928500	0.81026500	0.00000000
	C4	0.00000000	0.07854900	0.00000000
	N5	1.11920100	0.81038500	0.00000000
	N6	0.00008100	−1.36159900	0.00000000
	O7	1.08603400	−1.94390800	0.00000000
	O8	−1.08580900	−1.94402600	0.00000000

SCF Done: E(B3LYP, cation) = −674.7075741 a.u.

SCF Done: E(B3LYP, anion) = −462.3675711 a.u.

Table S127: Salt 127

Cartesian coordinates

	Atom	X	Y	Z
Cation	O1	−2.41721000	−1.63070300	0.00001300
	N2	−3.21717600	−0.34953200	0.00001200
	C3	−2.31548800	0.62805900	−0.00001500
	C4	−1.03473300	0.00120200	−0.00000200
	N5	−1.12730300	−1.31188400	−0.00003000
	O6	−4.41855900	−0.42314100	0.00002200
	C7	−2.72749400	2.06173600	−0.00003100
	C8	0.25640000	0.67453000	0.00002500
	N9	1.34268400	−0.00020500	0.00000800
	N10	2.52825500	0.68461500	0.00001700
	C11	3.66751000	−0.03976900	−0.00000100
	N12	4.84602300	0.59214800	0.00000500
	N13	3.57842000	−1.36176800	−0.00002100
	H14	−3.81569700	2.12749100	−0.00041800
	H15	−2.35725500	2.58076800	0.88829000
	H16	−2.35660700	2.58092500	−0.88798800
	H17	0.24820400	1.76774100	0.00004600
	H18	2.56191000	1.70080000	0.00003800
	H19	5.71362000	0.07824100	−0.00000200
	H20	4.91099100	1.59823400	0.00001800
	H21	4.39380900	−1.95431400	−0.00003700
	H22	2.65367600	−1.77728600	−0.00002200
Anion	C1	−0.32331000	0.09924700	0.00018400
	N2	0.18994600	1.32878300	0.00059600
	N3	1.49701900	1.17153100	−0.00033900
	N4	1.72208000	−0.18822300	−0.00011300
	N5	0.58879600	−0.88756000	0.00072200
	O6	2.87185800	−0.68467900	−0.00033400
	N7	−1.73407500	−0.14934000	−0.00034800
	O8	−2.11102300	−1.32486900	−0.00005500
	O9	−2.49914900	0.81932000	−0.00020200

SCF Done: E(B3LYP, cation) = −674.7075741 a.u.

SCF Done: E(B3LYP, anion) = −537.5669456 a.u.

Table S128: Salt 128

Cartesian coordinates

	Atom	X	Y	Z

Cation	O1	−2.41721000	−1.63070300	0.00001300
	N2	−3.21717600	−0.34953200	0.00001200
	C3	−2.31548800	0.62805900	−0.00001500
	C4	−1.03473300	0.00120200	−0.00000200
	N5	−1.12730300	−1.31188400	−0.00003000
	O6	−4.41855900	−0.42314100	0.00002200
	C7	−2.72749400	2.06173600	−0.00003100
	C8	0.25640000	0.67453000	0.00002500
	N9	1.34268400	−0.00020500	0.00000800
	N10	2.52825500	0.68461500	0.00001700
	C11	3.66751000	−0.03976900	−0.00000100
	N12	4.84602300	0.59214800	0.00000500
	N13	3.57842000	−1.36176800	−0.00002100
	H14	−3.81569700	2.12749100	−0.00041800
	H15	−2.35725500	2.58076800	0.88829000
	H16	−2.35660700	2.58092500	−0.88798800
	H17	0.24820400	1.76774100	0.00004600
	H18	2.56191000	1.70080000	0.00003800
	H19	5.71362000	0.07824100	−0.00000200
	H20	4.91099100	1.59823400	0.00001800
	H21	4.39380900	−1.95431400	−0.00003700
	H22	2.65367600	−1.77728600	−0.00002200
Anion	N1	2.80157900	0.71401300	−0.00000900
	N2	3.00370900	−0.59546200	0.00005600
	N3	1.83247700	−1.24297800	−0.00000500
	C4	0.91587000	−0.26585200	0.00000000
	N5	1.48783600	0.95606100	0.00002600
	C6	−0.52956300	−0.40499200	−0.00001700
	N7	−1.21858900	−1.51344400	−0.00004500
	O8	−2.54597900	−1.14967400	−0.00008600
	N9	−2.68894200	0.24496000	−0.00001000
	C10	−1.46557700	0.70561100	0.00001400
	N11	−1.10914400	2.01489800	0.00007000
	H12	−0.10936200	2.20889500	0.00007000
	H13	−1.80967200	2.73356000	0.00007100

SCF Done: E(B3LYP, cation) = −674.7075741 a.u.

SCF Done: E(B3LYP, anion) = −574.1182786 a.u.

Table S129: Salt 129

Cartesian coordinates

	Atom	X	Y	Z

Cation	O1	−2.41721000	−1.63070300	0.00001300
	N2	−3.21717600	−0.34953200	0.00001200
	C3	−2.31548800	0.62805900	−0.00001500
	C4	−1.03473300	0.00120200	−0.00000200
	N5	−1.12730300	−1.31188400	−0.00003000
	O6	−4.41855900	−0.42314100	0.00002200
	C7	−2.72749400	2.06173600	−0.00003100
	C8	0.25640000	0.67453000	0.00002500
	N9	1.34268400	−0.00020500	0.00000800
	N10	2.52825500	0.68461500	0.00001700
	C11	3.66751000	−0.03976900	−0.00000100
	N12	4.84602300	0.59214800	0.00000500
	N13	3.57842000	−1.36176800	−0.00002100
	H14	−3.81569700	2.12749100	−0.00041800
	H15	−2.35725500	2.58076800	0.88829000
	H16	−2.35660700	2.58092500	−0.88798800
	H17	0.24820400	1.76774100	0.00004600
	H18	2.56191000	1.70080000	0.00003800
	H19	5.71362000	0.07824100	−0.00000200
	H20	4.91099100	1.59823400	0.00001800
	H21	4.39380900	−1.95431400	−0.00003700
	H22	2.65367600	−1.77728600	−0.00002200
Anion	C1	0.00000000	0.37938600	0.00000000
	N2	−0.42855600	1.63963700	0.00000000
	N3	−1.77005300	1.50771800	0.00000000
	N4	−2.09637900	0.22834300	0.00000000
	N5	−0.97985900	−0.52614600	0.00000000
	N6	1.38152500	0.07435500	0.00000000
	N7	1.71473300	−1.10429000	0.00000000
	N8	2.17859000	−2.14480400	0.00000000

SCF Done: E(B3LYP, cation) = −674.7075741 a.u.

SCF Done: E(B3LYP, anion) = −421.4323883 a.u.

Table S130: Salt 130

Cartesian coordinates

	Atom	X	Y	Z
Cation	O1	−2.41721000	−1.63070300	0.00001300
	N2	−3.21717600	−0.34953200	0.00001200
	C3	−2.31548800	0.62805900	−0.00001500
	C4	−1.03473300	0.00120200	−0.00000200
	N5	−1.12730300	−1.31188400	−0.00003000

	O6	−4.41855900	−0.42314100	0.00002200
	C7	−2.72749400	2.06173600	−0.00003100
	C8	0.25640000	0.67453000	0.00002500
	N9	1.34268400	−0.00020500	0.00000800
	N10	2.52825500	0.68461500	0.00001700
	C11	3.66751000	−0.03976900	−0.00000100
	N12	4.84602300	0.59214800	0.00000500
	N13	3.57842000	−1.36176800	−0.00002100
	H14	−3.81569700	2.12749100	−0.00041800
	H15	−2.35725500	2.58076800	0.88829000
	H16	−2.35660700	2.58092500	−0.88798800
	H17	0.24820400	1.76774100	0.00004600
	H18	2.56191000	1.70080000	0.00003800
	H19	5.71362000	0.07824100	−0.00000200
	H20	4.91099100	1.59823400	0.00001800
	H21	4.39380900	−1.95431400	−0.00003700
	H22	2.65367600	−1.77728600	−0.00002200
Anion	N1	0.00875500	−0.68767200	−0.00008400
	C2	−1.04271900	0.15278000	0.00006800
	C3	−0.60820500	1.49561500	−0.00001000
	N4	0.72848700	1.49980500	−0.00013900
	C5	1.02289200	0.17449800	0.00021400
	N6	2.39809700	−0.28461200	0.00067900
	O7	3.29154400	0.56477300	−0.00031100
	O8	2.60950000	−1.49786000	−0.00025100
	N9	−2.39909000	−0.30038300	0.00022400
	O10	−3.28335800	0.57377800	−0.00011300
	O11	−2.64065600	−1.50622800	−0.00012600
	H12	−1.20179300	2.39696700	0.00003300

SCF Done: E(B3LYP, cation) = −674.7075741 a.u.

SCF Done: E(B3LYP, anion) = −634.8962734 a.u.

Table S131: Salt 131

Cartesian coordinates

	Atom	X	Y	Z
Cation	O1	−2.41721000	−1.63070300	0.00001300
	N2	−3.21717600	−0.34953200	0.00001200
	C3	−2.31548800	0.62805900	−0.00001500
	C4	−1.03473300	0.00120200	−0.00000200
	N5	−1.12730300	−1.31188400	−0.00003000
	O6	−4.41855900	−0.42314100	0.00002200

	C7	−2.72749400	2.06173600	−0.00003100
	C8	0.25640000	0.67453000	0.00002500
	N9	1.34268400	−0.00020500	0.00000800
	N10	2.52825500	0.68461500	0.00001700
	C11	3.66751000	−0.03976900	−0.00000100
	N12	4.84602300	0.59214800	0.00000500
	N13	3.57842000	−1.36176800	−0.00002100
	H14	−3.81569700	2.12749100	−0.00041800
	H15	−2.35725500	2.58076800	0.88829000
	H16	−2.35660700	2.58092500	−0.88798800
	H17	0.24820400	1.76774100	0.00004600
	H18	2.56191000	1.70080000	0.00003800
	H19	5.71362000	0.07824100	−0.00000200
	H20	4.91099100	1.59823400	0.00001800
	H21	4.39380900	−1.95431400	−0.00003700
	H22	2.65367600	−1.77728600	−0.00002200
Anion	N1	−1.81120700	1.72418200	0.00000000
	N2	−0.79668000	2.57503900	0.00000000
	N3	0.35873200	1.89717100	0.00000000
	C4	0.00000000	0.58544800	0.00000000
	N5	−1.34623900	0.45662100	0.00000000
	N6	1.04723100	−0.34007800	0.00000000
	N7	0.81628700	−1.62454700	0.00000000
	O8	1.85074100	−2.38952400	0.00000000
	O9	−0.33534800	−2.15190100	0.00000000

SCF Done: E(B3LYP, cation) = −674.7075741 a.u.

SCF Done: E(B3LYP, anion) = −517.0427728 a.u.

Table S132: Salt 132

Cartesian coordinates

	Atom	X	Y	Z
Cation	O1	−2.41721000	−1.63070300	0.00001300
	N2	−3.21717600	−0.34953200	0.00001200
	C3	−2.31548800	0.62805900	−0.00001500
	C4	−1.03473300	0.00120200	−0.00000200
	N5	−1.12730300	−1.31188400	−0.00003000
	O6	−4.41855900	−0.42314100	0.00002200
	C7	−2.72749400	2.06173600	−0.00003100
	C8	0.25640000	0.67453000	0.00002500
	N9	1.34268400	−0.00020500	0.00000800
	N10	2.52825500	0.68461500	0.00001700

	C11	3.66751000	−0.03976900	−0.00000100
	N12	4.84602300	0.59214800	0.00000500
	N13	3.57842000	−1.36176800	−0.00002100
	H14	−3.81569700	2.12749100	−0.00041800
	H15	−2.35725500	2.58076800	0.88829000
	H16	−2.35660700	2.58092500	−0.88798800
	H17	0.24820400	1.76774100	0.00004600
	H18	2.56191000	1.70080000	0.00003800
	H19	5.71362000	0.07824100	−0.00000200
	H20	4.91099100	1.59823400	0.00001800
	H21	4.39380900	−1.95431400	−0.00003700
	H22	2.65367600	−1.77728600	−0.00002200
Anion	N1	−3.97109800	−1.75691000	1.87261300
	N2	−2.98430100	−1.03901700	1.33777000
	C3	−3.60589500	−0.19915300	0.49279600
	N4	−4.94010300	−0.37919200	0.48746600
	N5	−5.13769100	−1.36719600	1.35956200
	C6	−2.91663900	0.79966300	−0.30367000
	N7	−3.49857500	1.89095200	−0.74118100
	O8	−2.53466200	2.56555700	−1.48307000
	N9	−1.34649500	1.90459000	−1.48073500
	C10	−1.54382200	0.82814500	−0.76292100
	N11	−0.59307300	−0.20001700	−0.64757200
	N12	0.59309300	0.20049800	−0.64776100
	C13	1.54379900	−0.82777700	−0.76327000
	C14	2.91666100	−0.79952000	−0.30413200
	N15	3.49840900	−1.89089800	−0.74166000
	O16	2.53422700	−2.56539200	−1.48347400
	N17	1.34622000	−1.90424100	−1.48096800
	C18	3.60606800	0.19911300	0.49241600
	N19	4.94048900	0.37781200	0.48864000
	N20	5.13801500	1.36599300	1.36052800
	N21	3.97113100	1.75706800	1.87205000
	N22	2.98432700	1.03996500	1.33625200

SCF Done: E(B3LYP, cation) = −674.7075741 a.u.

SCF Done: E(B3LYP, anion) = −1145.6620709 a.u.

Table S133: Salt 133

Cartesian coordinates

	Atom	X	Y	Z

Cation	C1	1.07570400	0.33131400	0.00000000
	N2	0.00000000	1.09962100	0.00000000
	N3	−1.15290400	0.38196600	0.00000000
	C4	−0.75844100	−0.85881400	0.00000000
	N5	0.61778200	−0.92637500	0.00000000
	H6	2.10682100	0.65010800	0.00000000
	H7	−0.04266000	2.11387400	0.00000000
	H8	−1.40913300	−1.71930500	0.00000000
	H9	1.18725500	−1.76616000	0.00000000
Anion	C1	2.42471400	0.41201200	−0.00007800
	N2	3.23290400	1.47427100	−0.00000900
	O3	2.37576200	2.59302100	−0.00013600
	N4	1.07520000	2.23285000	−0.00028700
	C5	1.06077000	0.92735000	−0.00024600
	C6	−0.23033200	0.26992000	−0.00016200
	N7	−1.39553900	1.02161500	0.00012600
	C8	−2.45817000	0.17025000	0.00013100
	N9	−1.88027000	−1.05122400	−0.00011300
	N10	−0.50899900	−1.00467000	−0.00029600
	N11	2.62885900	−0.92121900	−0.00005700
	N12	3.91426000	−1.35535100	0.00015400
	O13	4.06331500	−2.58771100	0.00006300
	O14	4.87312900	−0.56358600	0.00041300
	N15	−3.70722200	0.63048100	0.00038200
	N16	−4.69342000	−0.30937700	0.00009400
	O17	−5.84329200	0.11397400	0.00035900
	O18	−4.41845100	−1.53194000	−0.00047200
	H19	−1.45036500	2.02949400	0.00066300
	H20	−2.39564200	−1.91837100	−0.00030200

SCF Done: E(B3LYP, cation) = −242.6694056 a.u.

SCF Done: E(B3LYP, anion) = −1022.5658661 a.u.

Table S134: Salt 134

Cartesian coordinates

	Atom	X	Y	Z
Cation	N1	−0.00001200	0.00000000	0.00000000
	H2	0.09263200	0.83857300	−0.58434300
	H3	0.10779100	−0.83730900	−0.58355400
	H4	0.72571300	0.00690600	0.72562600
	H5	−0.92605200	−0.00816900	0.44227100
Anion	C1	−2.38294800	0.39604700	−0.01685100

	N2	−3.32317700	1.32962300	−0.03254900
	O3	−2.63382800	2.54841200	0.08305200
	N4	−1.27848000	2.35454600	0.17855600
	C5	−1.08980200	1.07043700	0.12410100
	C6	0.27248800	0.51275800	0.20063200
	N7	1.28684600	0.93030500	−0.60510300
	C8	2.34233900	0.22799500	−0.17034900
	N9	1.92983900	−0.56566300	0.85814000
	N10	0.60795400	−0.39556600	1.10898700
	N11	−2.43177200	−0.96073300	−0.15608400
	N12	−3.63060400	−1.54669800	−0.22422100
	O13	−3.62845600	−2.78571600	−0.42360200
	O14	−4.70442300	−0.91500400	−0.08805200
	N15	3.58741400	0.36057000	−0.73404400
	N16	4.57724700	−0.34999700	−0.22622600
	O17	5.70896300	−0.21273000	−0.74683100
	O18	4.42558700	−1.15195700	0.75550100
	H19	2.52792100	−1.20217800	1.36206300

SCF Done: E(B3LYP, cation) = −56.9203571 a.u.

SCF Done: E(B3LYP, anion) = −1021.9625241 a.u.

Table S135: Salt 135

Cartesian coordinates

	Atom	X	Y	Z
Cation	N1	0.65541700	−0.00411800	0.00005100
	N2	−0.75045600	−0.00232700	−0.00003900
	H3	0.99718400	0.96411800	−0.00012100
	H4	1.05415400	−0.45557600	0.83700000
	H5	1.05425800	−0.45589200	−0.83667700
	H6	−1.21844400	−0.89213100	−0.00011000
	H7	−1.22187800	0.88459500	−0.00017400
Anion	C1	−2.38294800	0.39604700	−0.01685100
	N2	−3.32317700	1.32962300	−0.03254900
	O3	−2.63382800	2.54841200	0.08305200
	N4	−1.27848000	2.35454600	0.17855600
	C5	−1.08980200	1.07043700	0.12410100
	C6	0.27248800	0.51275800	0.20063200
	N7	1.28684600	0.93030500	−0.60510300
	C8	2.34233900	0.22799500	−0.17034900
	N9	1.92983900	−0.56566300	0.85814000
	N10	0.60795400	−0.39556600	1.10898700

	N11	−2.43177200	−0.96073300	−0.15608400
	N12	−3.63060400	−1.54669800	−0.22422100
	O13	−3.62845600	−2.78571600	−0.42360200
	O14	−4.70442300	−0.91500400	−0.08805200
	N15	3.58741400	0.36057000	−0.73404400
	N16	4.57724700	−0.34999700	−0.22622600
	O17	5.70896300	−0.21273000	−0.74683100
	O18	4.42558700	−1.15195700	0.75550100
	H19	2.52792100	−1.20217800	1.36206300

SCF Done: E(B3LYP, cation) = −112.2418137 a.u.

SCF Done: E(B3LYP, anion) = −1021.9625241 a.u.

Table S136: Salt 136

Cartesian coordinates

	Atom	X	Y	Z
Cation	N1	0.62157632	0.00638917	0.00006179
	O2	−0.73539340	0.09712580	−0.00012371
	H3	1.01706940	0.92485812	−0.00007138
	H4	0.92261491	−0.48604256	0.81669314
	H5	0.92281651	−0.48636615	−0.81629998
	H6	−1.11506675	−0.78460439	0.00000414
Anion	C1	−2.38294800	0.39604700	−0.01685100
	N2	−3.32317700	1.32962300	−0.03254900
	O3	−2.63382800	2.54841200	0.08305200
	N4	−1.27848000	2.35454600	0.17855600
	C5	−1.08980200	1.07043700	0.12410100
	C6	0.27248800	0.51275800	0.20063200
	N7	1.28684600	0.93030500	−0.60510300
	C8	2.34233900	0.22799500	−0.17034900
	N9	1.92983900	−0.56566300	0.85814000
	N10	0.60795400	−0.39556600	1.10898700
	N11	−2.43177200	−0.96073300	−0.15608400
	N12	−3.63060400	−1.54669800	−0.22422100
	O13	−3.62845600	−2.78571600	−0.42360200
	O14	−4.70442300	−0.91500400	−0.08805200
	N15	3.58741400	0.36057000	−0.73404400
	N16	4.57724700	−0.34999700	−0.22622600
	O17	5.70896300	−0.21273000	−0.74683100
	O18	4.42558700	−1.15195700	0.75550100
	H19	2.52792100	−1.20217800	1.36206300

SCF Done: E(B3LYP, cation) = −132.0865145 a.u.

SCF Done: E(B3LYP, anion) = −1021.9625241 a.u.

Table S137: Salt 137

Cartesian coordinates

	Atom	X	Y	Z
Cation	N1	0.01505600	1.33482600	−0.00016400
	C2	0.00005600	0.00000600	0.00002800
	N3	1.14853800	−0.68050900	0.00012700
	N4	−1.16365100	−0.65433800	−0.00009900
	H5	0.86148500	1.85114100	−0.18670200
	H6	−0.81953400	1.86956400	0.18784900
	H7	2.02923800	−0.22498500	0.18640200
	H8	1.17252800	−1.67159400	−0.18697800
	H9	−1.20975100	−1.64457800	0.18733500
	H10	−2.03389600	−0.17943500	−0.18712000
Anion	C1	−2.38294800	0.39604700	−0.01685100
	N2	−3.32317700	1.32962300	−0.03254900
	O3	−2.63382800	2.54841200	0.08305200
	N4	−1.27848000	2.35454600	0.17855600
	C5	−1.08980200	1.07043700	0.12410100
	C6	0.27248800	0.51275800	0.20063200
	N7	1.28684600	0.93030500	−0.60510300
	C8	2.34233900	0.22799500	−0.17034900
	N9	1.92983900	−0.56566300	0.85814000
	N10	0.60795400	−0.39556600	1.10898700
	N11	−2.43177200	−0.96073300	−0.15608400
	N12	−3.63060400	−1.54669800	−0.22422100
	O13	−3.62845600	−2.78571600	−0.42360200
	O14	−4.70442300	−0.91500400	−0.08805200
	N15	3.58741400	0.36057000	−0.73404400
	N16	4.57724700	−0.34999700	−0.22622600
	O17	5.70896300	−0.21273000	−0.74683100
	O18	4.42558700	−1.15195700	0.75550100
	H19	2.52792100	−1.20217800	1.36206300

SCF Done: E(B3LYP, cation) = −205.8356358 a.u.

SCF Done: E(B3LYP, anion) = −1021.9625241 a.u.

Table S138: Salt 138

Cartesian coordinates

	Atom	X	Y	Z
Cation	N1	−0.71309600	1.63579100	0.00000000
	C2	0.00000000	0.49075400	0.00000000
	N3	1.34087600	0.55400700	0.00000000
	N4	−0.63397000	−0.66964200	0.00000000
	N5	−0.05044100	−1.92877000	0.00000000
	H6	−1.72036800	1.63935700	0.00000000
	H7	−0.25478500	2.53240900	0.00000000
	H8	1.92947100	−0.26070500	0.00000000
	H9	1.81369600	1.44397200	0.00000000
	H10	−1.64306300	−0.68139000	0.00000000
	H11	−0.67178300	−2.71571000	0.00000000
	H12	0.94325200	−2.04216900	0.00000000
Anion	C1	−2.38294800	0.39604700	−0.01685100
	N2	−3.32317700	1.32962300	−0.03254900
	O3	−2.63382800	2.54841200	0.08305200
	N4	−1.27848000	2.35454600	0.17855600
	C5	−1.08980200	1.07043700	0.12410100
	C6	0.27248800	0.51275800	0.20063200
	N7	1.28684600	0.93030500	−0.60510300
	C8	2.34233900	0.22799500	−0.17034900
	N9	1.92983900	−0.56566300	0.85814000
	N10	0.60795400	−0.39556600	1.10898700
	N11	−2.43177200	−0.96073300	−0.15608400
	N12	−3.63060400	−1.54669800	−0.22422100
	O13	−3.62845600	−2.78571600	−0.42360200
	O14	−4.70442300	−0.91500400	−0.08805200
	N15	3.58741400	0.36057000	−0.73404400
	N16	4.57724700	−0.34999700	−0.22622600
	O17	5.70896300	−0.21273000	−0.74683100
	O18	4.42558700	−1.15195700	0.75550100
	H19	2.52792100	−1.20217800	1.36206300

SCF Done: E(B3LYP, cation) = −261.143769 a.u.

SCF Done: E(B3LYP, anion) = −1021.9625241 a.u.

Table S139: Salt 139

Cartesian coordinates

	Atom	X	Y	Z
Cation	N1	−1.23126100	−0.61392200	−0.00005700
	C2	−0.06944600	0.01029100	−0.00002200
	N3	−2.41564800	0.10939600	−0.00009600

	N4	1.12200500	−0.76734500	0.00008400
	N5	2.24162600	0.08278500	0.00006100
	O6	0.09192400	1.23885400	0.00003400
	H7	−1.31636200	−1.61920200	−0.00004200
	H8	−3.27674900	−0.40124900	−0.00008900
	H9	−2.33833700	1.11046000	−0.00012000
	H10	1.25457300	−1.76698500	0.00002500
	H11	1.70267300	1.03124200	0.00000300
	H12	2.81920300	−0.00159200	0.84339400
	H13	2.81922300	−0.00165200	−0.84325300
Anion	C1	−2.38294800	0.39604700	−0.01685100
	N2	−3.32317700	1.32962300	−0.03254900
	O3	−2.63382800	2.54841200	0.08305200
	N4	−1.27848000	2.35454600	0.17855600
	C5	−1.08980200	1.07043700	0.12410100
	C6	0.27248800	0.51275800	0.20063200
	N7	1.28684600	0.93030500	−0.60510300
	C8	2.34233900	0.22799500	−0.17034900
	N9	1.92983900	−0.56566300	0.85814000
	N10	0.60795400	−0.39556600	1.10898700
	N11	−2.43177200	−0.96073300	−0.15608400
	N12	−3.63060400	−1.54669800	−0.22422100
	O13	−3.62845600	−2.78571600	−0.42360200
	O14	−4.70442300	−0.91500400	−0.08805200
	N15	3.58741400	0.36057000	−0.73404400
	N16	4.57724700	−0.34999700	−0.22622600
	O17	5.70896300	−0.21273000	−0.74683100
	O18	4.42558700	−1.15195700	0.75550100
	H19	2.52792100	−1.20217800	1.36206300

SCF Done: E(B3LYP, cation) = −336.3373235 a.u.

SCF Done: E(B3LYP, anion) = −1021.9625241 a.u.

Table S140: Salt 140

Cartesian coordinates

	Atom	X	Y	Z
Cation	C1	1.07570400	0.33131400	0.00000000
	N2	0.00000000	1.09962100	0.00000000
	N3	−1.15290400	0.38196600	0.00000000
	C4	−0.75844100	−0.85881400	0.00000000
	N5	0.61778200	−0.92637500	0.00000000
	H6	2.10682100	0.65010800	0.00000000

	H7	−0.04266000	2.11387400	0.00000000
	H8	−1.40913300	−1.71930500	0.00000000
	H9	1.18725500	−1.76616000	0.00000000
Anion	C1	−2.38294800	0.39604700	−0.01685100
	N2	−3.32317700	1.32962300	−0.03254900
	O3	−2.63382800	2.54841200	0.08305200
	N4	−1.27848000	2.35454600	0.17855600
	C5	−1.08980200	1.07043700	0.12410100
	C6	0.27248800	0.51275800	0.20063200
	N7	1.28684600	0.93030500	−0.60510300
	C8	2.34233900	0.22799500	−0.17034900
	N9	1.92983900	−0.56566300	0.85814000
	N10	0.60795400	−0.39556600	1.10898700
	N11	−2.43177200	−0.96073300	−0.15608400
	N12	−3.63060400	−1.54669800	−0.22422100
	O13	−3.62845600	−2.78571600	−0.42360200
	O14	−4.70442300	−0.91500400	−0.08805200
	N15	3.58741400	0.36057000	−0.73404400
	N16	4.57724700	−0.34999700	−0.22622600
	O17	5.70896300	−0.21273000	−0.74683100
	O18	4.42558700	−1.15195700	0.75550100
	H19	2.52792100	−1.20217800	1.36206300

SCF Done: E(B3LYP, cation) = −242.6694056 a.u.

SCF Done: E(B3LYP, anion) = −1021.9625241 a.u.

Table S141: Salt 141

Cartesian coordinates

	Atom	X	Y	Z
Cation	C1	−1.08228900	−0.13710400	0.00000000
	N2	−0.61573600	−1.38850300	0.00000000
	N3	0.72573800	−1.43573300	0.00000000
	C4	1.10022700	−0.18529000	0.00000000
	N5	0.00000000	0.65115400	0.00000000
	N6	0.02662600	2.01061000	0.00000000
	H7	−2.11338600	0.17676100	0.00000000
	H8	−1.15210300	−2.24869500	0.00000000
	H9	2.12050200	0.16391300	0.00000000
	H10	0.92340300	2.46433500	0.00000000
	H11	−0.84244300	2.51536000	0.00000000
Anion	C1	−2.38294800	0.39604700	−0.01685100
	N2	−3.32317700	1.32962300	−0.03254900

	O3	−2.63382800	2.54841200	0.08305200
	N4	−1.27848000	2.35454600	0.17855600
	C5	−1.08980200	1.07043700	0.12410100
	C6	0.27248800	0.51275800	0.20063200
	N7	1.28684600	0.93030500	−0.60510300
	C8	2.34233900	0.22799500	−0.17034900
	N9	1.92983900	−0.56566300	0.85814000
	N10	0.60795400	−0.39556600	1.10898700
	N11	−2.43177200	−0.96073300	−0.15608400
	N12	−3.63060400	−1.54669800	−0.22422100
	O13	−3.62845600	−2.78571600	−0.42360200
	O14	−4.70442300	−0.91500400	−0.08805200
	N15	3.58741400	0.36057000	−0.73404400
	N16	4.57724700	−0.34999700	−0.22622600
	O17	5.70896300	−0.21273000	−0.74683100
	O18	4.42558700	−1.15195700	0.75550100
	H19	2.52792100	−1.20217800	1.36206300

SCF Done: E(B3LYP, cation) = −297.9953294 a.u.

SCF Done: E(B3LYP, anion) = −1021.9625241 a.u.

Table S142: Salt 142

Cartesian coordinates

	Atom	X	Y	Z
Cation	N1	−1.14623600	0.03333100	0.00000000
	N2	−0.77808300	−1.27764000	0.00000000
	C3	0.52291600	−1.45275200	0.00000000
	N4	1.05687000	−0.21481200	0.00000000
	C5	0.00000000	0.68776000	0.00000000
	N6	0.13955500	2.01763800	0.00000000
	H7	−1.49806000	−1.99128000	0.00000000
	H8	1.06053600	−2.38757900	0.00000000
	H9	2.04787200	−0.00691200	0.00000000
	H10	−0.68962500	2.59303300	0.00000000
	H11	1.03703700	2.47307500	0.00000000
Anion	C1	−2.38294800	0.39604700	−0.01685100
	N2	−3.32317700	1.32962300	−0.03254900
	O3	−2.63382800	2.54841200	0.08305200
	N4	−1.27848000	2.35454600	0.17855600
	C5	−1.08980200	1.07043700	0.12410100
	C6	0.27248800	0.51275800	0.20063200
	N7	1.28684600	0.93030500	−0.60510300

	C8	2.34233900	0.22799500	−0.17034900
	N9	1.92983900	−0.56566300	0.85814000
	N10	0.60795400	−0.39556600	1.10898700
	N11	−2.43177200	−0.96073300	−0.15608400
	N12	−3.63060400	−1.54669800	−0.22422100
	O13	−3.62845600	−2.78571600	−0.42360200
	O14	−4.70442300	−0.91500400	−0.08805200
	N15	3.58741400	0.36057000	−0.73404400
	N16	4.57724700	−0.34999700	−0.22622600
	O17	5.70896300	−0.21273000	−0.74683100
	O18	4.42558700	−1.15195700	0.75550100
	H19	2.52792100	−1.20217800	1.36206300

SCF Done: E(B3LYP, cation) = −298.0617301 a.u.

SCF Done: E(B3LYP, anion) = −1021.9625241 a.u.

Table S143: Salt 143

Cartesian coordinates

	Atom	X	Y	Z
Cation	N1	−0.00001200	0.00000000	0.00000000
	H2	0.09263200	0.83857300	−0.58434300
	H3	0.10779100	−0.83730900	−0.58355400
	H4	0.72571300	0.00690600	0.72562600
	H5	−0.92605200	−0.00816900	0.44227100
Anion	C1	2.58189000	0.24147100	−0.01939400
	N2	3.81210100	−0.14870400	−0.10559200
	O3	3.73524400	−1.51122400	−0.17745200
	N4	2.42720300	−1.94786100	−0.14365200
	C5	1.68288300	−0.87493300	−0.03591600
	C6	0.23122400	−0.84078900	0.01650100
	N7	−0.43311200	0.32435600	−0.16591300
	C8	−1.72520800	−0.03870000	−0.06514500
	N9	−1.75713900	−1.38974900	0.17040100
	N10	−0.51462900	−1.91799900	0.22761700
	N11	2.30210000	1.67723600	0.13147700
	O12	2.00901300	2.04761300	1.25325100
	O13	2.42502300	2.37005200	−0.86162400
	N14	−2.74062100	0.85126100	−0.19553800
	N15	−3.98632500	0.38456900	−0.07364800
	O16	−4.90720200	1.20302900	−0.20020800
	O17	−4.23468800	−0.83928700	0.16204000
	H18	−2.61090500	−1.91550400	0.29959600

SCF Done: E(B3LYP, cation) = −56.9203571 a.u.

SCF Done: E(B3LYP, anion) = −967.2011174 a.u.

Table S144: Salt 144

Cartesian coordinates

	Atom	X	Y	Z
Cation	N1	0.65541700	−0.00411800	0.00005100
	N2	−0.75045600	−0.00232700	−0.00003900
	H3	0.99718400	0.96411800	−0.00012100
	H4	1.05415400	−0.45557600	0.83700000
	H5	1.05425800	−0.45589200	−0.83667700
	H6	−1.21844400	−0.89213100	−0.00011000
	H7	−1.22187800	0.88459500	−0.00017400
Anion	C1	2.58189000	0.24147100	−0.01939400
	N2	3.81210100	−0.14870400	−0.10559200
	O3	3.73524400	−1.51122400	−0.17745200
	N4	2.42720300	−1.94786100	−0.14365200
	C5	1.68288300	−0.87493300	−0.03591600
	C6	0.23122400	−0.84078900	0.01650100
	N7	−0.43311200	0.32435600	−0.16591300
	C8	−1.72520800	−0.03870000	−0.06514500
	N9	−1.75713900	−1.38974900	0.17040100
	N10	−0.51462900	−1.91799900	0.22761700
	N11	2.30210000	1.67723600	0.13147700
	O12	2.00901300	2.04761300	1.25325100
	O13	2.42502300	2.37005200	−0.86162400
	N14	−2.74062100	0.85126100	−0.19553800
	N15	−3.98632500	0.38456900	−0.07364800
	O16	−4.90720200	1.20302900	−0.20020800
	O17	−4.23468800	−0.83928700	0.16204000
	H18	−2.61090500	−1.91550400	0.29959600

SCF Done: E(B3LYP, cation) = −112.2418137 a.u.

SCF Done: E(B3LYP, anion) = −967.2011174 a.u.

Table S145: Salt 145

Cartesian coordinates

	Atom	X	Y	Z
Cation	N1	0.62157632	0.00638917	0.00006179
	O2	−0.73539340	0.09712580	−0.00012371

	H3	1.01706940	0.92485812	−0.00007138
	H4	0.92261491	−0.48604256	0.81669314
	H5	0.92281651	−0.48636615	−0.81629998
	H6	−1.11506675	−0.78460439	0.00000414
Anion	C1	2.58189000	0.24147100	−0.01939400
	N2	3.81210100	−0.14870400	−0.10559200
	O3	3.73524400	−1.51122400	−0.17745200
	N4	2.42720300	−1.94786100	−0.14365200
	C5	1.68288300	−0.87493300	−0.03591600
	C6	0.23122400	−0.84078900	0.01650100
	N7	−0.43311200	0.32435600	−0.16591300
	C8	−1.72520800	−0.03870000	−0.06514500
	N9	−1.75713900	−1.38974900	0.17040100
	N10	−0.51462900	−1.91799900	0.22761700
	N11	2.30210000	1.67723600	0.13147700
	O12	2.00901300	2.04761300	1.25325100
	O13	2.42502300	2.37005200	−0.86162400
	N14	−2.74062100	0.85126100	−0.19553800
	N15	−3.98632500	0.38456900	−0.07364800
	O16	−4.90720200	1.20302900	−0.20020800
	O17	−4.23468800	−0.83928700	0.16204000
	H18	−2.61090500	−1.91550400	0.29959600

SCF Done: E(B3LYP, cation) = −132.0865145 a.u.

SCF Done: E(B3LYP, anion) = −967.2011174 a.u.

Table S146: Salt 146

Cartesian coordinates

	Atom	X	Y	Z
Cation	N1	0.01505600	1.33482600	−0.00016400
	C2	0.00005600	0.00000600	0.00002800
	N3	1.14853800	−0.68050900	0.00012700
	N4	−1.16365100	−0.65433800	−0.00009900
	H5	0.86148500	1.85114100	−0.18670200
	H6	−0.81953400	1.86956400	0.18784900
	H7	2.02923800	−0.22498500	0.18640200
	H8	1.17252800	−1.67159400	−0.18697800
	H9	−1.20975100	−1.64457800	0.18733500
	H10	−2.03389600	−0.17943500	−0.18712000
Anion	C1	2.58189000	0.24147100	−0.01939400
	N2	3.81210100	−0.14870400	−0.10559200
	O3	3.73524400	−1.51122400	−0.17745200

	N4	2.42720300	−1.94786100	−0.14365200
	C5	1.68288300	−0.87493300	−0.03591600
	C6	0.23122400	−0.84078900	0.01650100
	N7	−0.43311200	0.32435600	−0.16591300
	C8	−1.72520800	−0.03870000	−0.06514500
	N9	−1.75713900	−1.38974900	0.17040100
	N10	−0.51462900	−1.91799900	0.22761700
	N11	2.30210000	1.67723600	0.13147700
	O12	2.00901300	2.04761300	1.25325100
	O13	2.42502300	2.37005200	−0.86162400
	N14	−2.74062100	0.85126100	−0.19553800
	N15	−3.98632500	0.38456900	−0.07364800
	O16	−4.90720200	1.20302900	−0.20020800
	O17	−4.23468800	−0.83928700	0.16204000
	H18	−2.61090500	−1.91550400	0.29959600

SCF Done: E(B3LYP, cation) = −205.8356358 a.u.

SCF Done: E(B3LYP, anion) = −967.2011174 a.u.

Table S147: Salt 147

Cartesian coordinates

	Atom	X	Y	Z
Cation	N1	−0.71309600	1.63579100	0.00000000
	C2	0.00000000	0.49075400	0.00000000
	N3	1.34087600	0.55400700	0.00000000
	N4	−0.63397000	−0.66964200	0.00000000
	N5	−0.05044100	−1.92877000	0.00000000
	H6	−1.72036800	1.63935700	0.00000000
	H7	−0.25478500	2.53240900	0.00000000
	H8	1.92947100	−0.26070500	0.00000000
	H9	1.81369600	1.44397200	0.00000000
	H10	−1.64306300	−0.68139000	0.00000000
	H11	−0.67178300	−2.71571000	0.00000000
	H12	0.94325200	−2.04216900	0.00000000
Anion	C1	2.58189000	0.24147100	−0.01939400
	N2	3.81210100	−0.14870400	−0.10559200
	O3	3.73524400	−1.51122400	−0.17745200
	N4	2.42720300	−1.94786100	−0.14365200
	C5	1.68288300	−0.87493300	−0.03591600
	C6	0.23122400	−0.84078900	0.01650100
	N7	−0.43311200	0.32435600	−0.16591300
	C8	−1.72520800	−0.03870000	−0.06514500

	N9	−1.75713900	−1.38974900	0.17040100
	N10	−0.51462900	−1.91799900	0.22761700
	N11	2.30210000	1.67723600	0.13147700
	O12	2.00901300	2.04761300	1.25325100
	O13	2.42502300	2.37005200	−0.86162400
	N14	−2.74062100	0.85126100	−0.19553800
	N15	−3.98632500	0.38456900	−0.07364800
	O16	−4.90720200	1.20302900	−0.20020800
	O17	−4.23468800	−0.83928700	0.16204000
	H18	−2.61090500	−1.91550400	0.29959600

SCF Done: E(B3LYP, cation) = −261.143769 a.u.

SCF Done: E(B3LYP, anion) = −967.2011174 a.u.

Table S148: Salt 148

Cartesian coordinates

	Atom	X	Y	Z
Cation	N1	−1.23126100	−0.61392200	−0.00005700
	C2	−0.06944600	0.01029100	−0.00002200
	N3	−2.41564800	0.10939600	−0.00009600
	N4	1.12200500	−0.76734500	0.00008400
	N5	2.24162600	0.08278500	0.00006100
	O6	0.09192400	1.23885400	0.00003400
	H7	−1.31636200	−1.61920200	−0.00004200
	H8	−3.27674900	−0.40124900	−0.00008900
	H9	−2.33833700	1.11046000	−0.00012000
	H10	1.25457300	−1.76698500	0.00002500
	H11	1.70267300	1.03124200	0.00000300
	H12	2.81920300	−0.00159200	0.84339400
	H13	2.81922300	−0.00165200	−0.84325300
Anion	C1	2.58189000	0.24147100	−0.01939400
	N2	3.81210100	−0.14870400	−0.10559200
	O3	3.73524400	−1.51122400	−0.17745200
	N4	2.42720300	−1.94786100	−0.14365200
	C5	1.68288300	−0.87493300	−0.03591600
	C6	0.23122400	−0.84078900	0.01650100
	N7	−0.43311200	0.32435600	−0.16591300
	C8	−1.72520800	−0.03870000	−0.06514500
	N9	−1.75713900	−1.38974900	0.17040100
	N10	−0.51462900	−1.91799900	0.22761700
	N11	2.30210000	1.67723600	0.13147700
	O12	2.00901300	2.04761300	1.25325100

	O13	2.42502300	2.37005200	−0.86162400
	N14	−2.74062100	0.85126100	−0.19553800
	N15	−3.98632500	0.38456900	−0.07364800
	O16	−4.90720200	1.20302900	−0.20020800
	O17	−4.23468800	−0.83928700	0.16204000
	H18	−2.61090500	−1.91550400	0.29959600

SCF Done: E(B3LYP, cation) = −336.3373235 a.u.

SCF Done: E(B3LYP, anion) = −967.2011174 a.u.

Table S149: Salt 149

Cartesian coordinates

	Atom	X	Y	Z
Cation	C1	1.07570400	0.33131400	0.00000000
	N2	0.00000000	1.09962100	0.00000000
	N3	−1.15290400	0.38196600	0.00000000
	C4	−0.75844100	−0.85881400	0.00000000
	N5	0.61778200	−0.92637500	0.00000000
	H6	2.10682100	0.65010800	0.00000000
	H7	−0.04266000	2.11387400	0.00000000
	H8	−1.40913300	−1.71930500	0.00000000
	H9	1.18725500	−1.76616000	0.00000000
Anion	C1	2.58189000	0.24147100	−0.01939400
	N2	3.81210100	−0.14870400	−0.10559200
	O3	3.73524400	−1.51122400	−0.17745200
	N4	2.42720300	−1.94786100	−0.14365200
	C5	1.68288300	−0.87493300	−0.03591600
	C6	0.23122400	−0.84078900	0.01650100
	N7	−0.43311200	0.32435600	−0.16591300
	C8	−1.72520800	−0.03870000	−0.06514500
	N9	−1.75713900	−1.38974900	0.17040100
	N10	−0.51462900	−1.91799900	0.22761700
	N11	2.30210000	1.67723600	0.13147700
	O12	2.00901300	2.04761300	1.25325100
	O13	2.42502300	2.37005200	−0.86162400
	N14	−2.74062100	0.85126100	−0.19553800
	N15	−3.98632500	0.38456900	−0.07364800
	O16	−4.90720200	1.20302900	−0.20020800
	O17	−4.23468800	−0.83928700	0.16204000
	H18	−2.61090500	−1.91550400	0.29959600

SCF Done: E(B3LYP, cation) = −242.6694056 a.u.

SCF Done: E(B3LYP, anion) = −967.2011174 a.u.

Table S150: Salt 150

Cartesian coordinates

	Atom	X	Y	Z
Cation	C1	−1.08228900	−0.13710400	0.00000000
	N2	−0.61573600	−1.38850300	0.00000000
	N3	0.72573800	−1.43573300	0.00000000
	C4	1.10022700	−0.18529000	0.00000000
	N5	0.00000000	0.65115400	0.00000000
	N6	0.02662600	2.01061000	0.00000000
	H7	−2.11338600	0.17676100	0.00000000
	H8	−1.15210300	−2.24869500	0.00000000
	H9	2.12050200	0.16391300	0.00000000
	H10	0.92340300	2.46433500	0.00000000
	H11	−0.84244300	2.51536000	0.00000000
Anion	C1	2.58189000	0.24147100	−0.01939400
	N2	3.81210100	−0.14870400	−0.10559200
	O3	3.73524400	−1.51122400	−0.17745200
	N4	2.42720300	−1.94786100	−0.14365200
	C5	1.68288300	−0.87493300	−0.03591600
	C6	0.23122400	−0.84078900	0.01650100
	N7	−0.43311200	0.32435600	−0.16591300
	C8	−1.72520800	−0.03870000	−0.06514500
	N9	−1.75713900	−1.38974900	0.17040100
	N10	−0.51462900	−1.91799900	0.22761700
	N11	2.30210000	1.67723600	0.13147700
	O12	2.00901300	2.04761300	1.25325100
	O13	2.42502300	2.37005200	−0.86162400
	N14	−2.74062100	0.85126100	−0.19553800
	N15	−3.98632500	0.38456900	−0.07364800
	O16	−4.90720200	1.20302900	−0.20020800
	O17	−4.23468800	−0.83928700	0.16204000
	H18	−2.61090500	−1.91550400	0.29959600

SCF Done: E(B3LYP, cation) = −297.9953294 a.u.

SCF Done: E(B3LYP, anion) = −967.2011174 a.u.

Table S151: Salt 151

Cartesian coordinates

	Atom	X	Y	Z

Cation	N1	−1.14623600	0.03333100	0.00000000
	N2	−0.77808300	−1.27764000	0.00000000
	C3	0.52291600	−1.45275200	0.00000000
	N4	1.05687000	−0.21481200	0.00000000
	C5	0.00000000	0.68776000	0.00000000
	N6	0.13955500	2.01763800	0.00000000
	H7	−1.49806000	−1.99128000	0.00000000
	H8	1.06053600	−2.38757900	0.00000000
	H9	2.04787200	−0.00691200	0.00000000
	H10	−0.68962500	2.59303300	0.00000000
	H11	1.03703700	2.47307500	0.00000000
Anion	C1	2.58189000	0.24147100	−0.01939400
	N2	3.81210100	−0.14870400	−0.10559200
	O3	3.73524400	−1.51122400	−0.17745200
	N4	2.42720300	−1.94786100	−0.14365200
	C5	1.68288300	−0.87493300	−0.03591600
	C6	0.23122400	−0.84078900	0.01650100
	N7	−0.43311200	0.32435600	−0.16591300
	C8	−1.72520800	−0.03870000	−0.06514500
	N9	−1.75713900	−1.38974900	0.17040100
	N10	−0.51462900	−1.91799900	0.22761700
	N11	2.30210000	1.67723600	0.13147700
	O12	2.00901300	2.04761300	1.25325100
	O13	2.42502300	2.37005200	−0.86162400
	N14	−2.74062100	0.85126100	−0.19553800
	N15	−3.98632500	0.38456900	−0.07364800
	O16	−4.90720200	1.20302900	−0.20020800
	O17	−4.23468800	−0.83928700	0.16204000
	H18	−2.61090500	−1.91550400	0.29959600

SCF Done: E(B3LYP, cation) = −298.0617301 a.u.

SCF Done: E(B3LYP, anion) = −967.2011174 a.u.

Table S152: Salt 152

Cartesian coordinates

	Atom	X	Y	Z
Cation	N1	0.65541700	−0.00411800	0.00005100
	N2	−0.75045600	−0.00232700	−0.00003900
	H3	0.99718400	0.96411800	−0.00012100
	H4	1.05415400	−0.45557600	0.83700000
	H5	1.05425800	−0.45589200	−0.83667700
	H6	−1.21844400	−0.89213100	−0.00011000

	H7	−1.22187800	0.88459500	−0.00017400
Anion	C1	0.00000000	0.00000000	0.00000000
	N2	0.00000000	1.44647000	0.00000000
	O3	1.07697200	2.05098400	0.00000000
	O4	−1.07697200	2.05098400	0.00000000
	N5	−1.25268000	−0.72323500	0.00000000
	O6	−1.23771800	−1.95817700	0.00000000
	O7	−2.31469000	−0.09280700	0.00000000
	N8	1.25268000	−0.72323500	0.00000000
	O9	1.23771800	−1.95817700	0.00000000
	O10	2.31469000	−0.09280700	0.00000000

SCF Done: E(B3LYP, cation) = −112.2418137 a.u.

SCF Done: E(B3LYP, anion) = −653.654036 a.u.
